# Supplementary material for: Discovery of Novel Cinnamide Fungicidal Leads with Optical Hydroxyl Side Chain
Source: Molecules. 2022 Aug 17;27(16):5259. doi: 10.3390/molecules27165259 (PMC9414950; doi:10.3390/molecules27165259)
Supplement: Supplementary file 1 [file molecules-27-05259-s001.zip › molecules-1853154-supplementary.pdf]

Supplementary Materials for Molecules

Discovery of Novel Cinnamide Fungicidal Leads with Optical Hydroxyl Side Chain

Weiwei Wang [1], Jiazhen Jiang [1], Zhenhua Zhang [1] and Mingan Wang [1,*]

Innovation Center of Pesticide Research, Department of Applied Chemistry, China Agricutural University,

Beijing 100193, China

*Corresponding author: Prof. Dr. Mingan Wang

E-mail: wangma@cau.edu.cn

Tel: +86-13621078240

Fax: +86-01-62737732

Contents

1. NMR spectra for the intermediates and title compounds..S2-S50

2. HPLC profiles of five representative compounds……………………………………..………..S51-S58

1. NMR spectra for the intermediates and title compounds

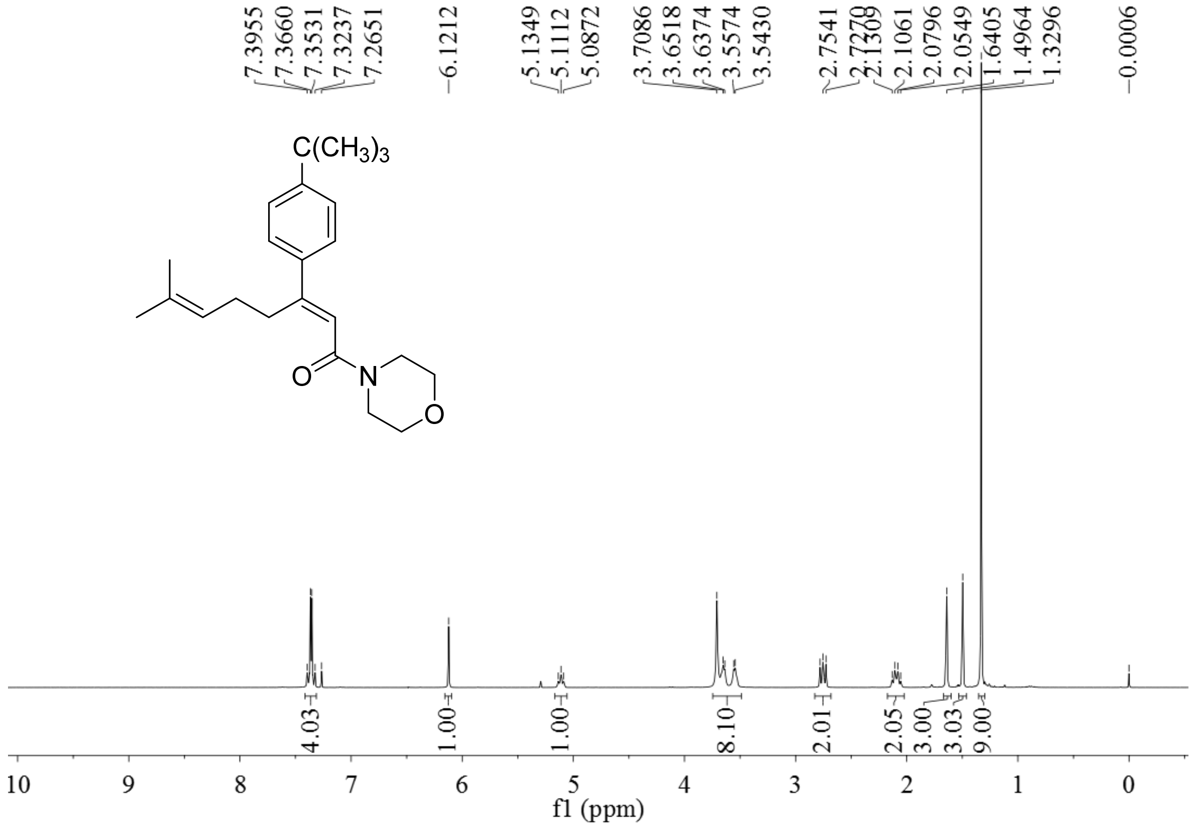

Figure S1. ¹H NMR of Compound **10a**

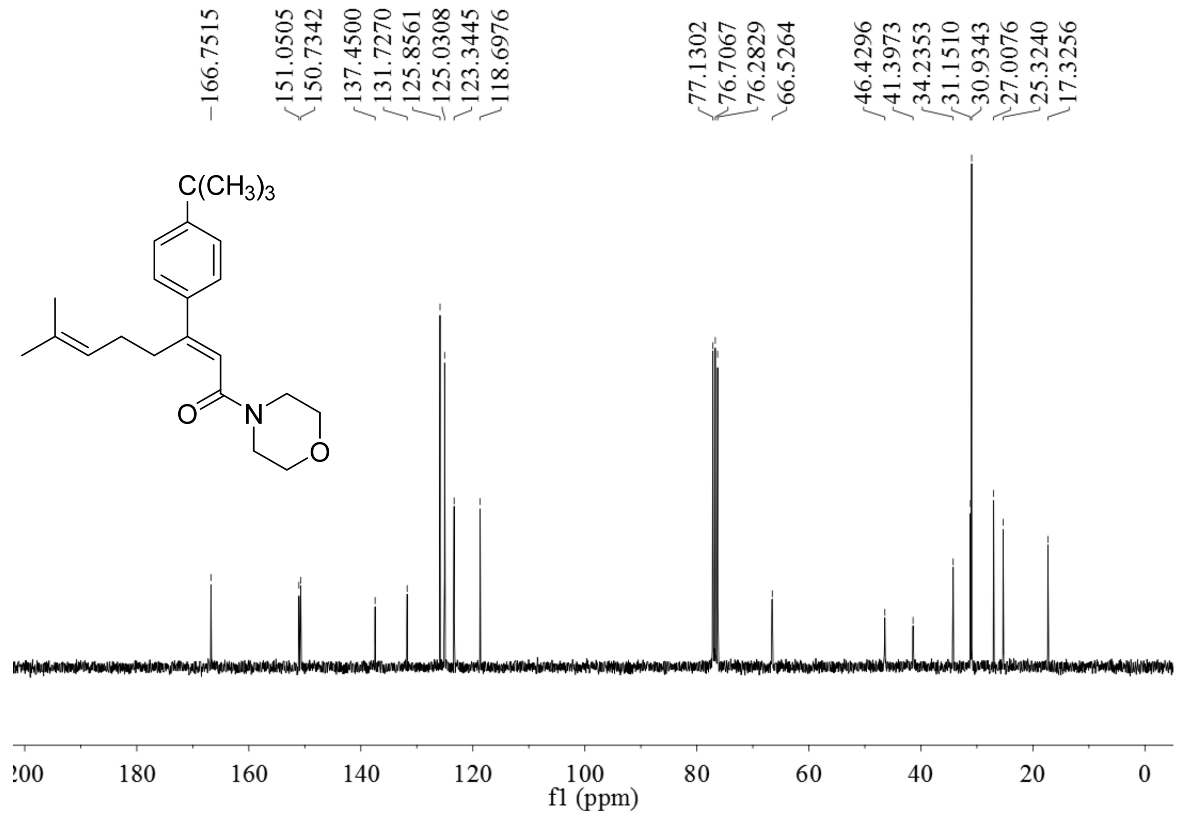

Figure S2. ¹³C NMR spectrum of **10a**

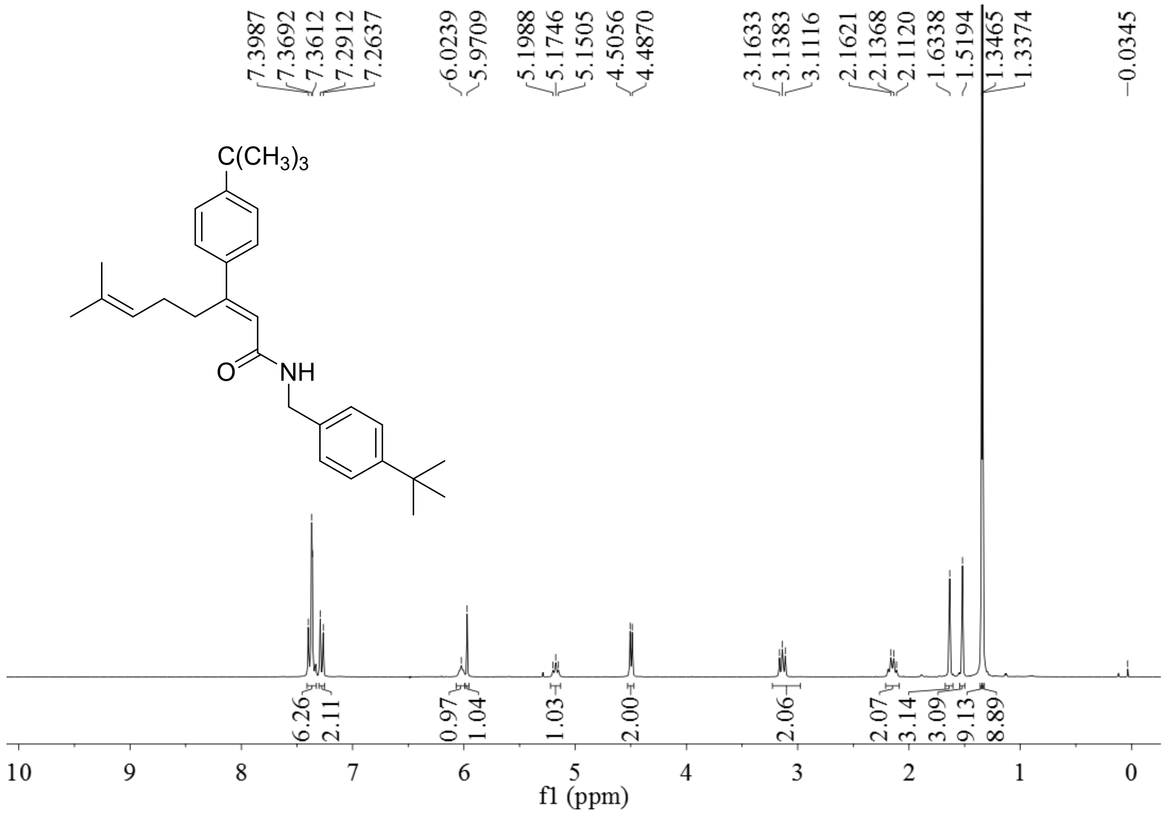

Figure S3. ^{1}H NMR of Compound **10b**

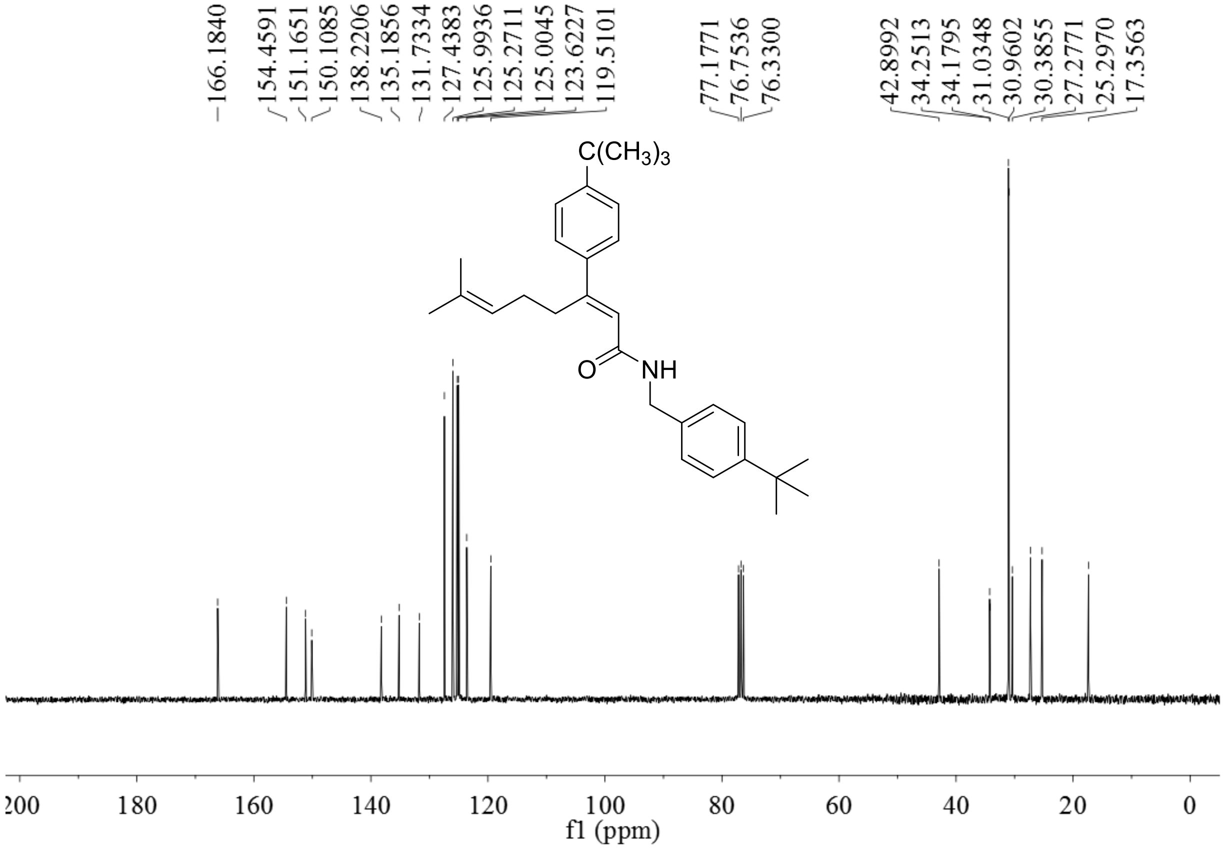

Figure S4. ^{13}C NMR spectrum of **10b**

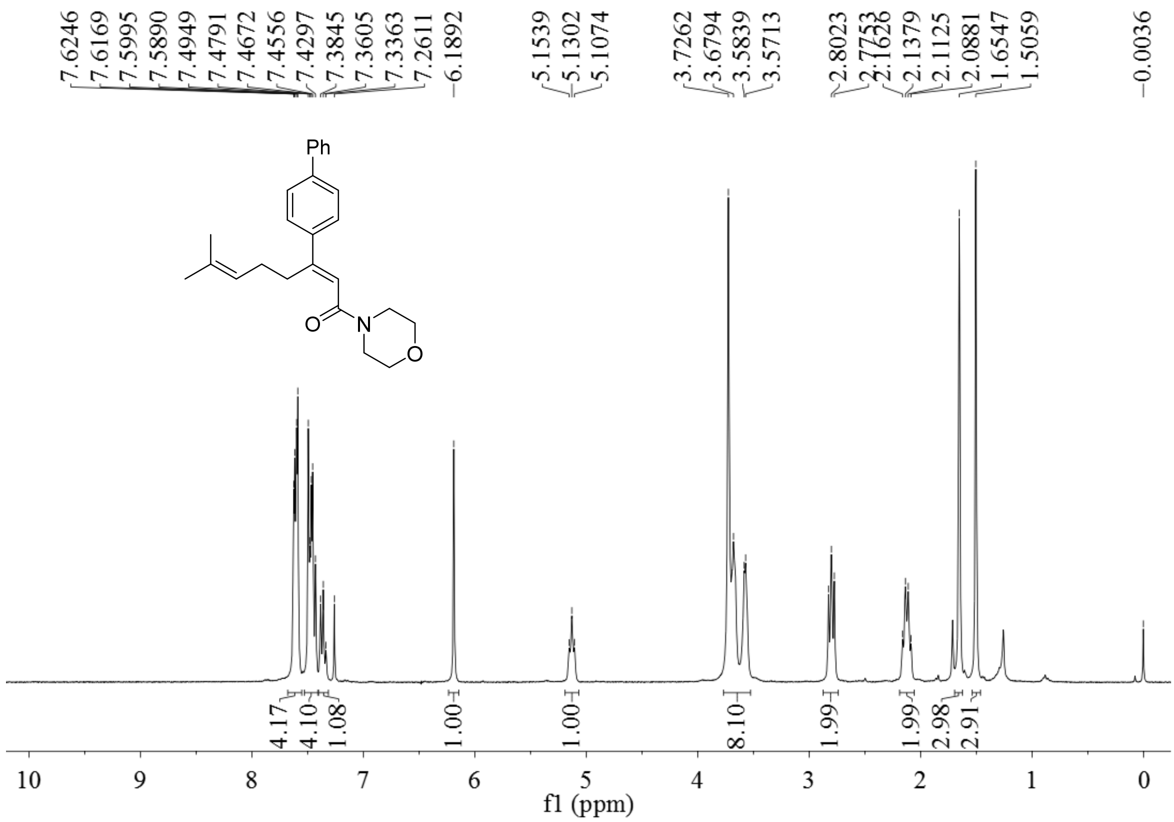

Figure S5. ^{1}H NMR of Compound **10c**

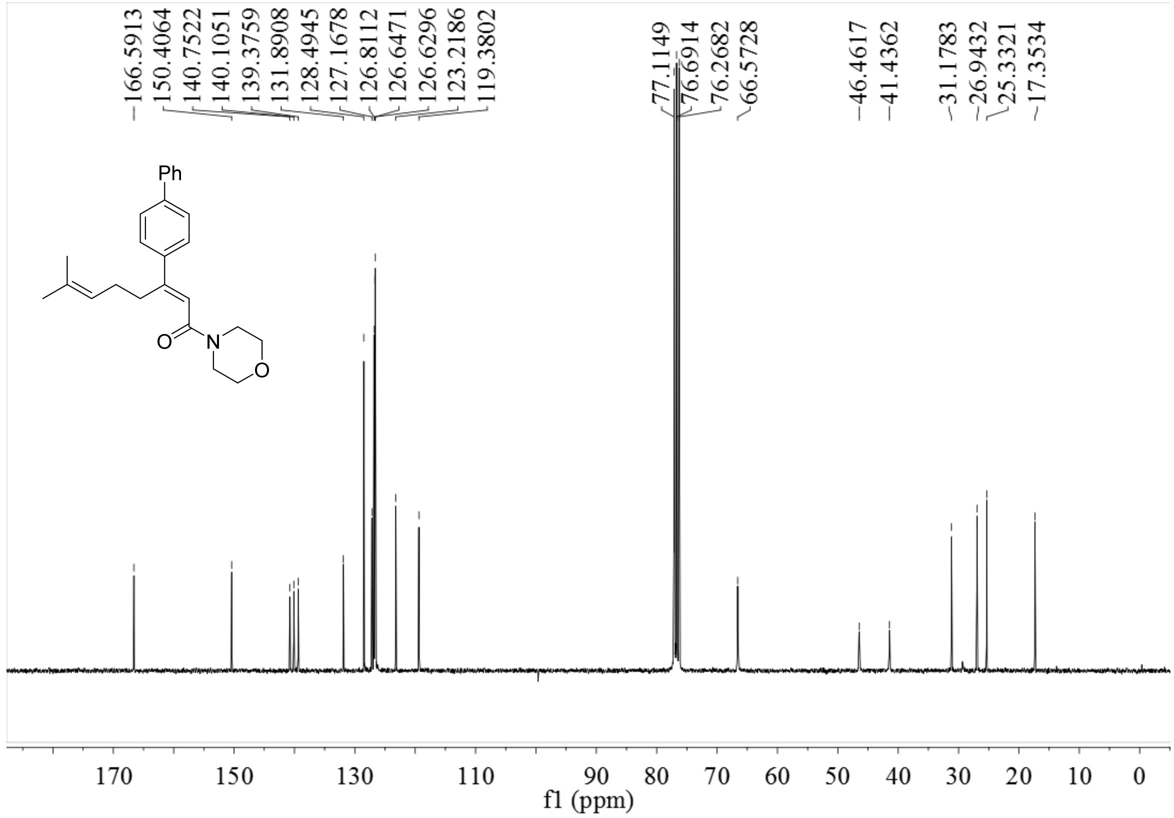

Figure S6. ^{13}C NMR spectrum of **10c**

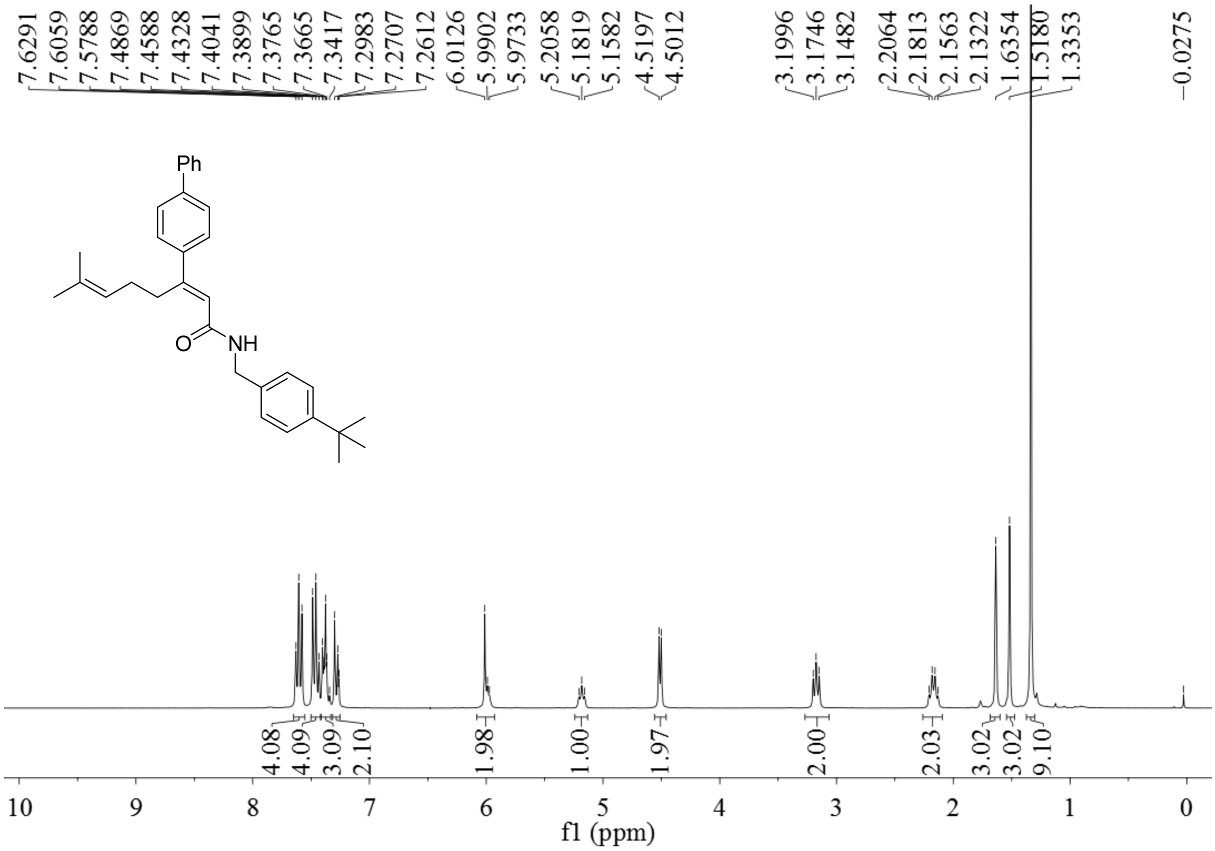

Figure S7. ^{1}H NMR of Compound **10d**

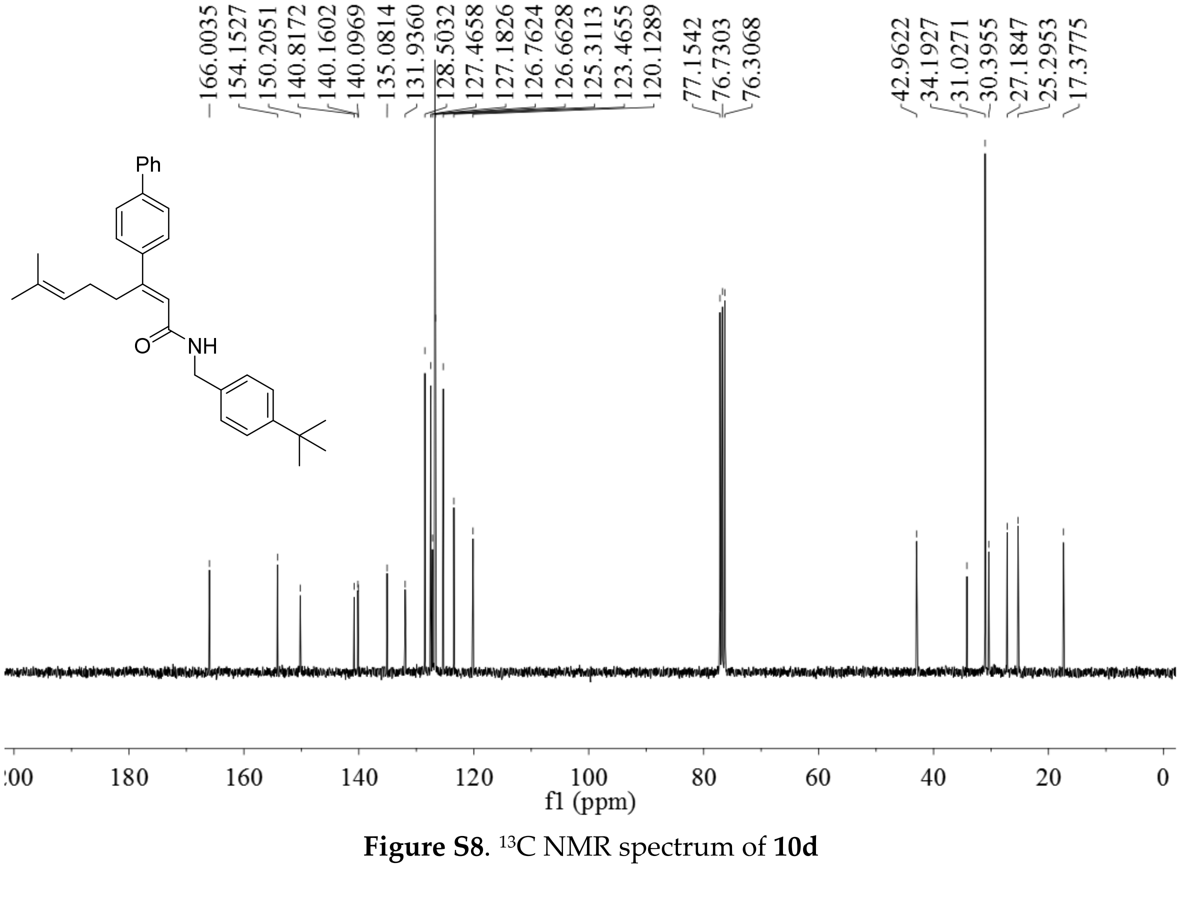

Figure S8. ^{13}C NMR spectrum of **10d**

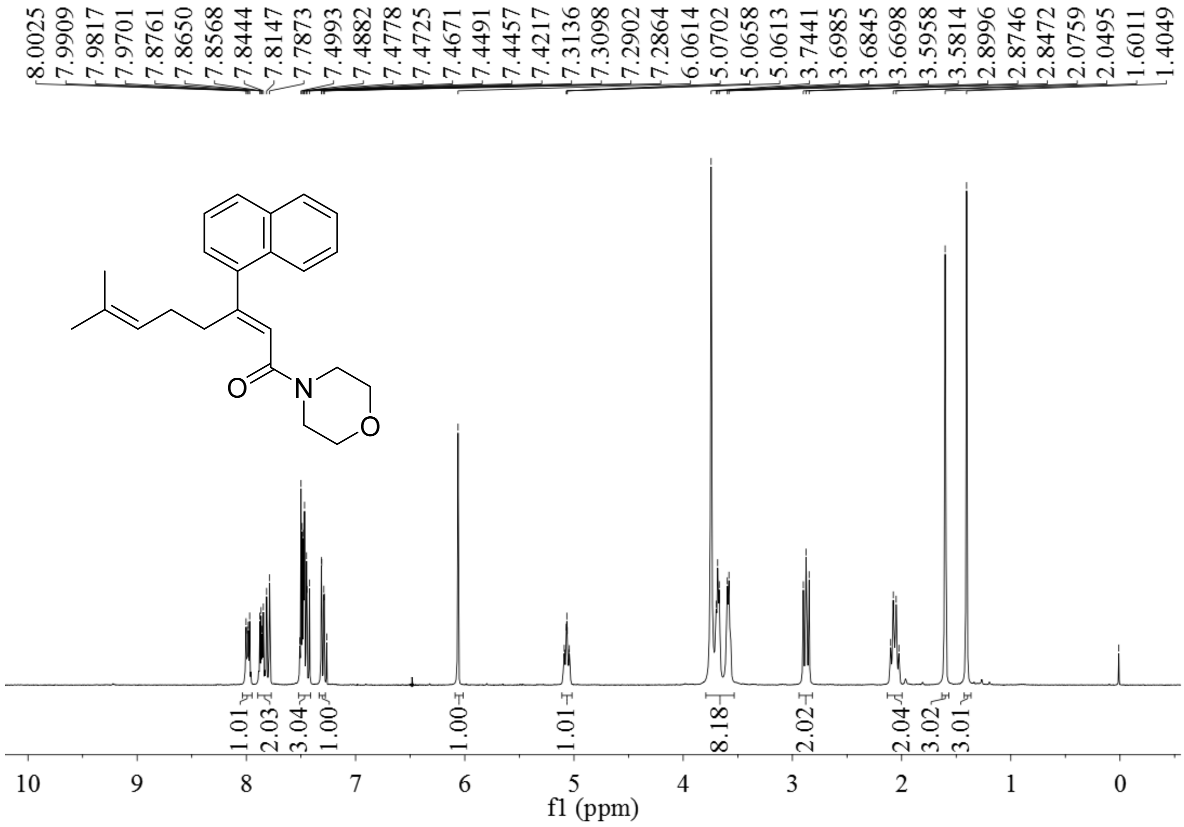

Figure S9. ^{1}H NMR of Compound **10e**

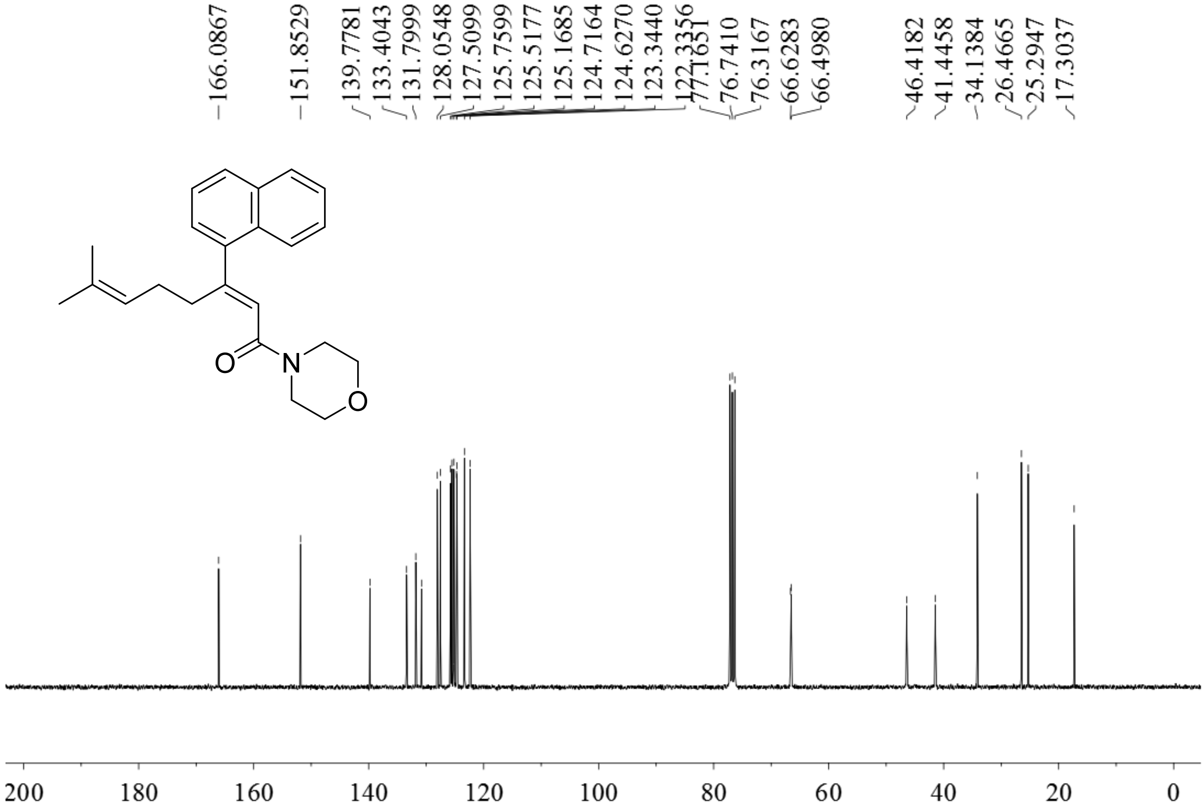

Figure S10. ^{13}C NMR spectrum of **10e**

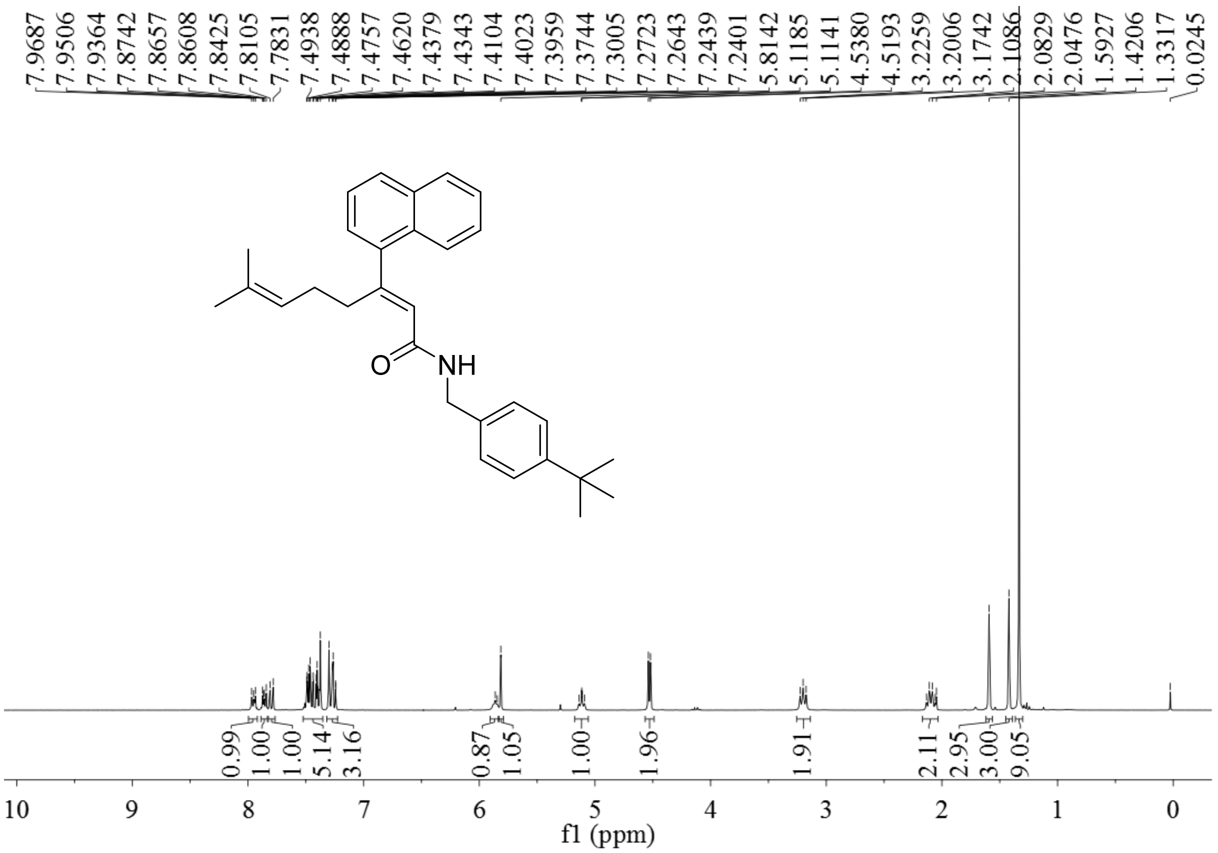

Figure S11. ^{1}H NMR of Compound **10f**

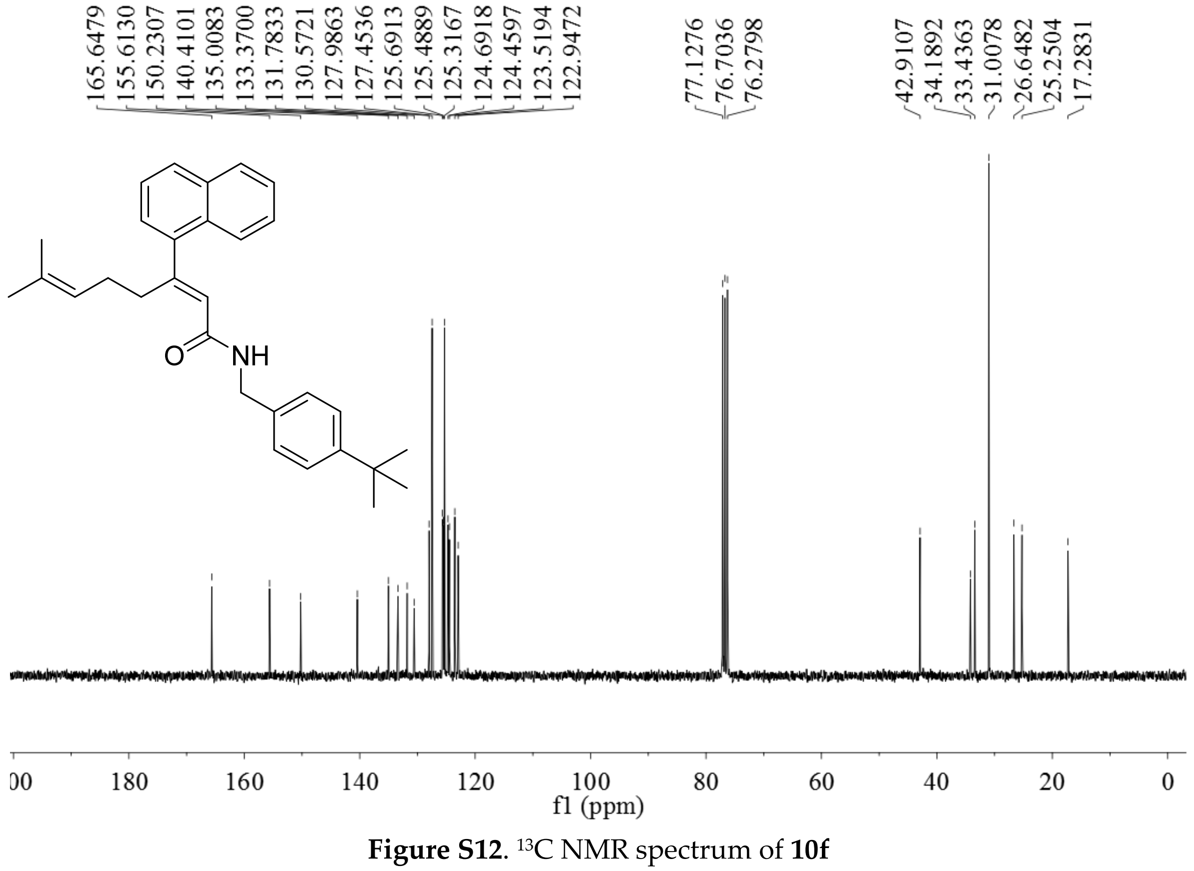

Figure S12. ^{13}C NMR spectrum of **10f**

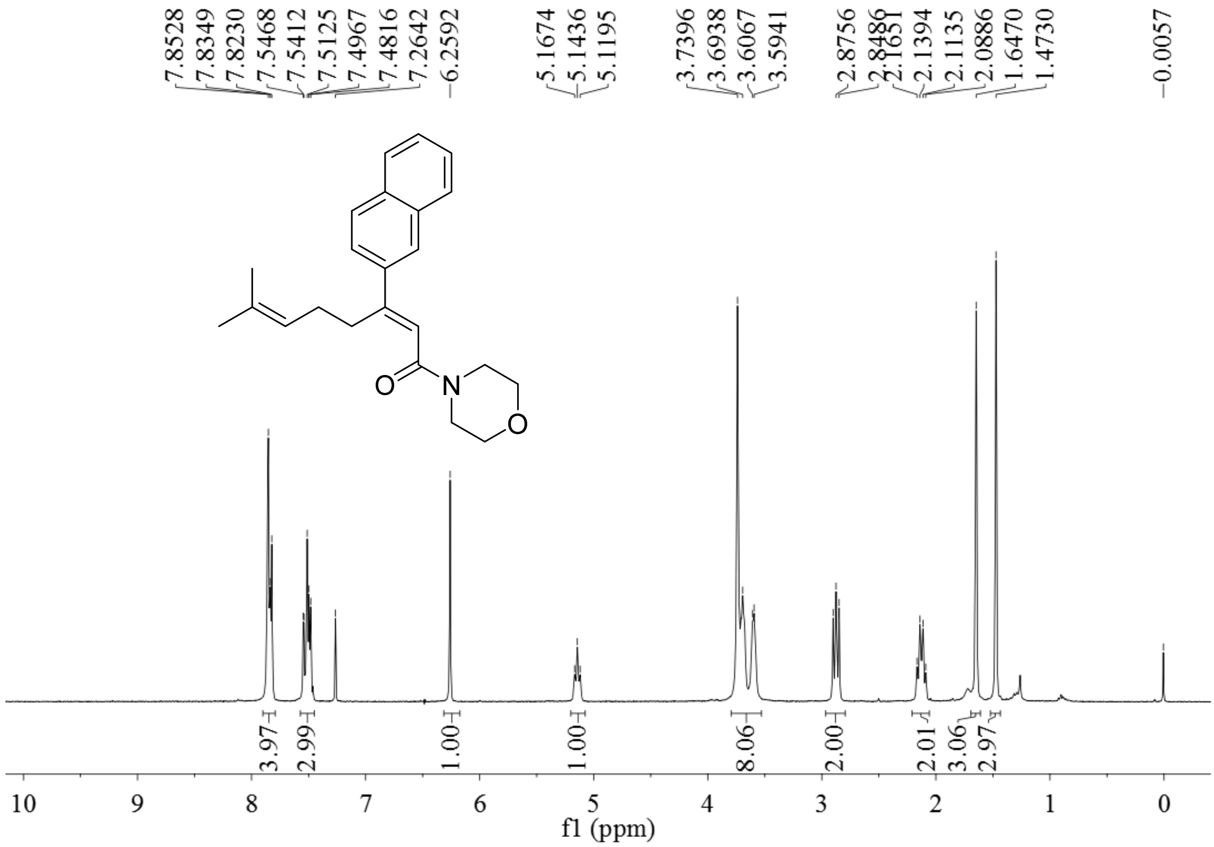

Figure S13. ^{1}H NMR of Compound **10g**

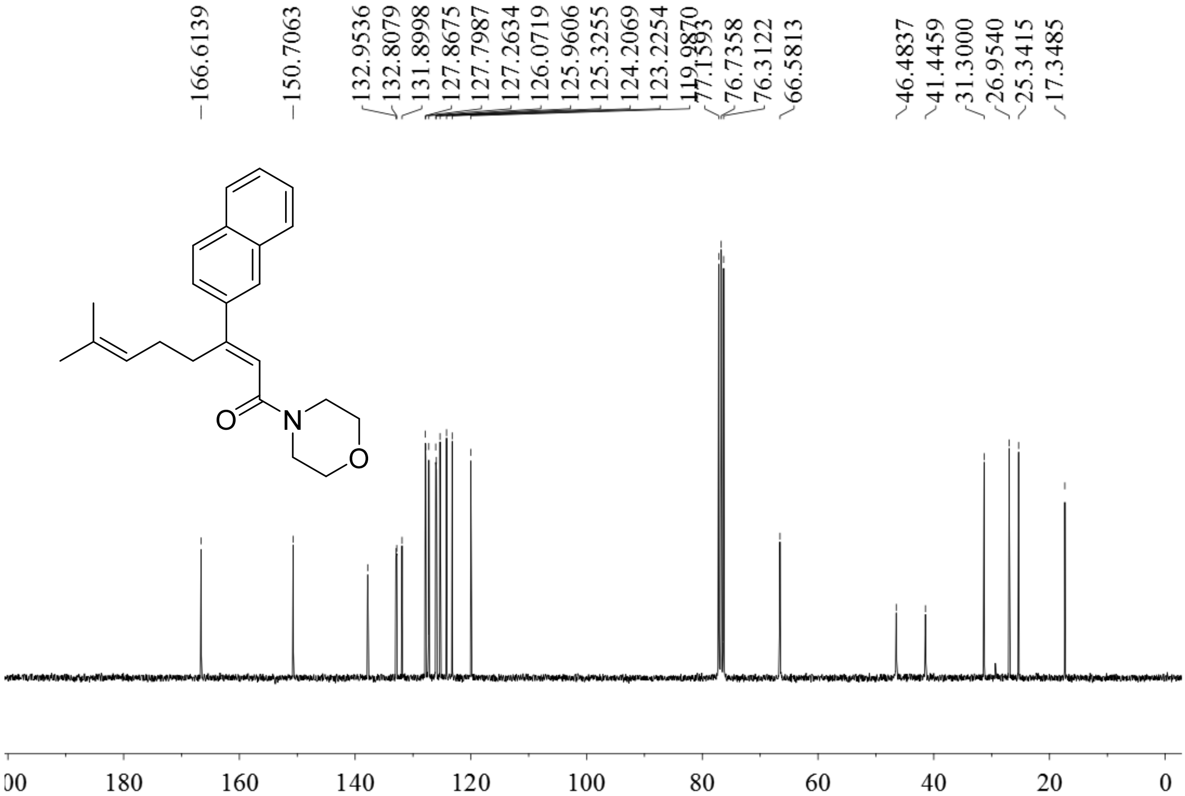

Figure S14. ^{13}C NMR spectrum of **10g**

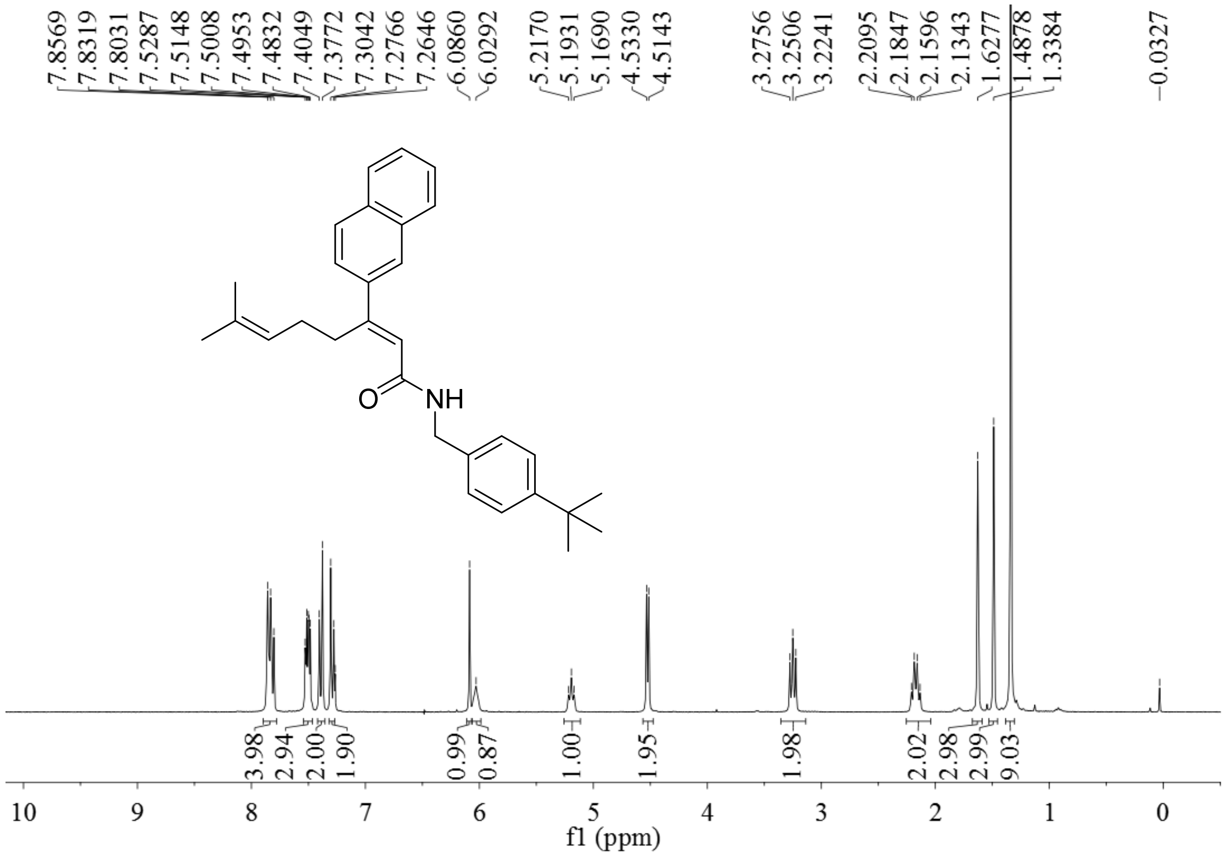

Figure S15. ^{1}H NMR of Compound **10h**

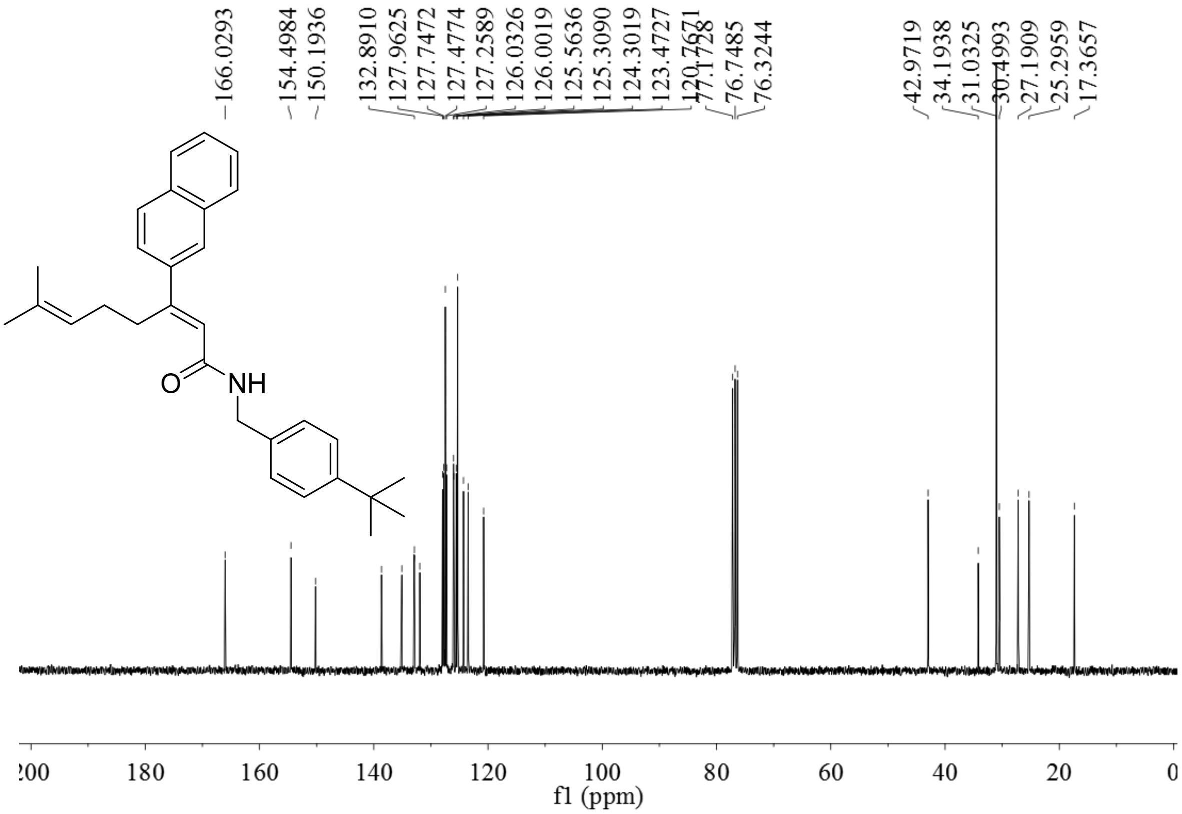

Figure S16. ^{13}C NMR spectrum of **10h**

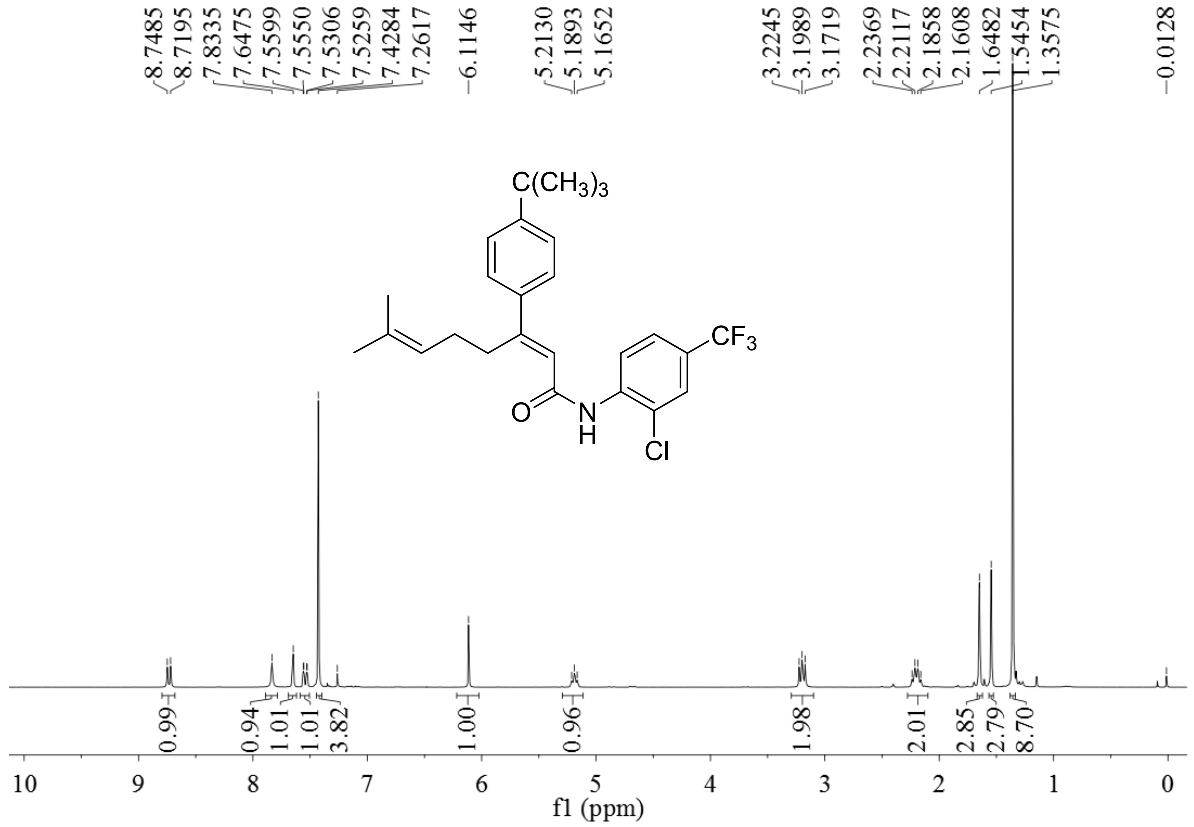

Figure S17. ^{1}H NMR of Compound **10i**

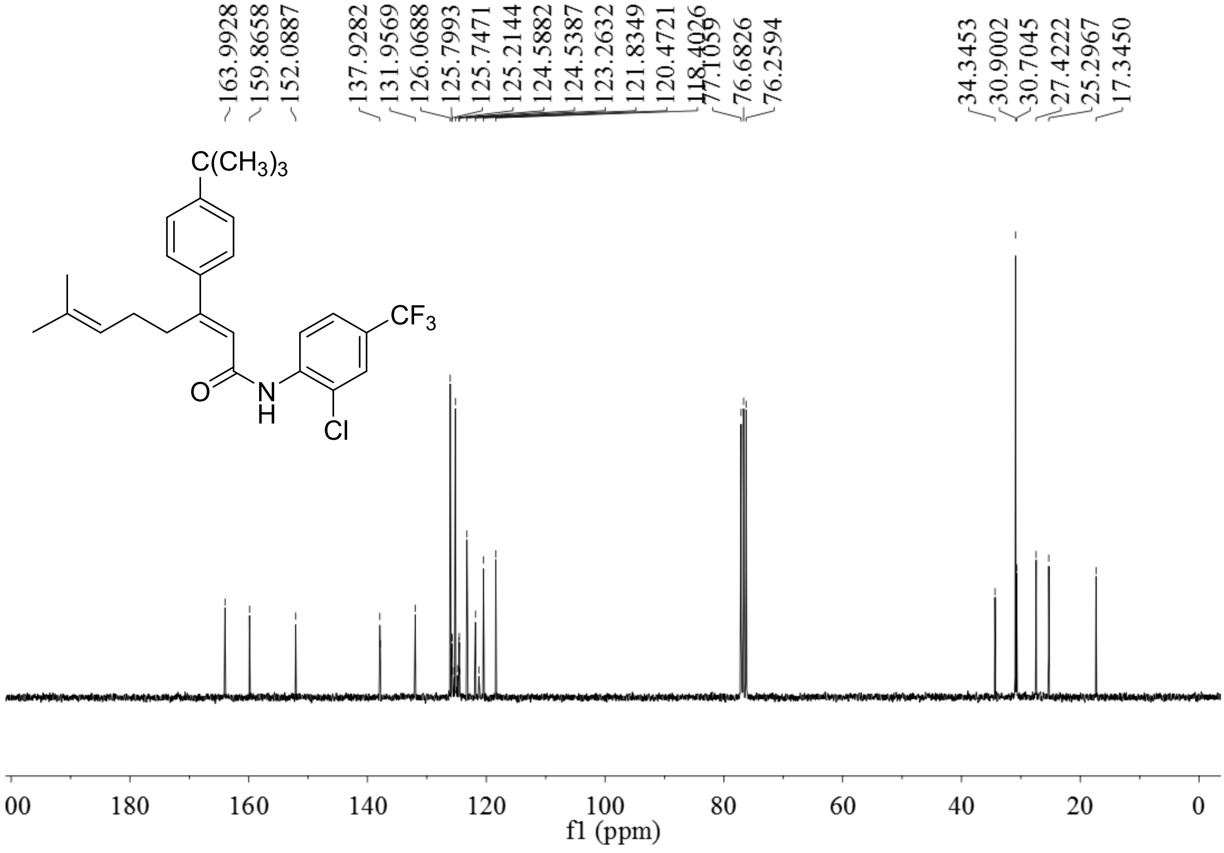

Figure S18. ^{13}C NMR spectrum of **10i**

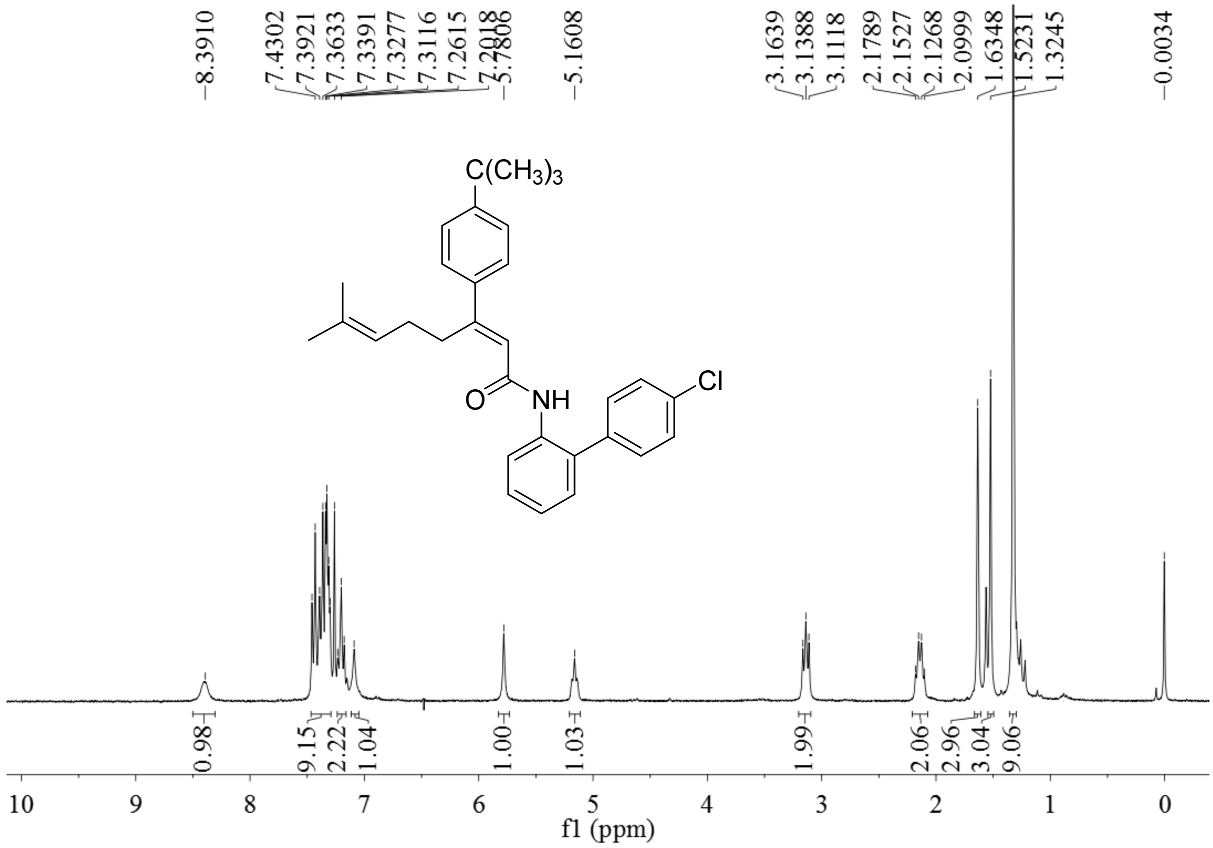

Figure S19. ^{1}H NMR of Compound **10j**

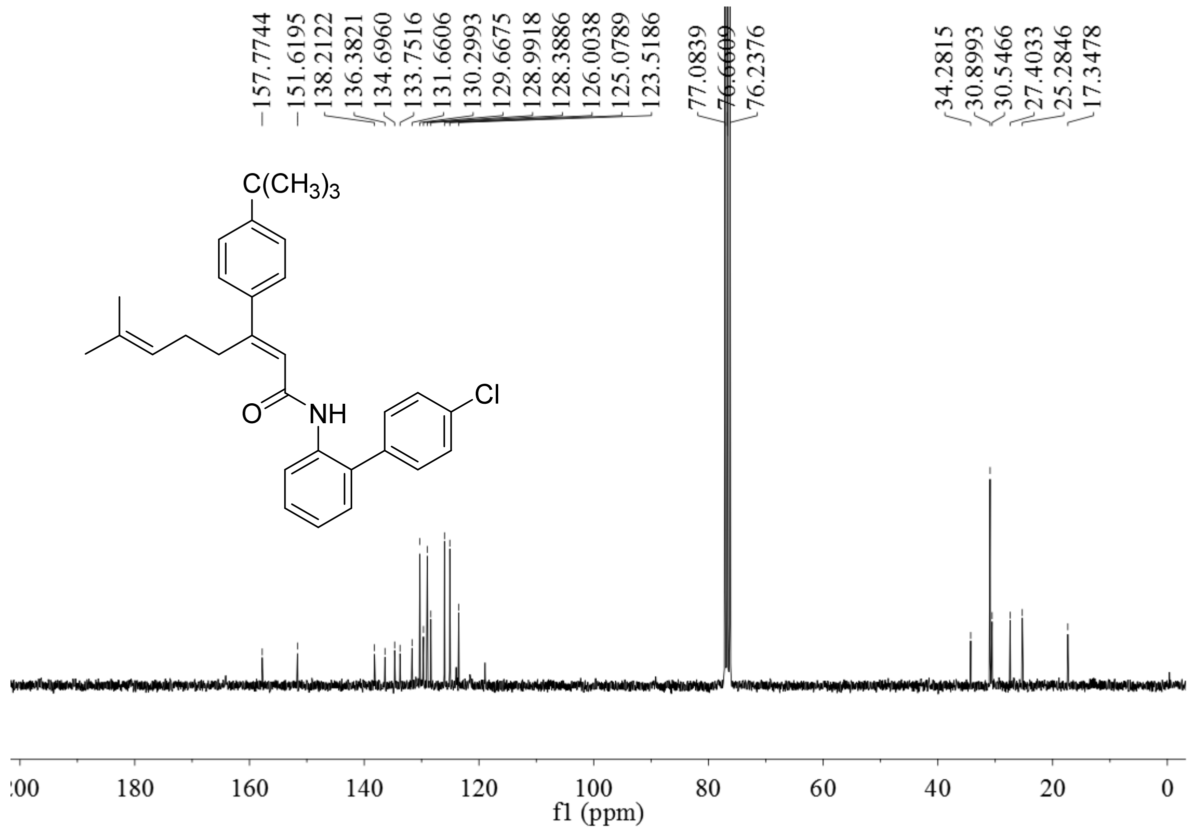

Figure S20. ^{13}C NMR spectrum of **10j**

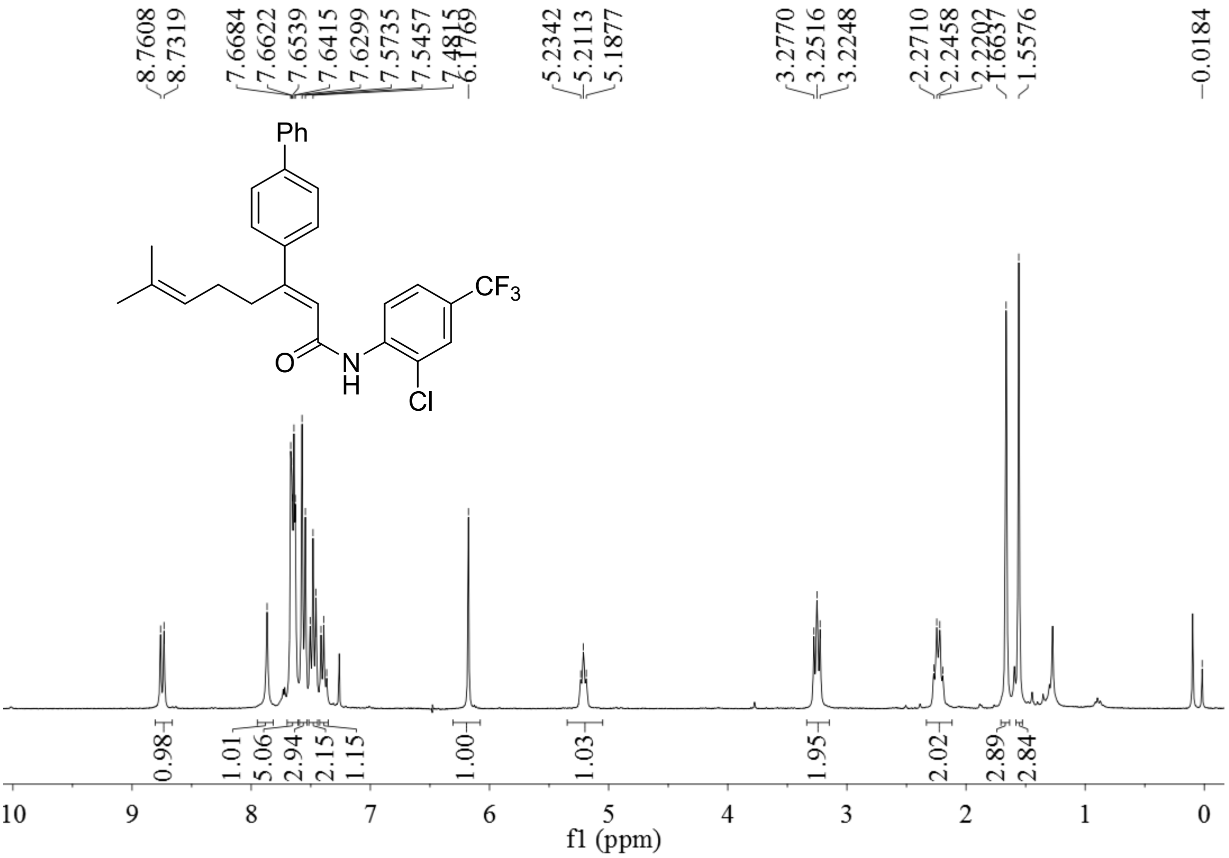

Figure S21. ^{1}H NMR of Compound **10k**

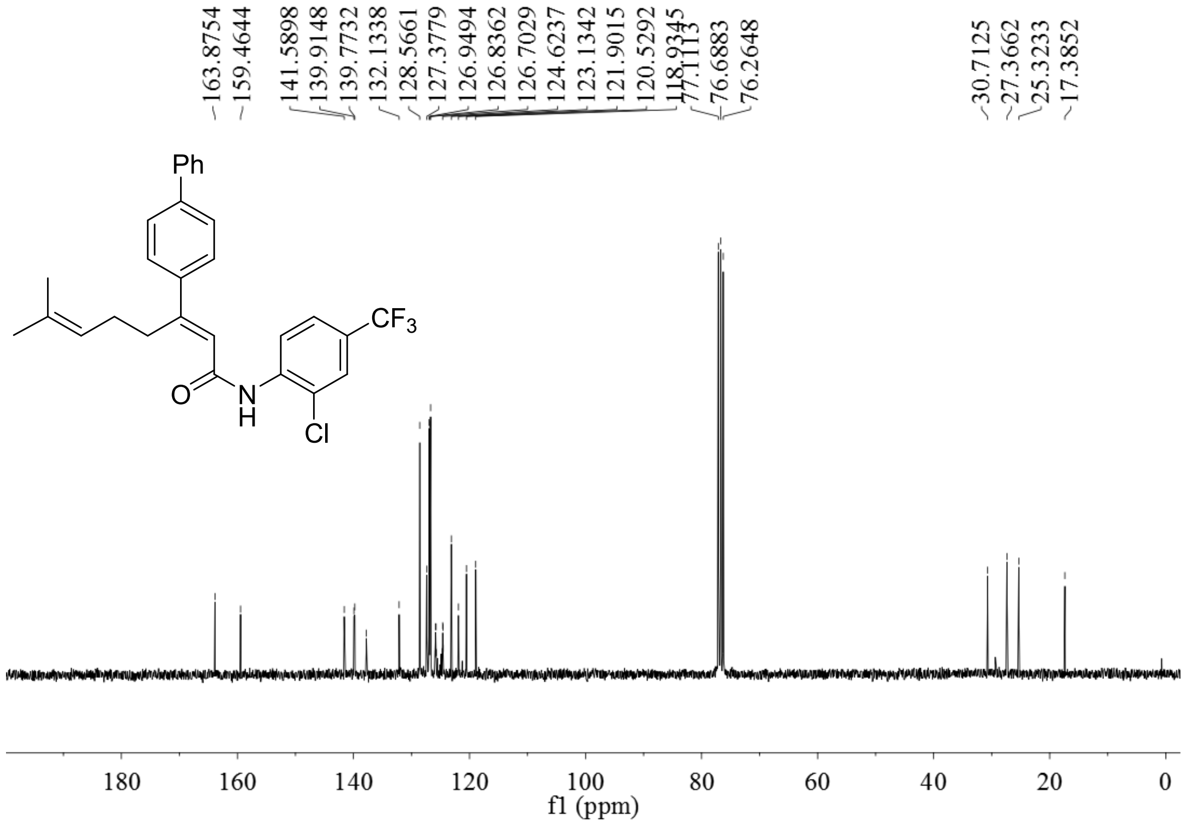

Figure S22. ^{13}C NMR spectrum of **10k**

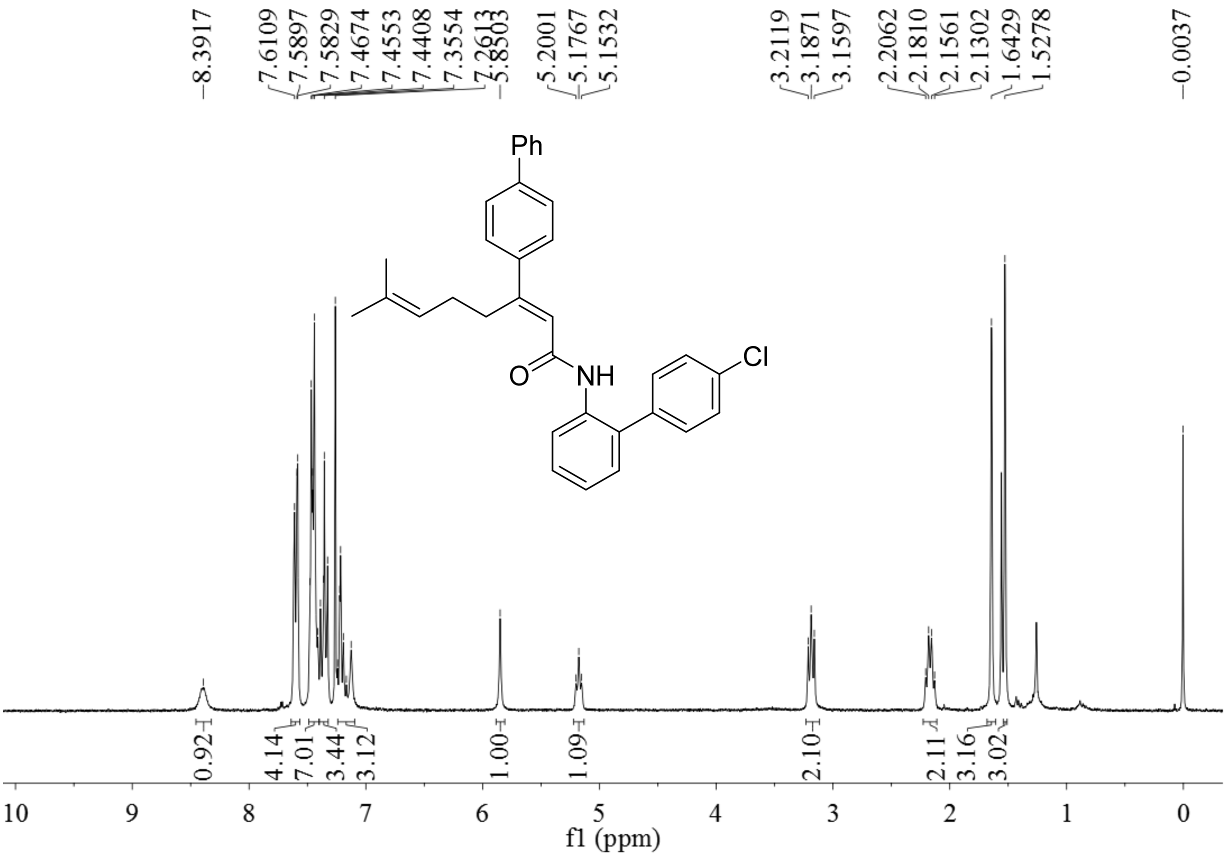

Figure S23. ^{1}H NMR of Compound **10l**

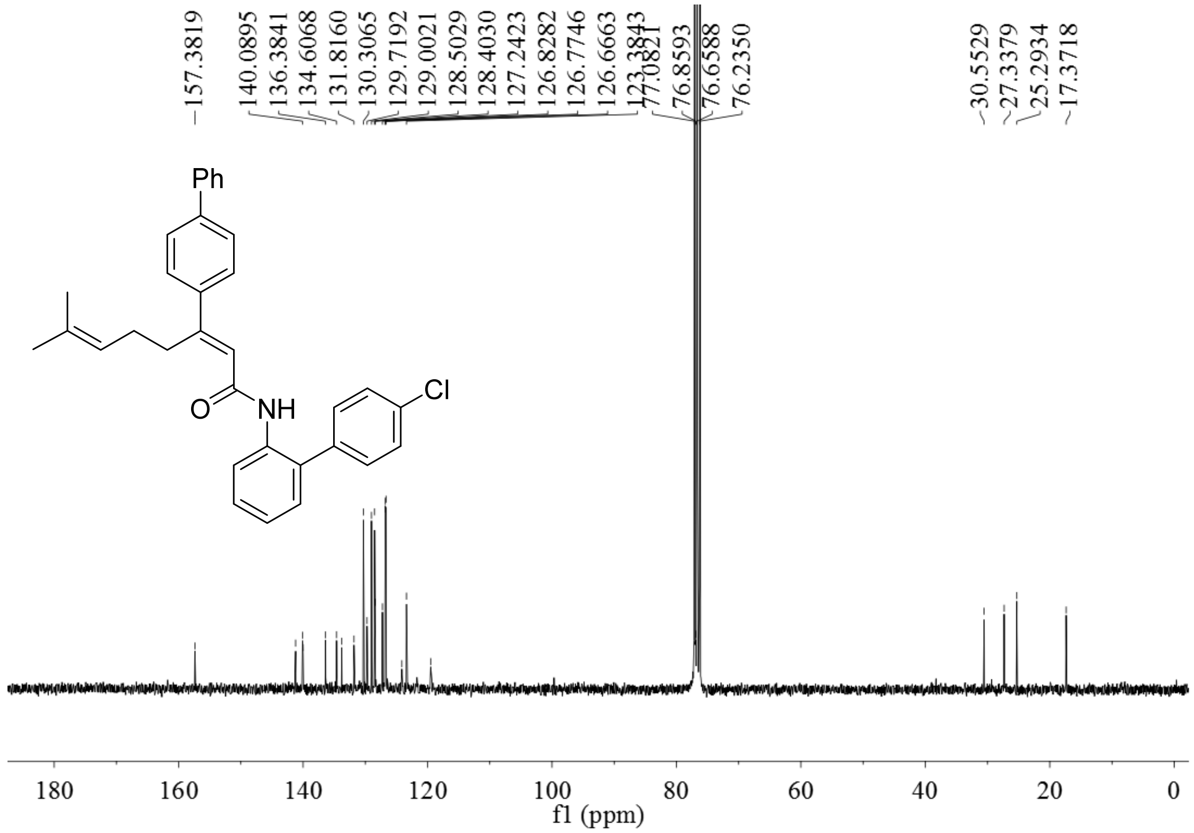

Figure S24. ^{13}C NMR spectrum of **10l**

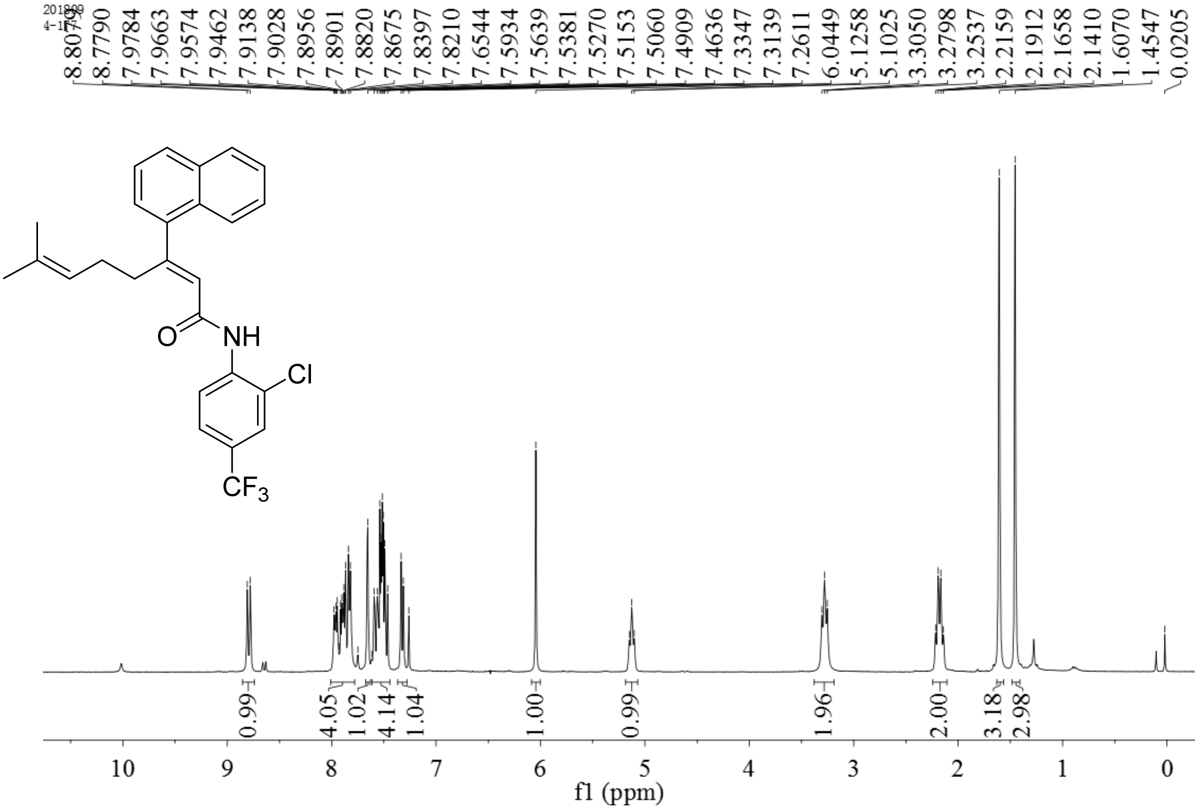

Figure S25. ^{1}H NMR of Compound **10m**

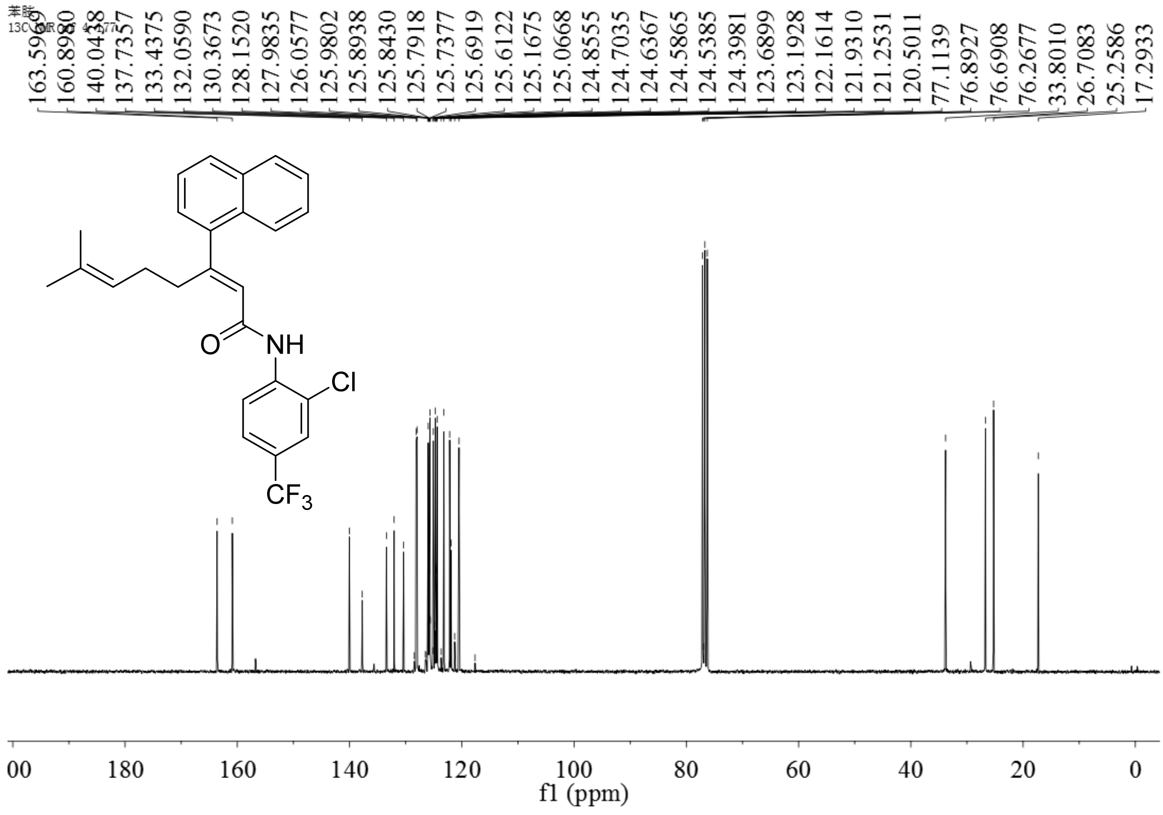

Figure S26. ^{13}C NMR spectrum of **10m**

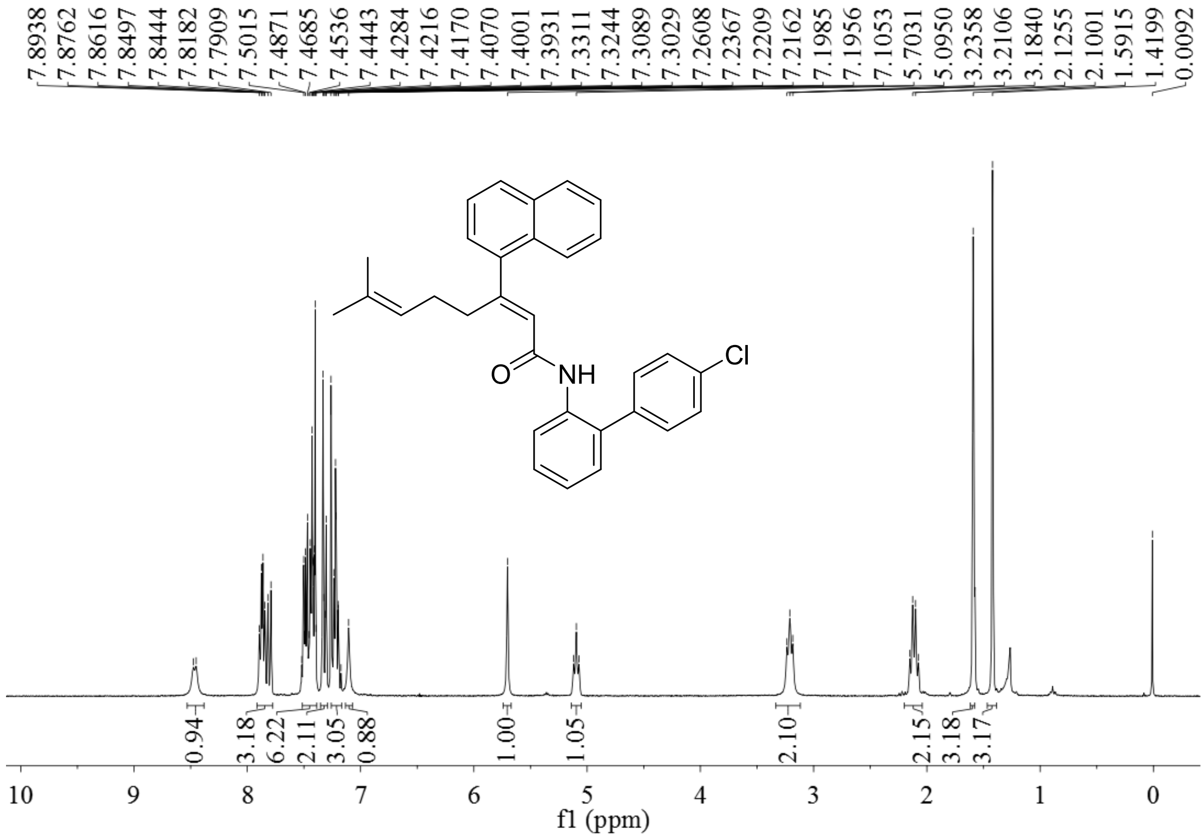

Figure S27. ^{1}H NMR of Compound **10n**

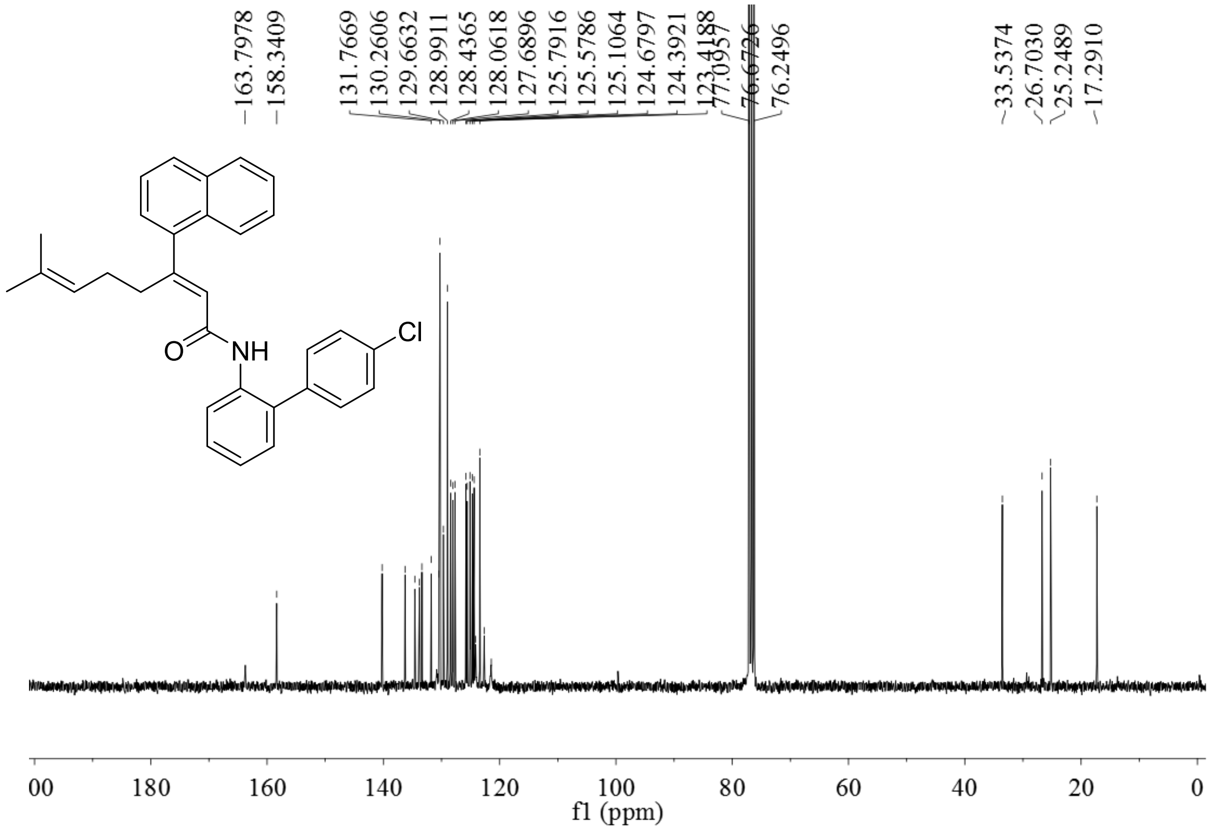

Figure S28. ^{13}C NMR spectrum of **10n**

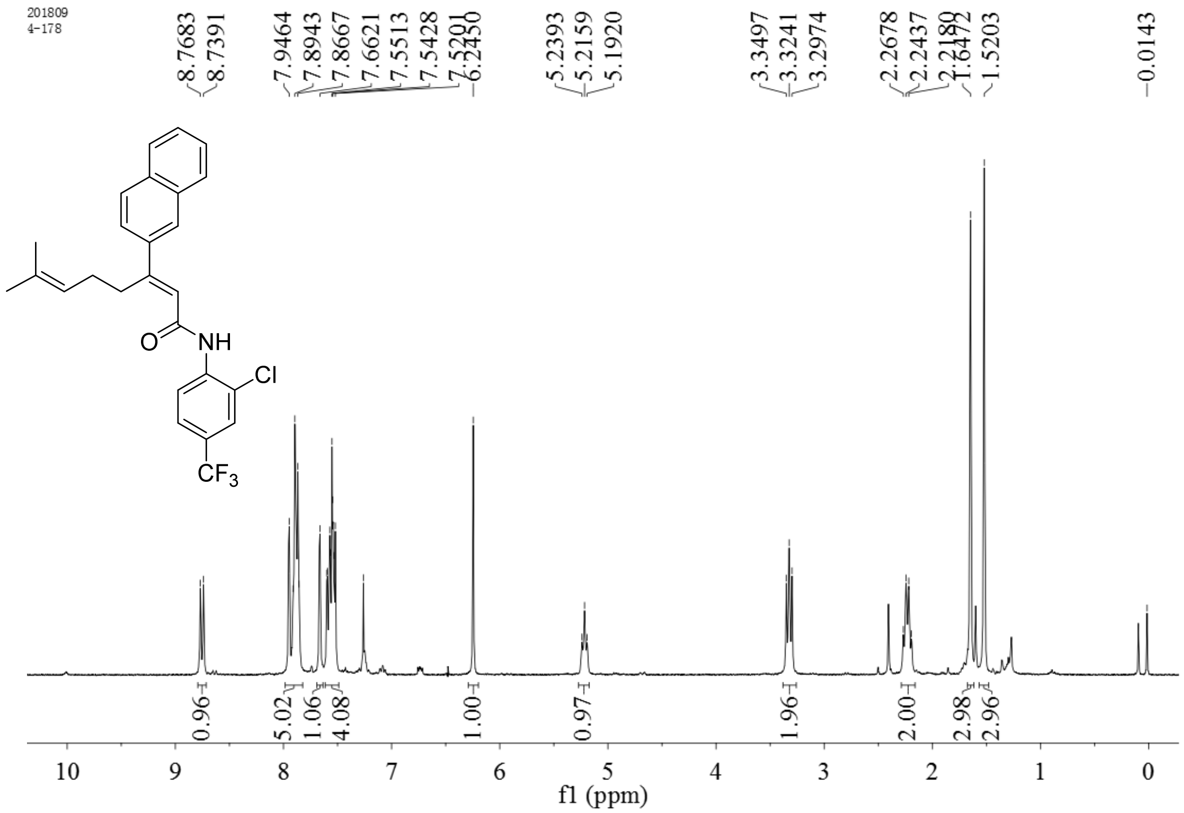

Figure S29. ^{1}H NMR of Compound **10o**

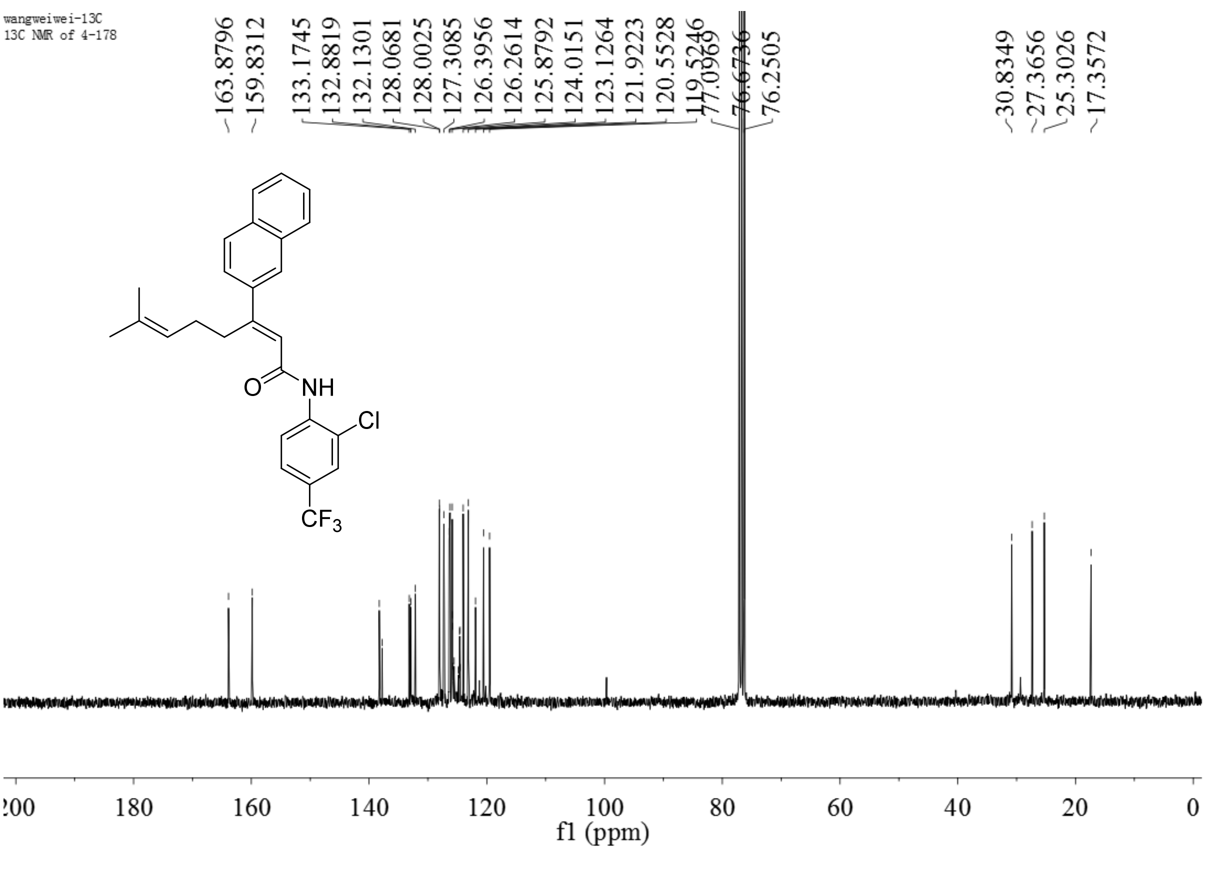

Figure S30. ^{13}C NMR spectrum of **10o**

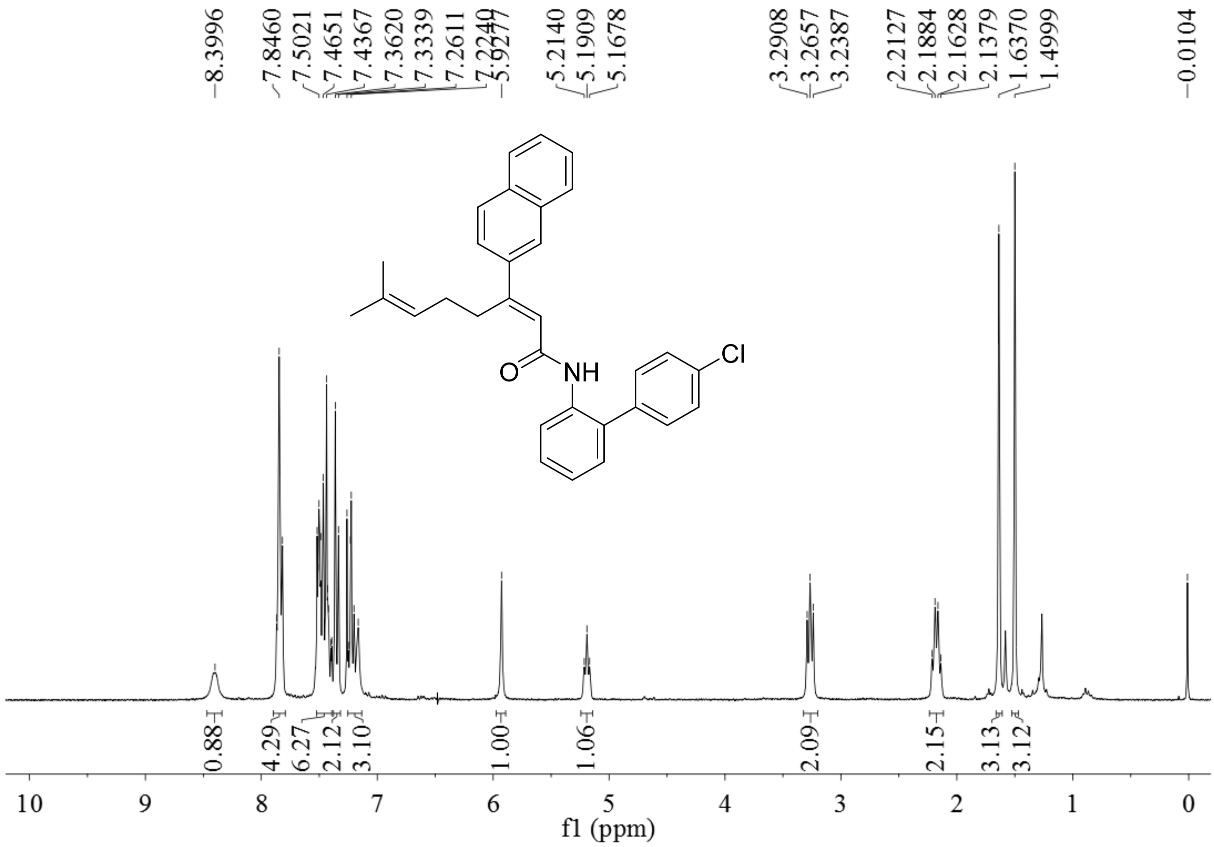

Figure S31. ^{1}H NMR of Compound **10p**

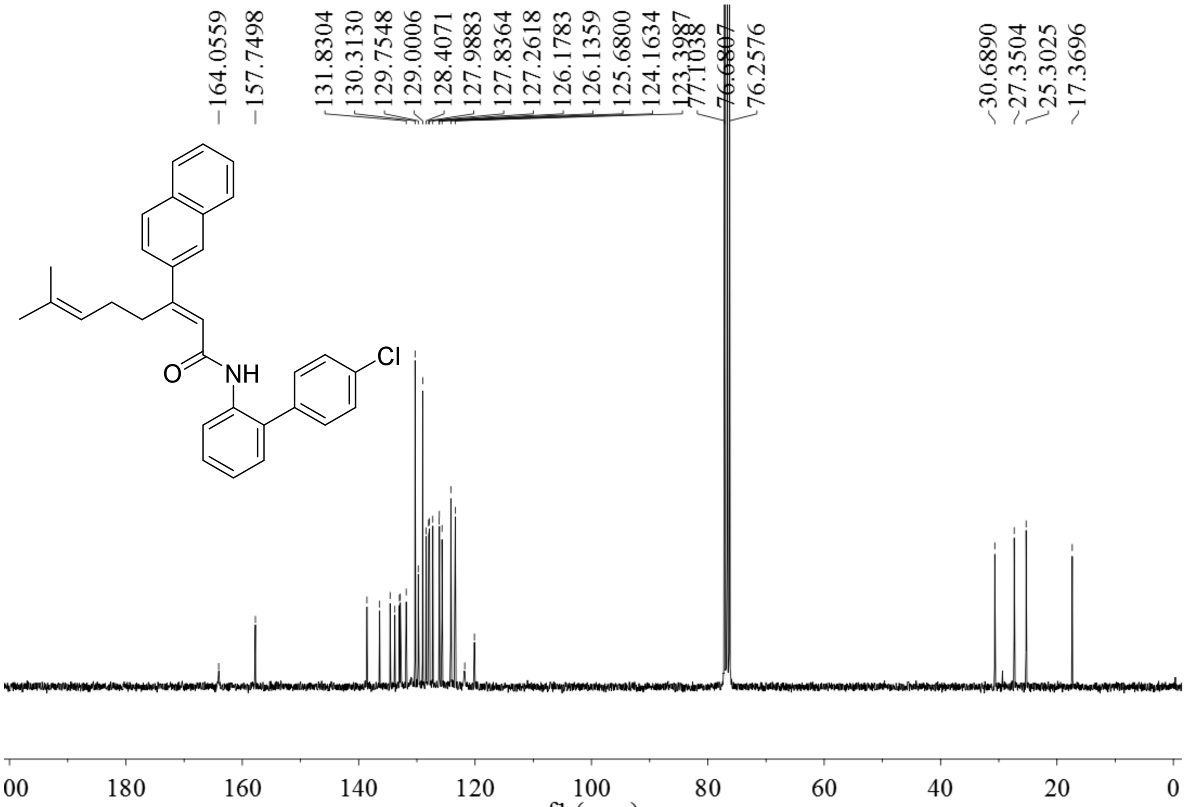

Figure S32. ^{13}C NMR spectrum of **10p**

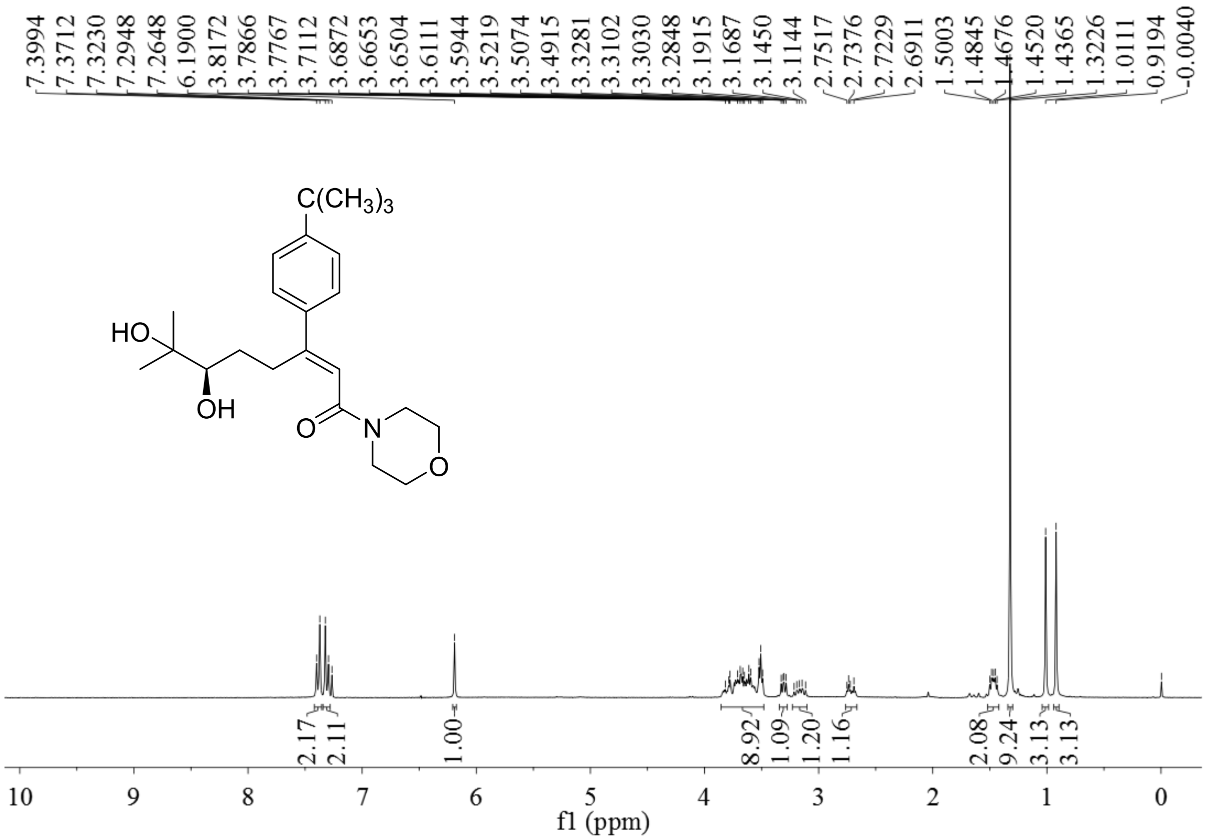

Figure S33. ^{1}H NMR of Compound (*R*)-**11a**

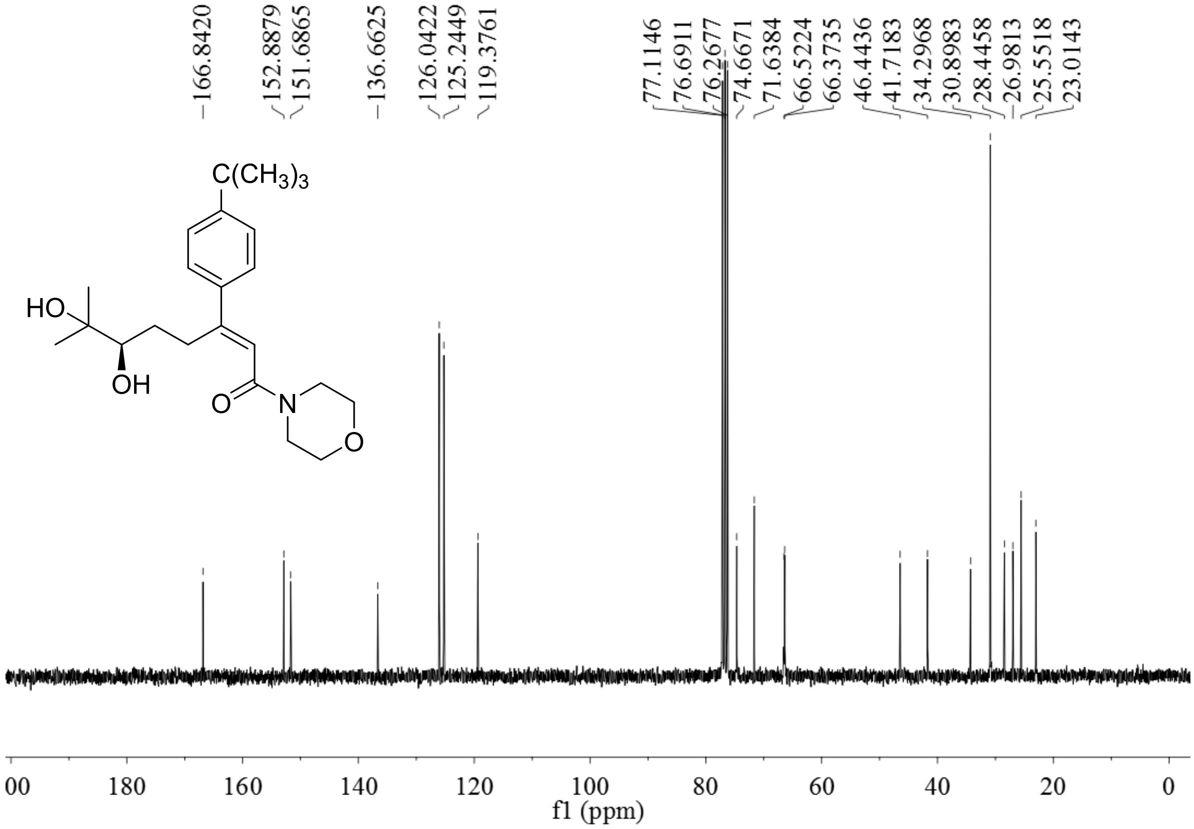

Figure S34. ^{13}C NMR spectrum of (*R*)-**11a**

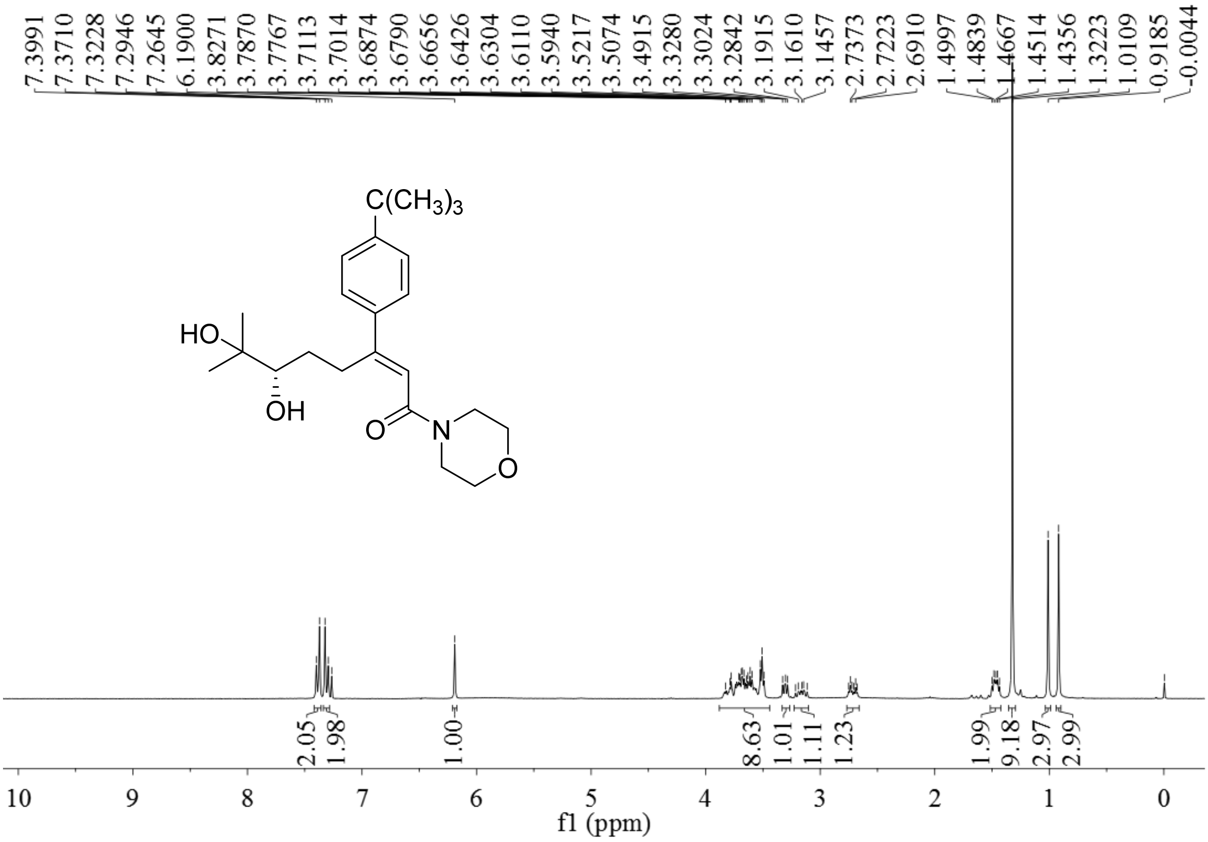

Figure S35. ^{1}H NMR of Compound (*S*)-**11a**

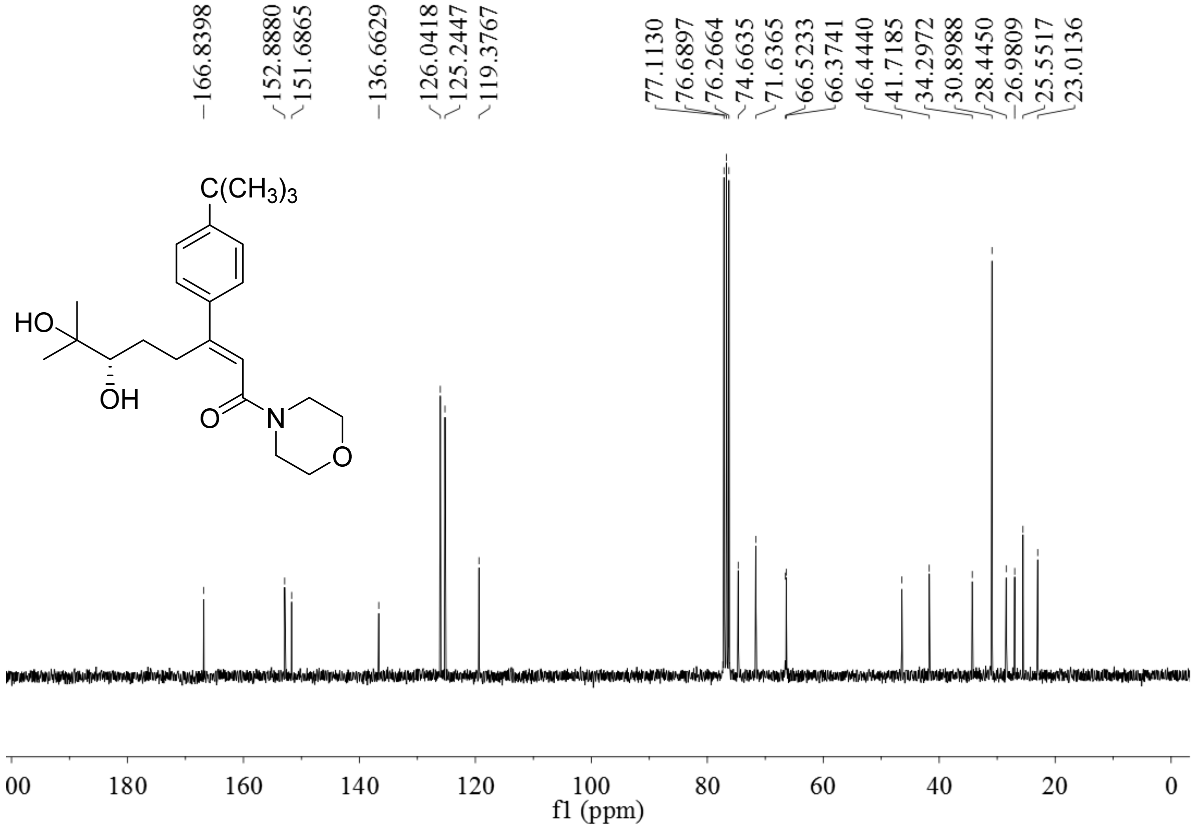

Figure S36. ^{13}C NMR spectrum of (*S*)-**11a**

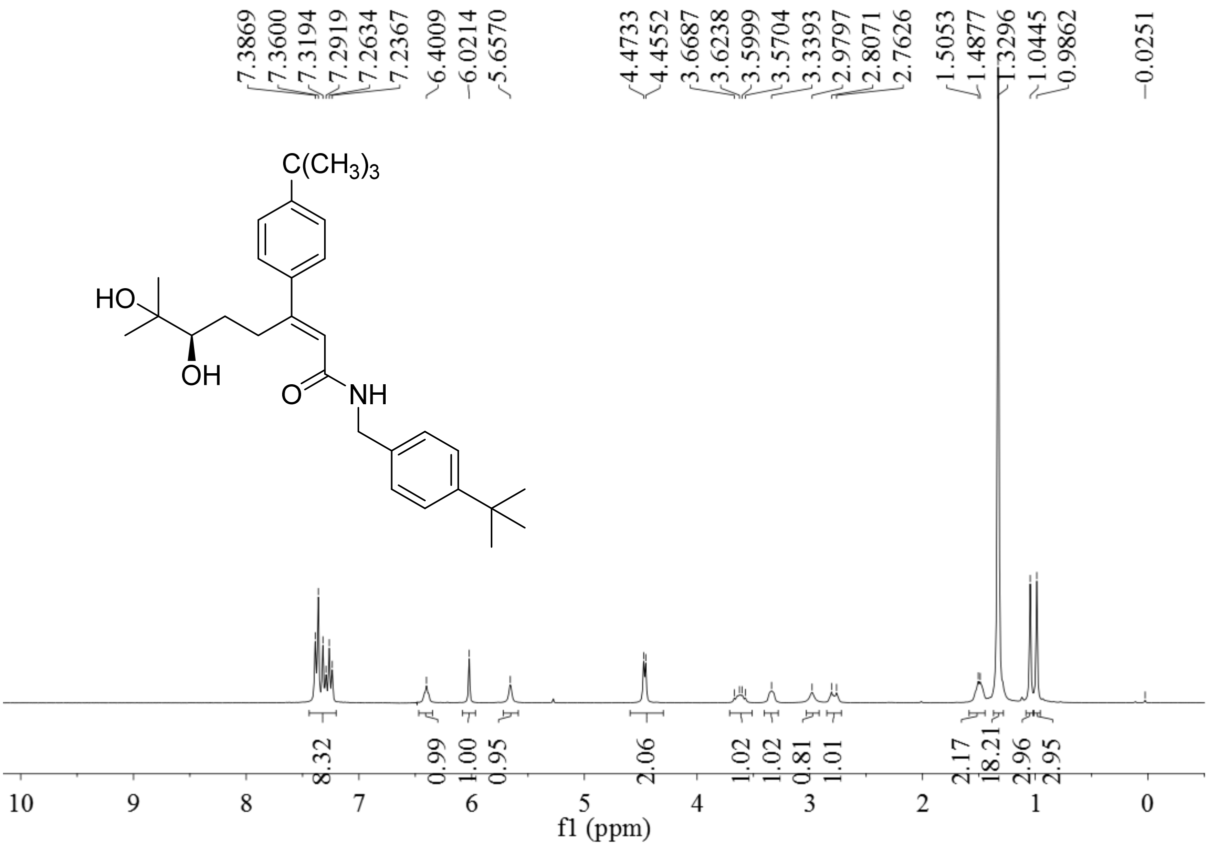

Figure S37. ^{1}H NMR of Compound (*R*)-**11b**

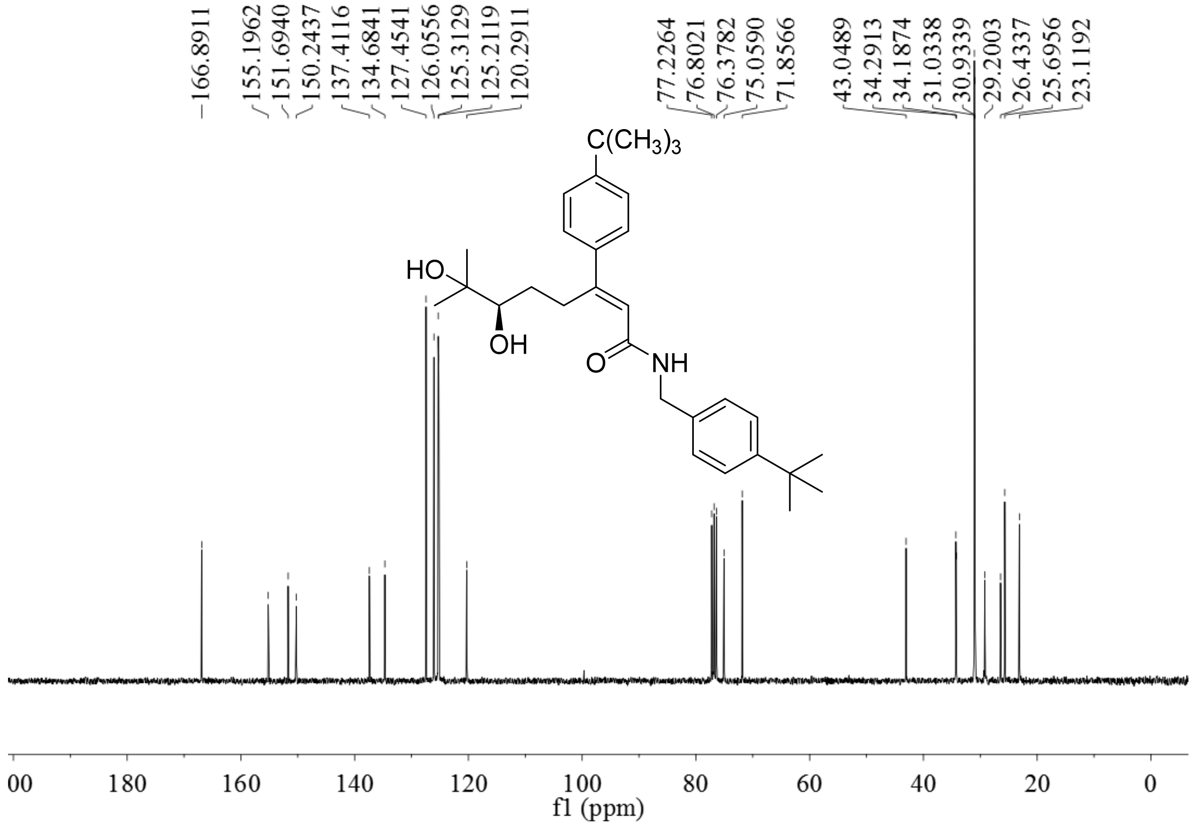

Figure S38. ^{13}C NMR spectrum of (*R*)-**11b**

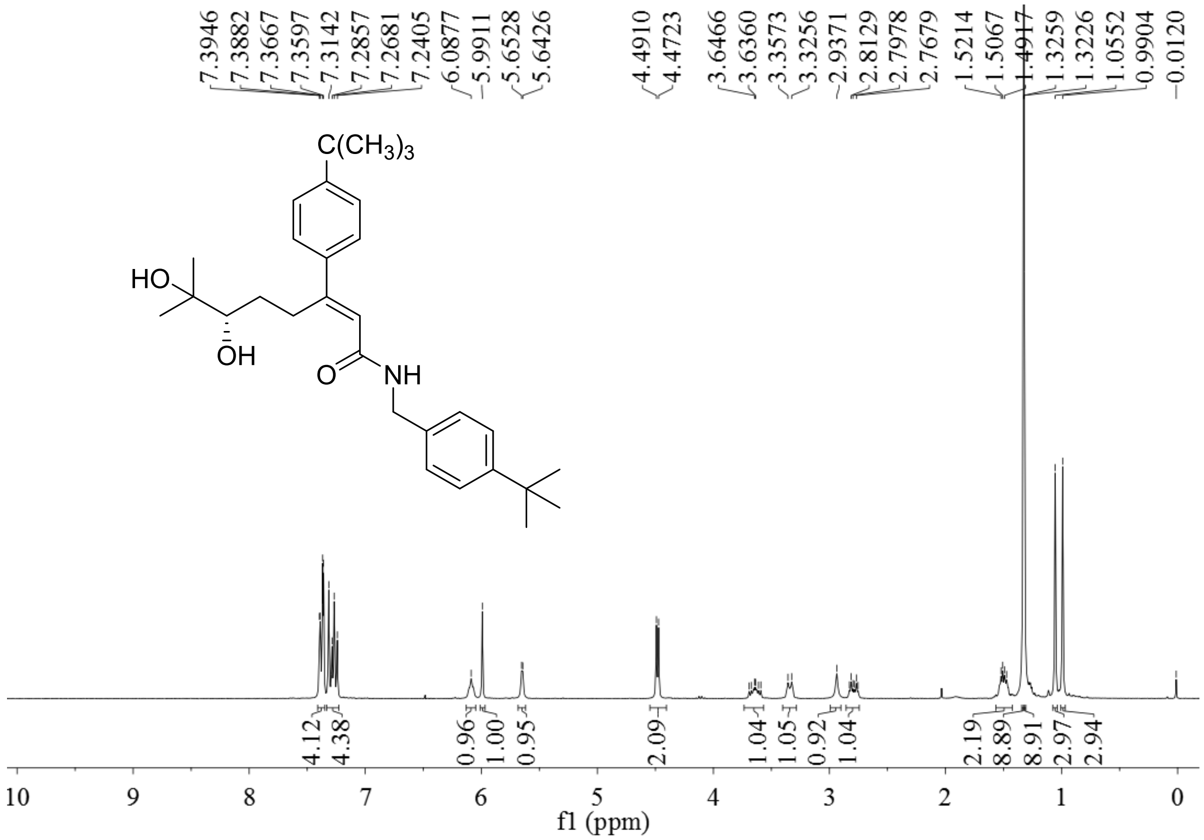

Figure S39. ^{1}H NMR of Compound (*S*)-**11b**

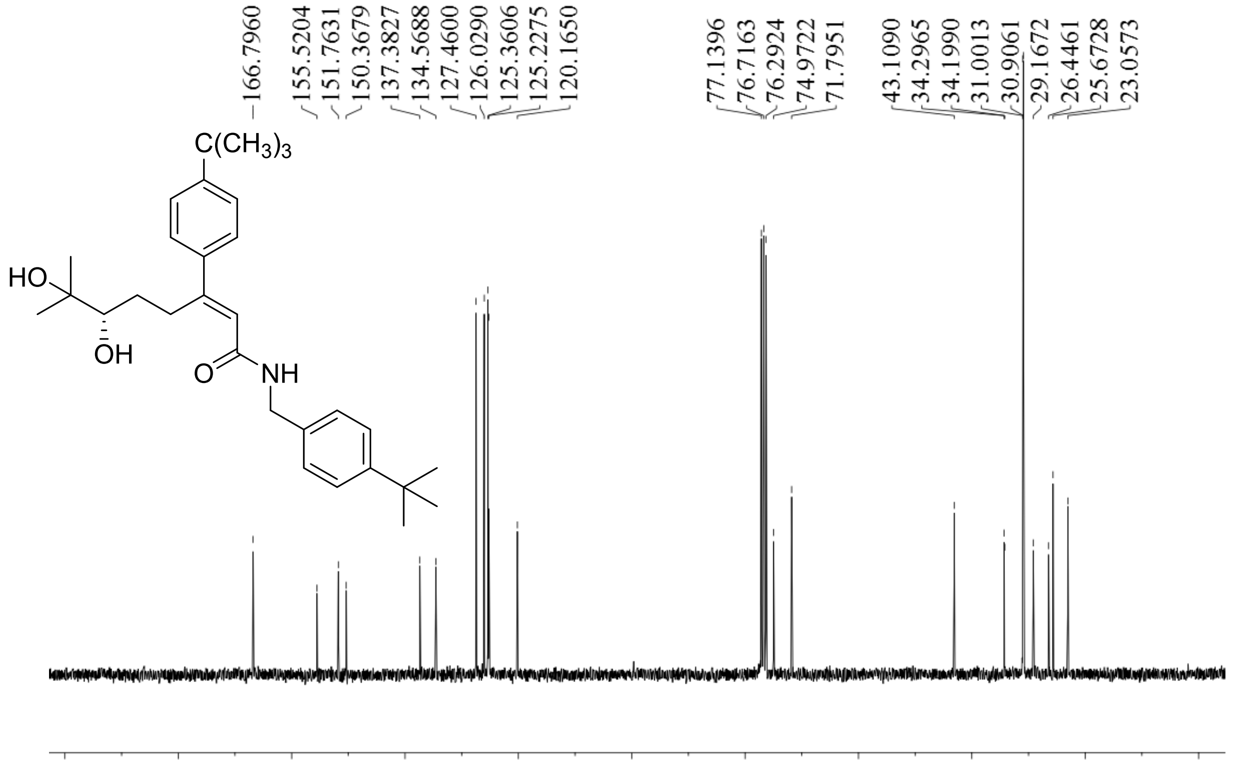

Figure S40. ^{13}C NMR spectrum of (*S*)-**11b**

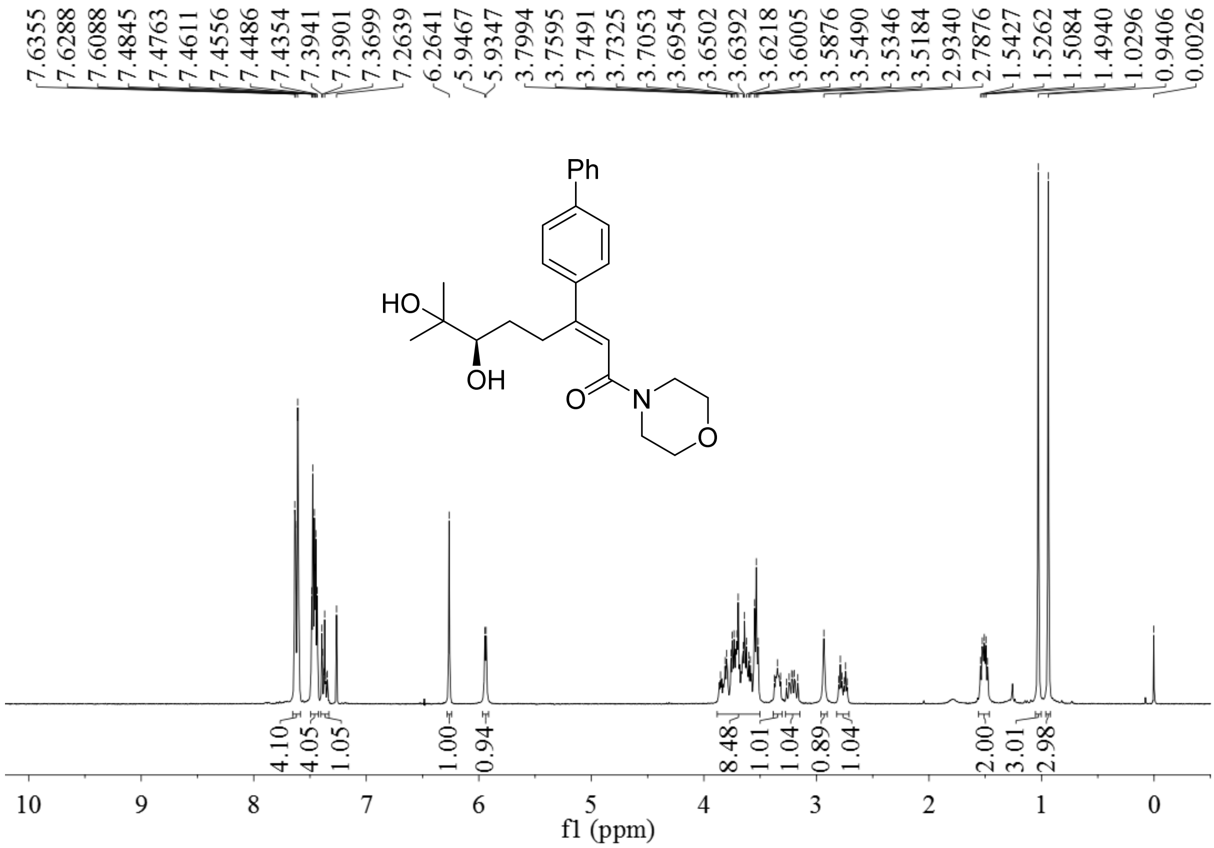

Figure S41. ^{1}H NMR of Compound (*R*)-**11c**

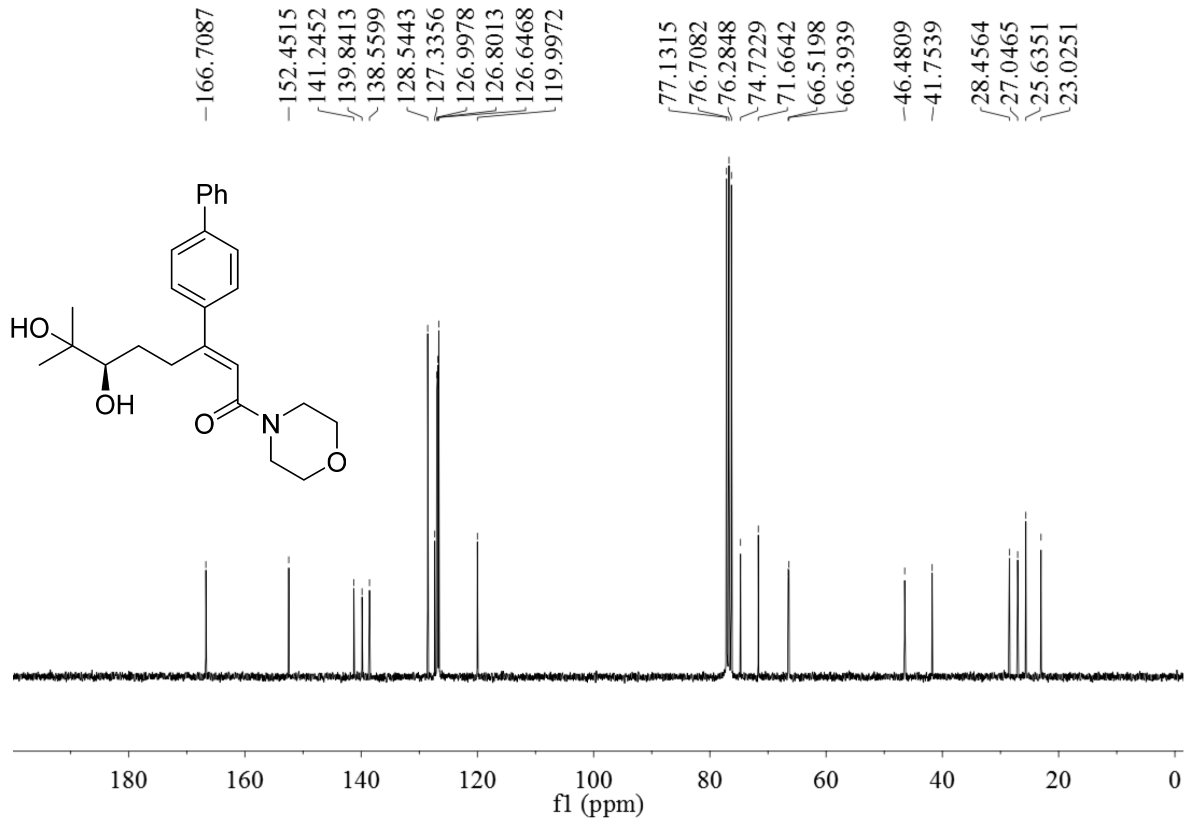

Figure S42. ^{13}C NMR spectrum of (*R*)-**11c**

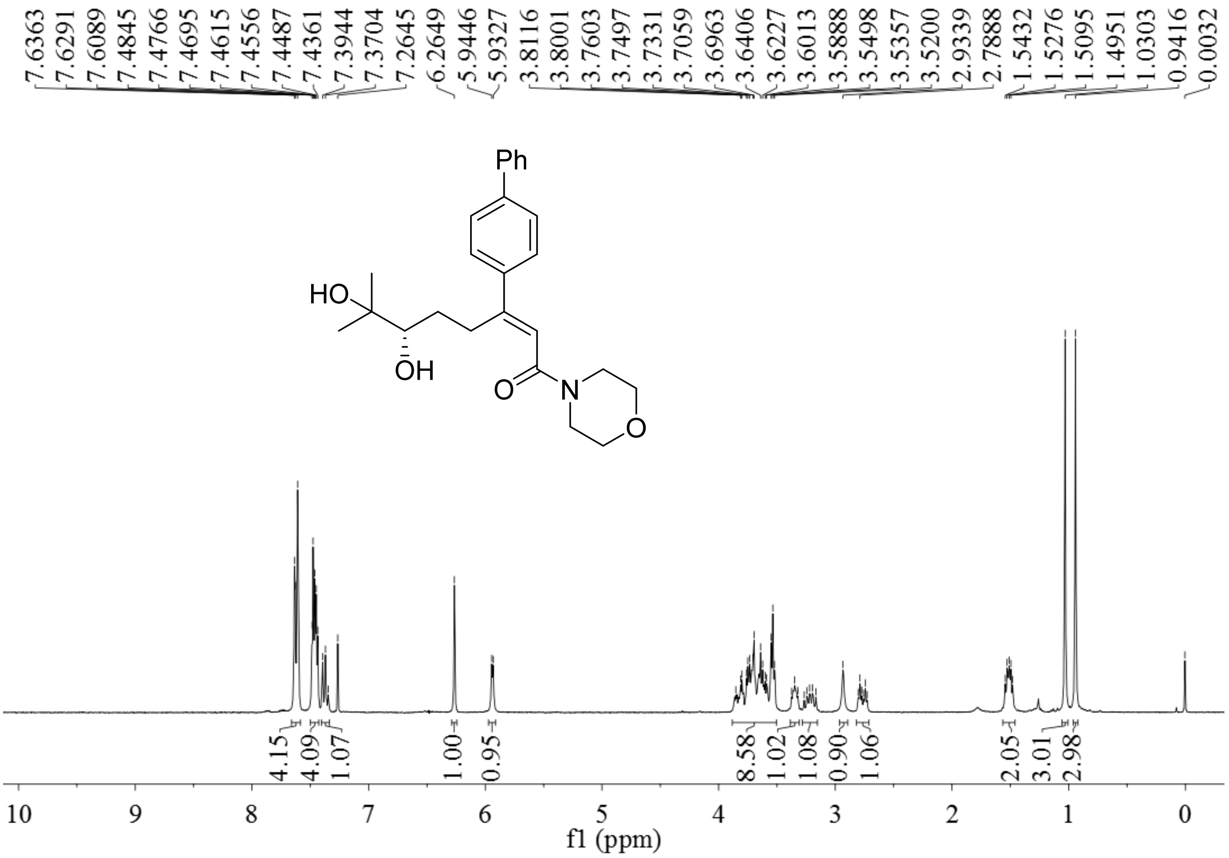

Figure S43. ^{1}H NMR of Compound (*S*)-**11c**

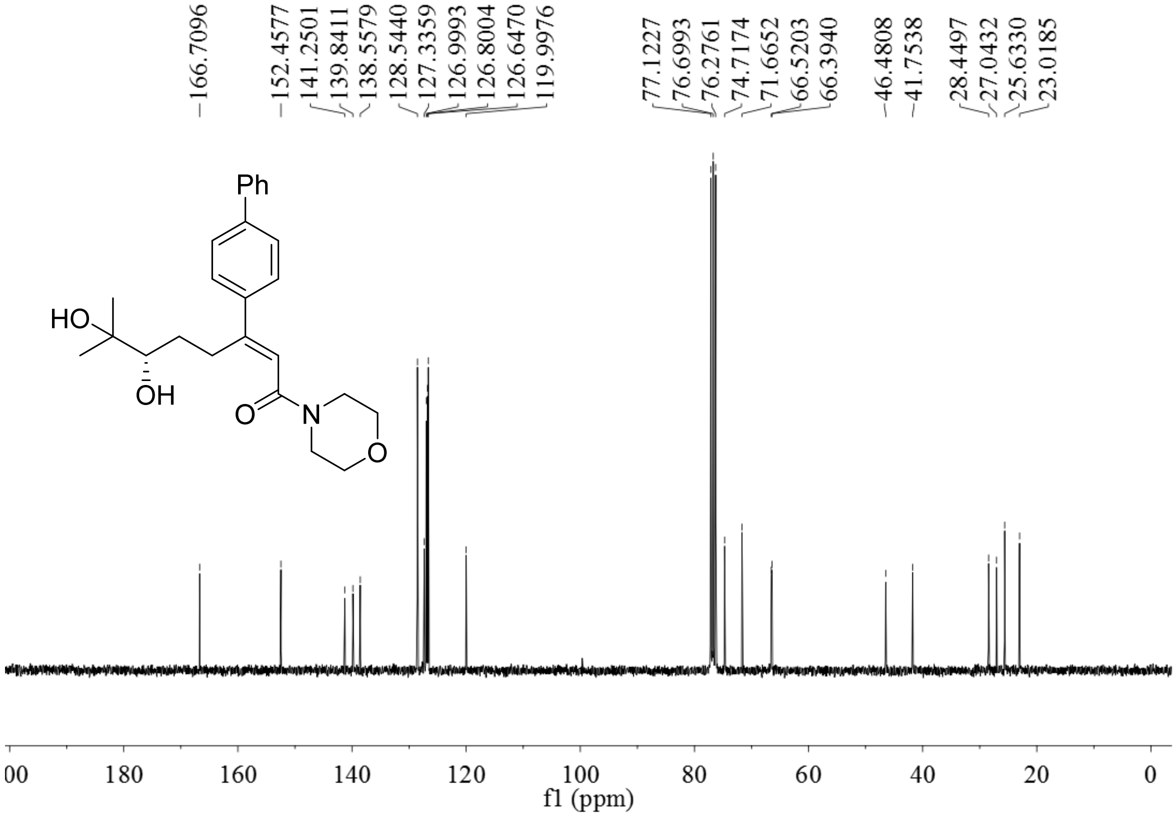

Figure S44. ^{13}C NMR spectrum of (*S*)-**11c**

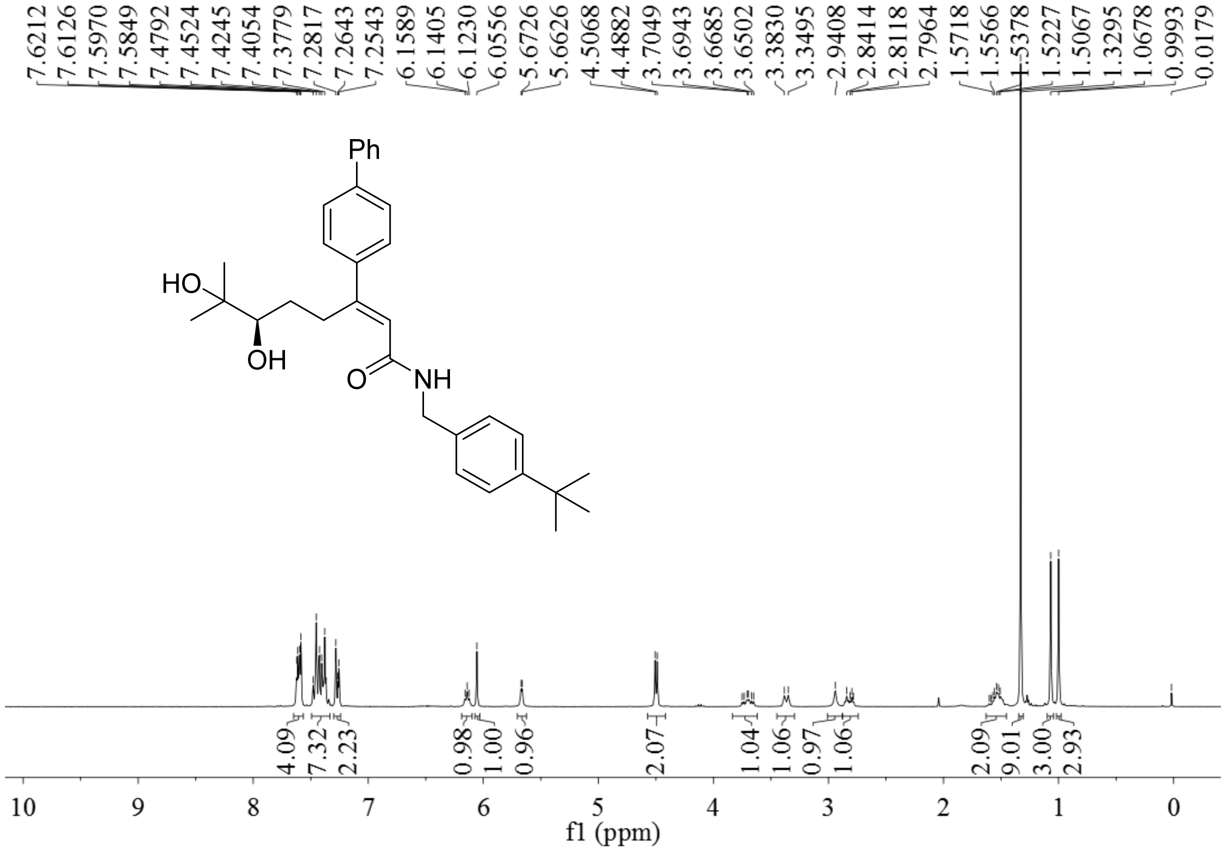

Figure S45. ^{1}H NMR of Compound (*R*)-**11d**

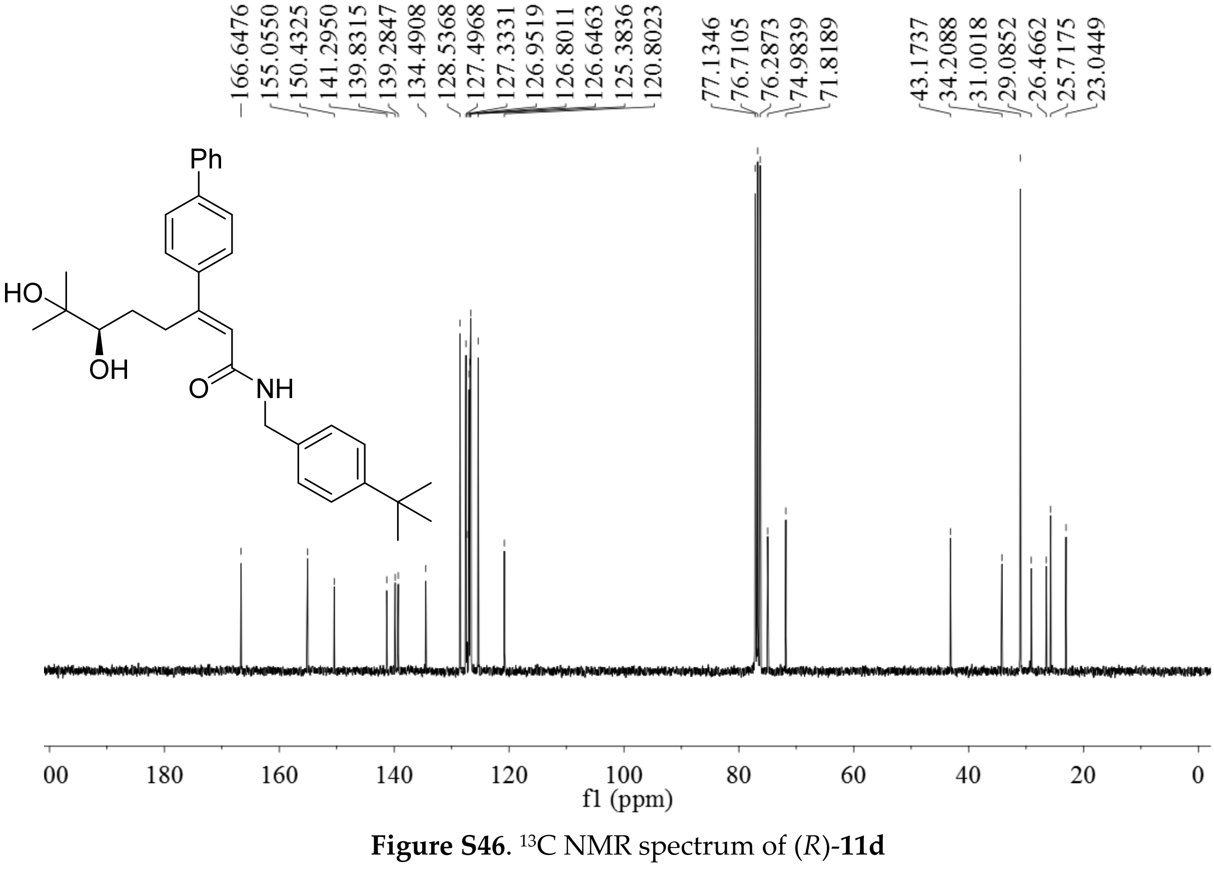

Figure S46. ^{13}C NMR spectrum of (*R*)-**11d**

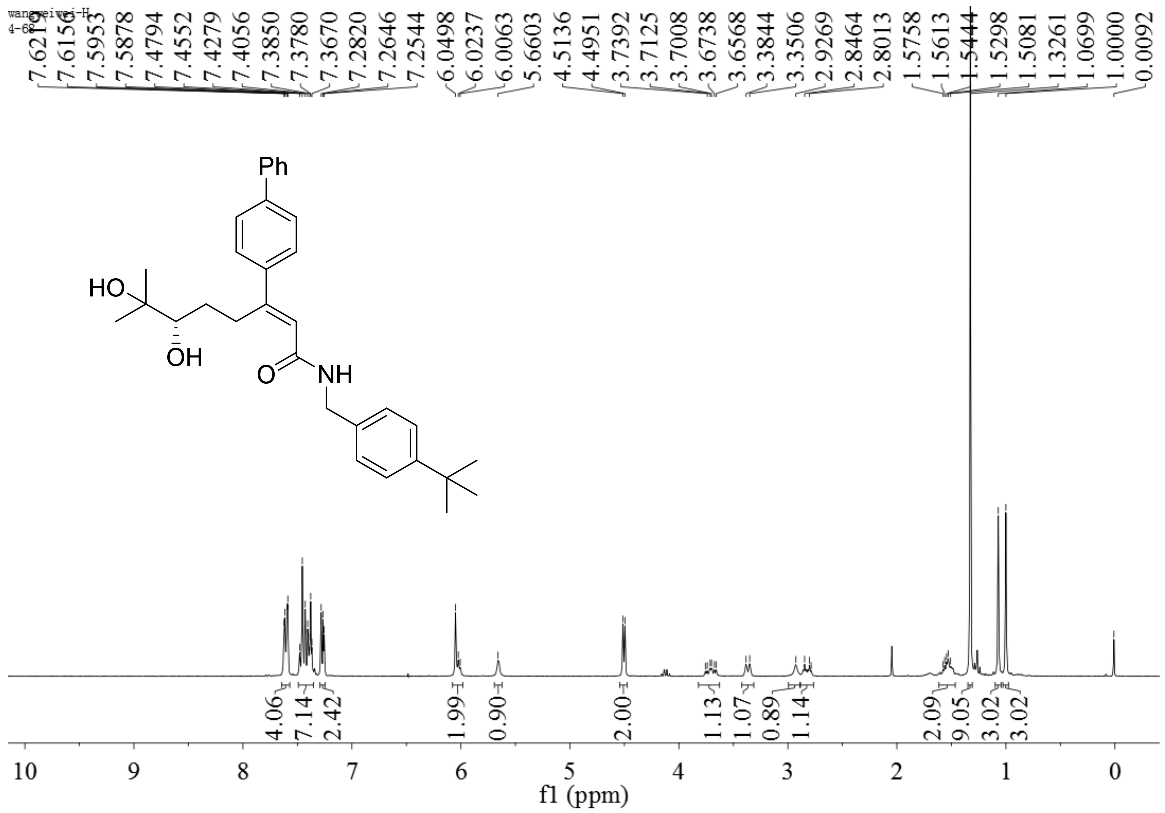

Figure S47. ^{1}H NMR of Compound (*S*)-**11d**

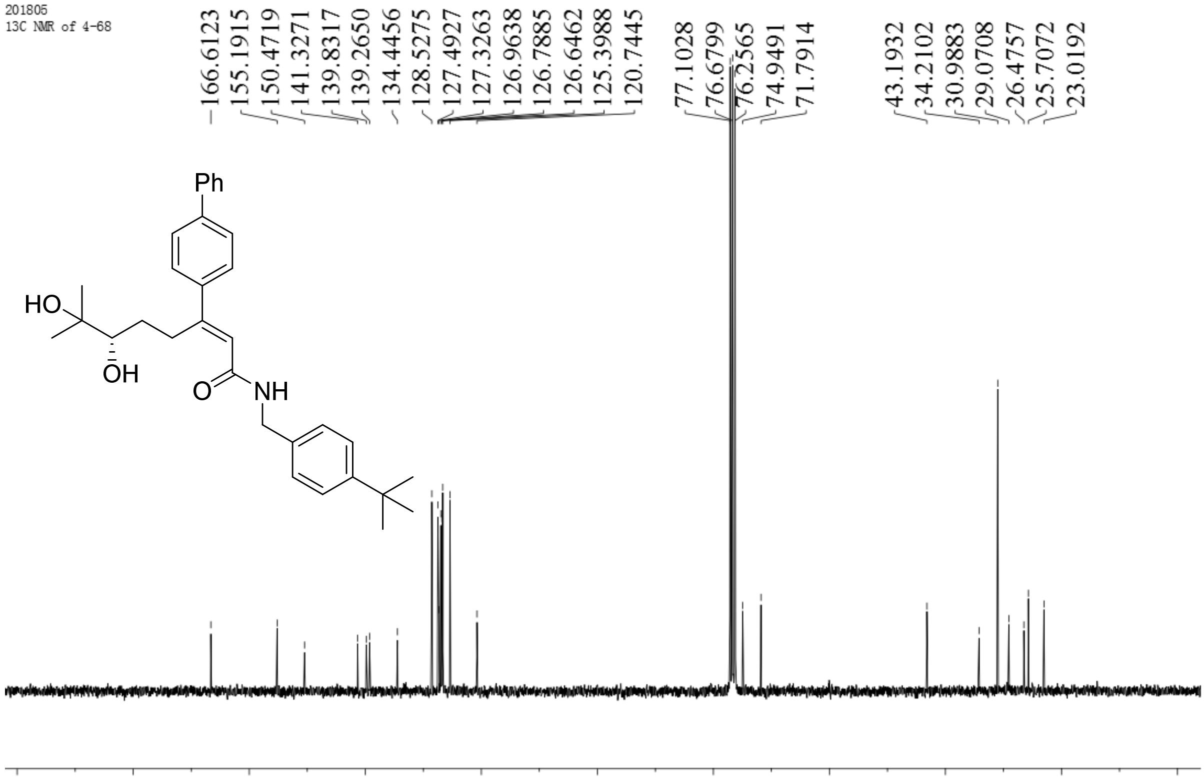

Figure S48. ^{13}C NMR spectrum of (*S*)-**11d**

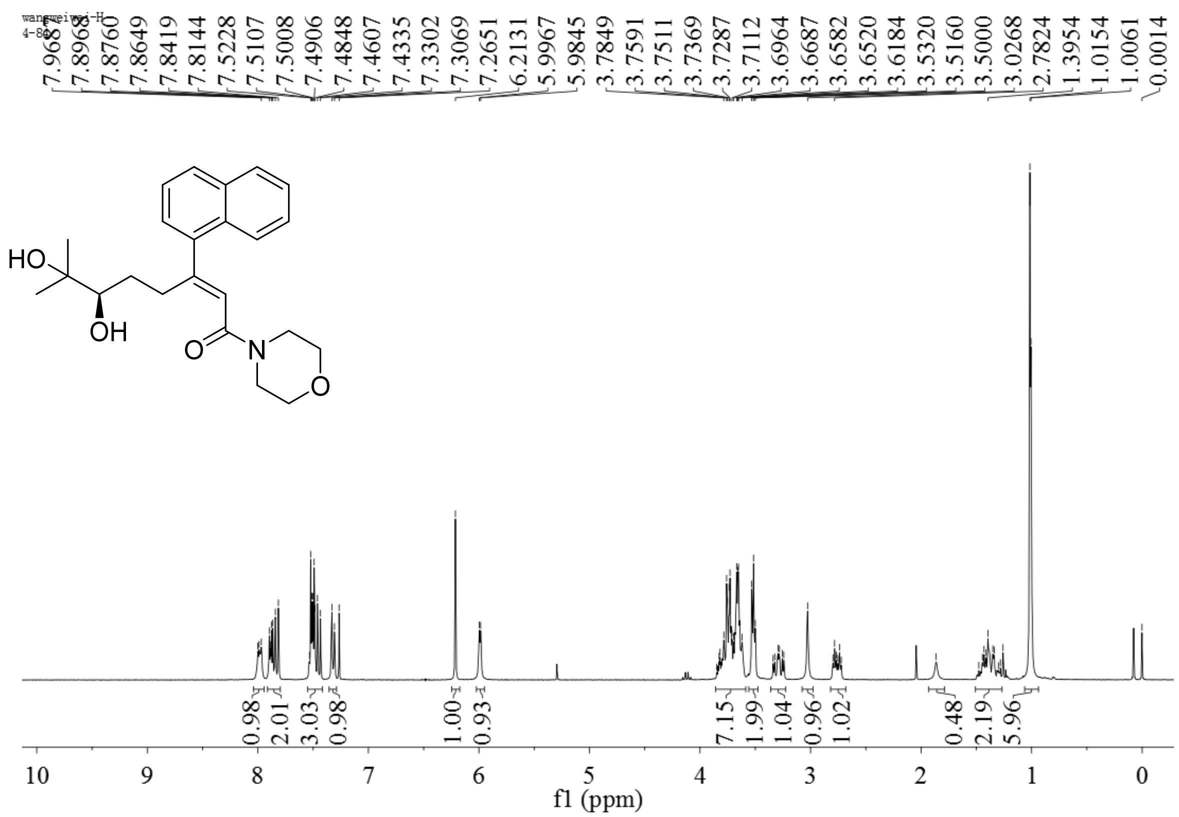

Figure S49. ^{1}H NMR of Compound (*R*)-**11e**

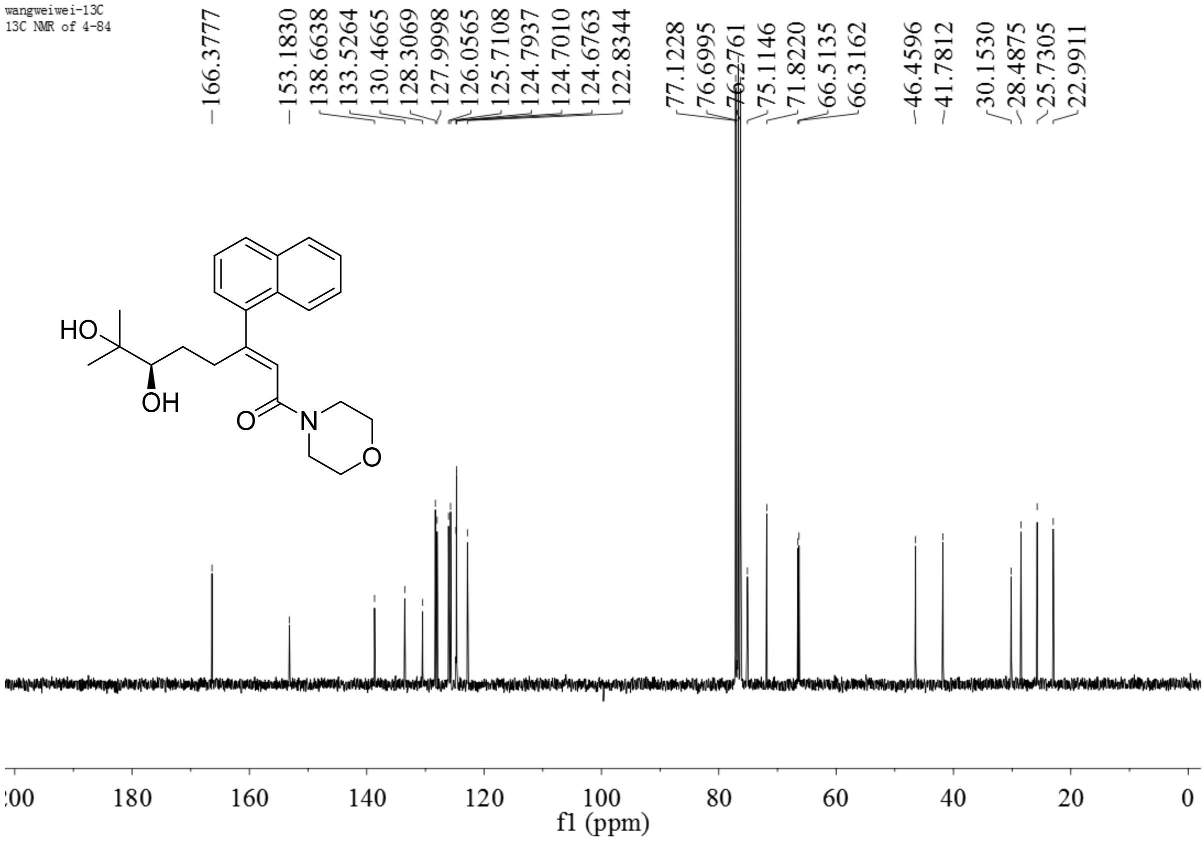

Figure S50. ^{13}C NMR spectrum of (*R*)-**11e**

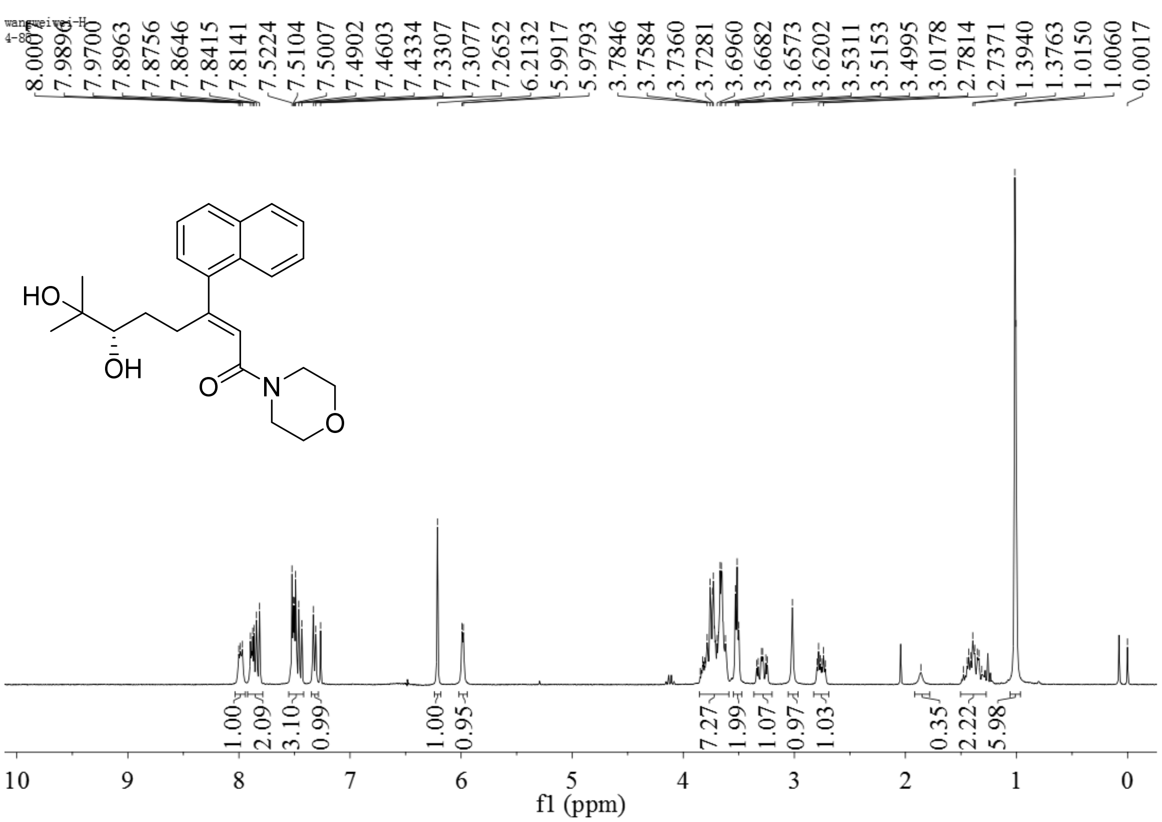

Figure S51. ¹H NMR of Compound (*S*)-**11e**

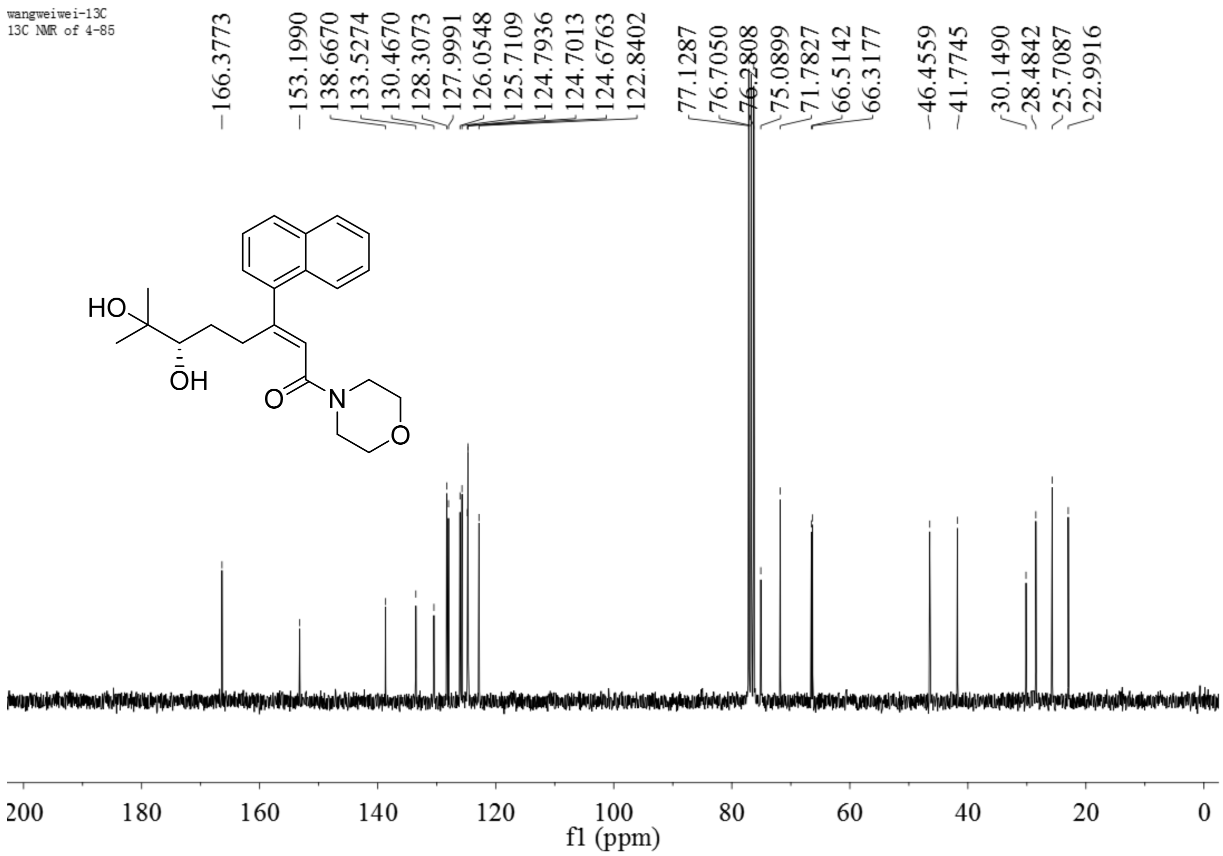

Figure S52. ¹³C NMR spectrum of (*S*)-**11e**

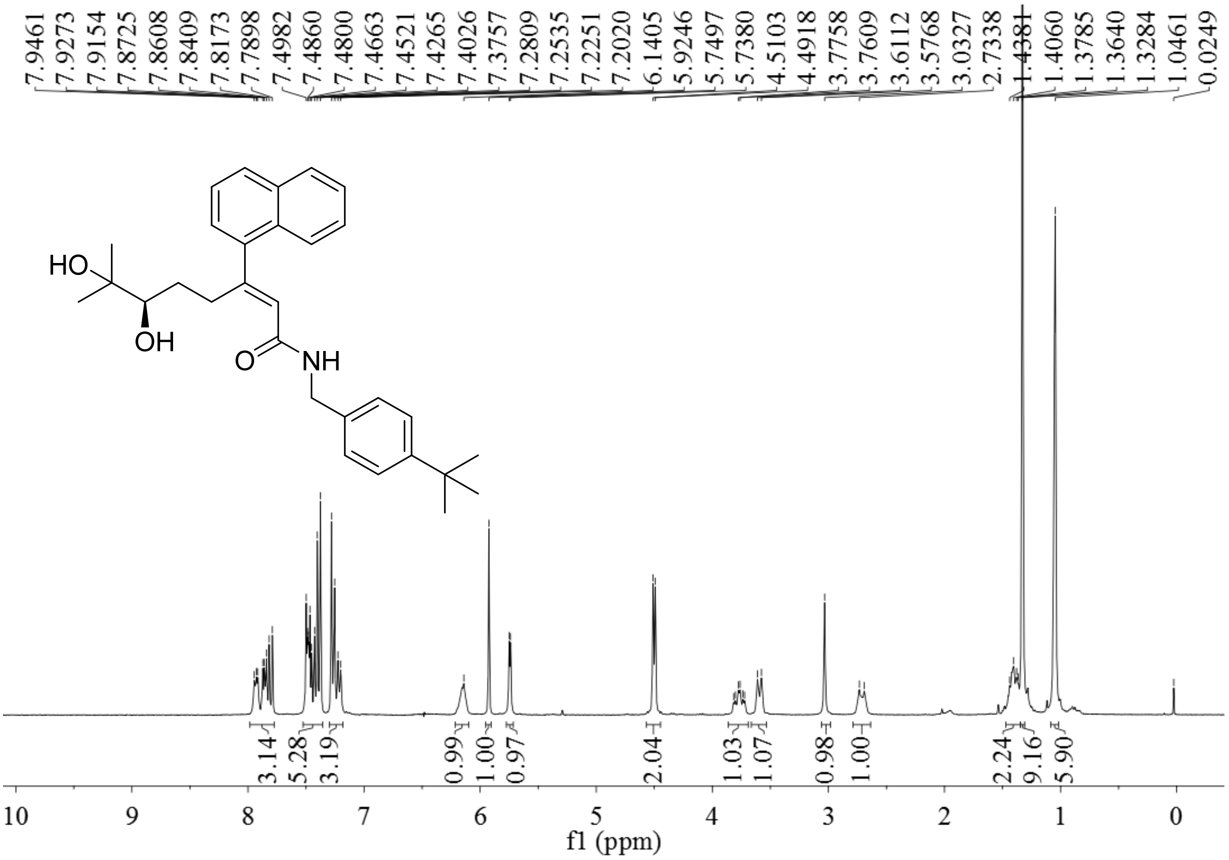

Figure S53. ^{1}H NMR of Compound (*R*)-**11f**

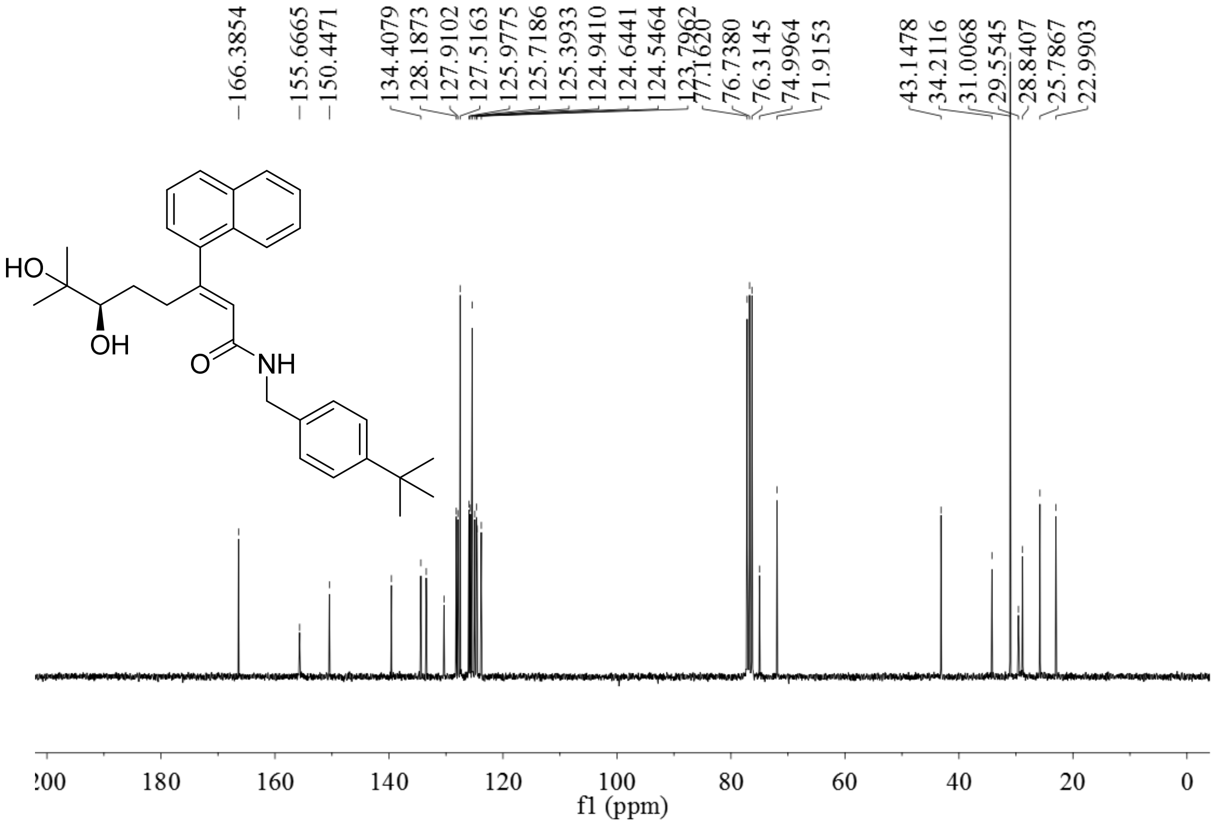

Figure S54. ^{13}C NMR spectrum of (*R*)-**11f**

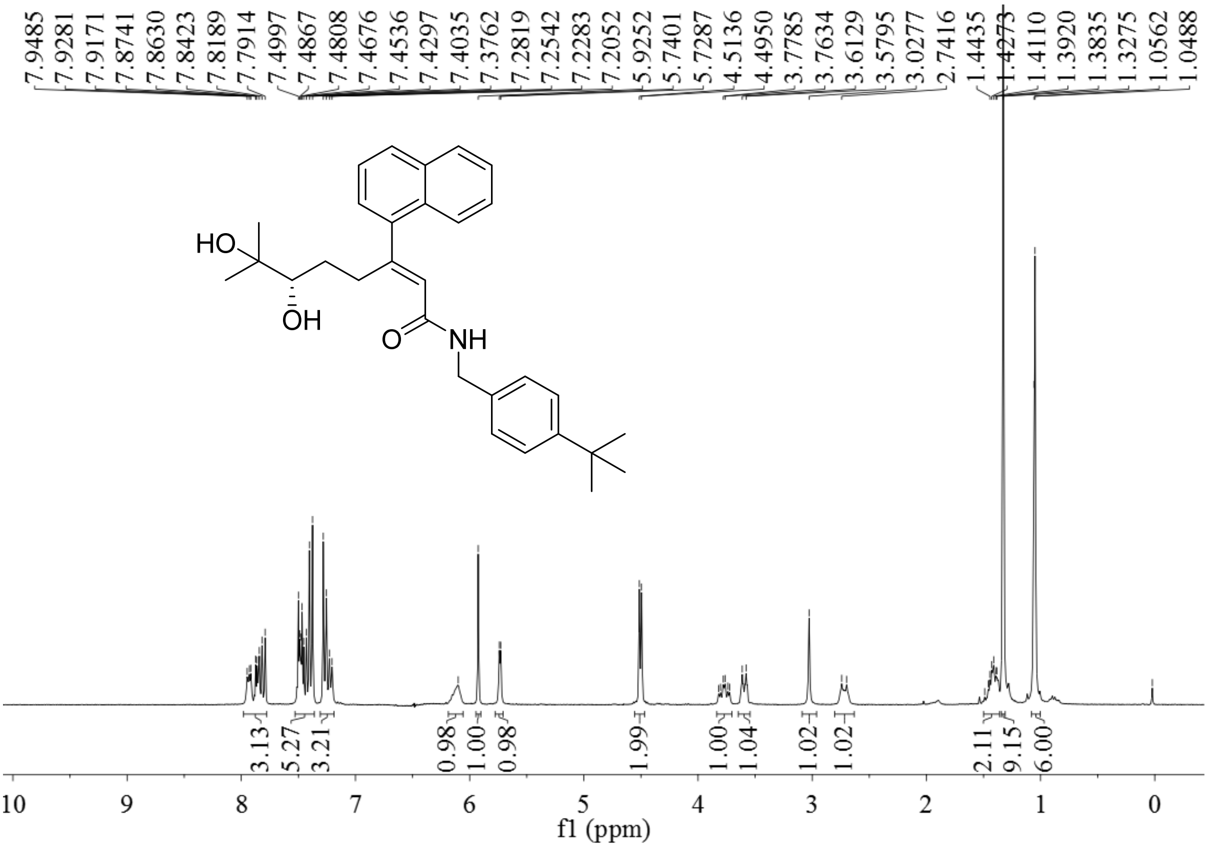

Figure S55. ^{1}H NMR of Compound (*S*)-**11f**

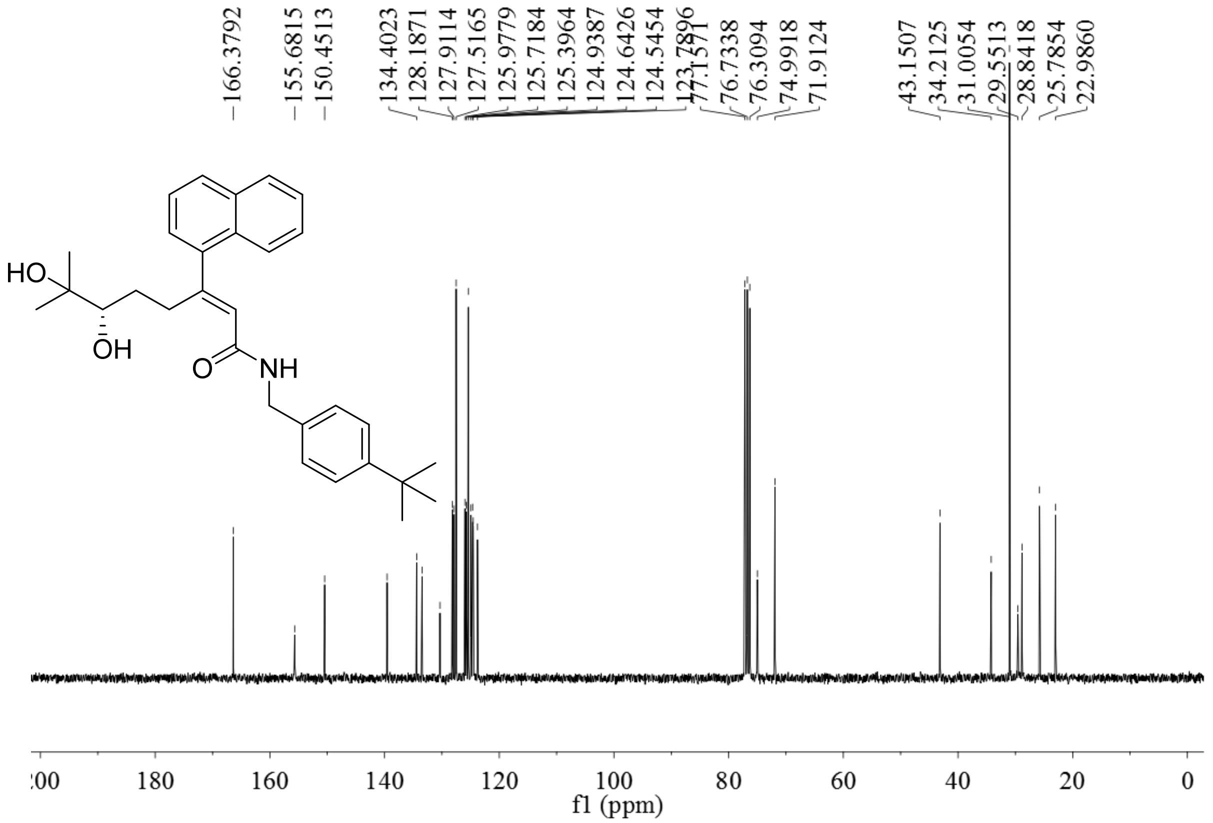

Figure S56. ^{13}C NMR spectrum of (*S*)-**11f**

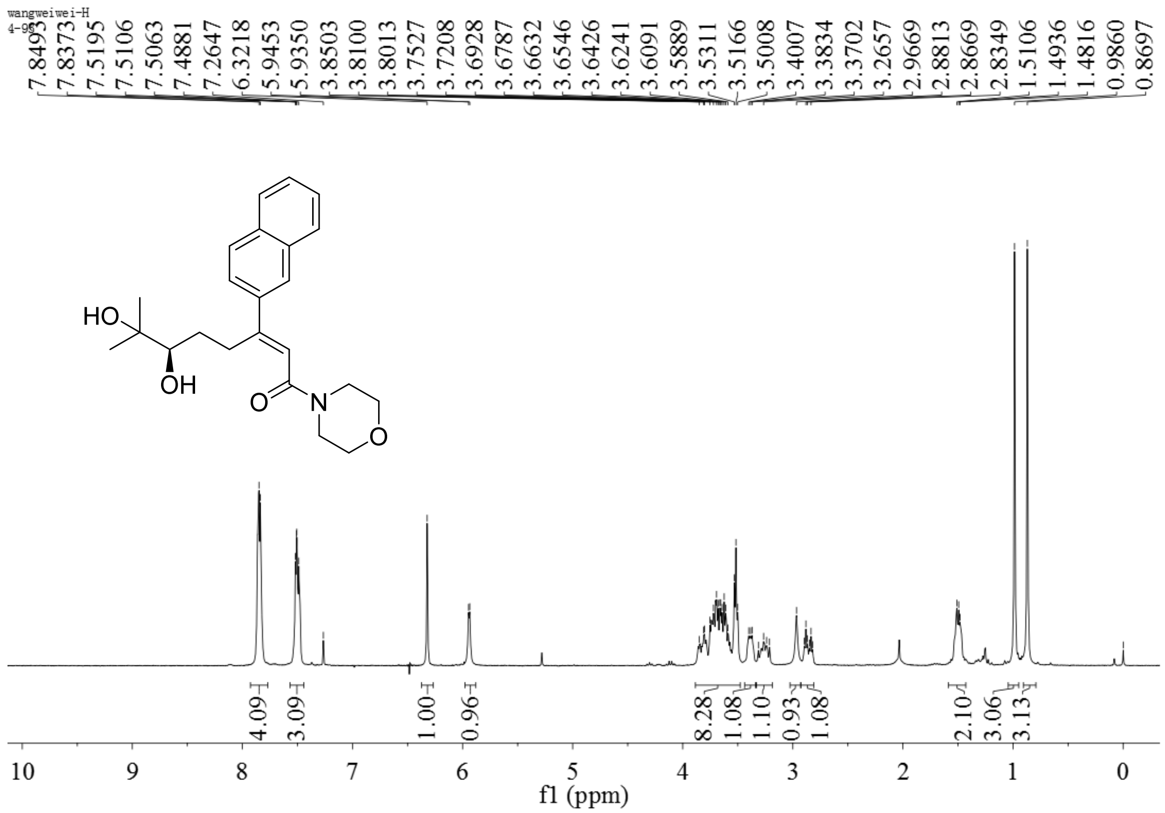

Figure S57. ^{1}H NMR of Compound (*R*)-**11g**

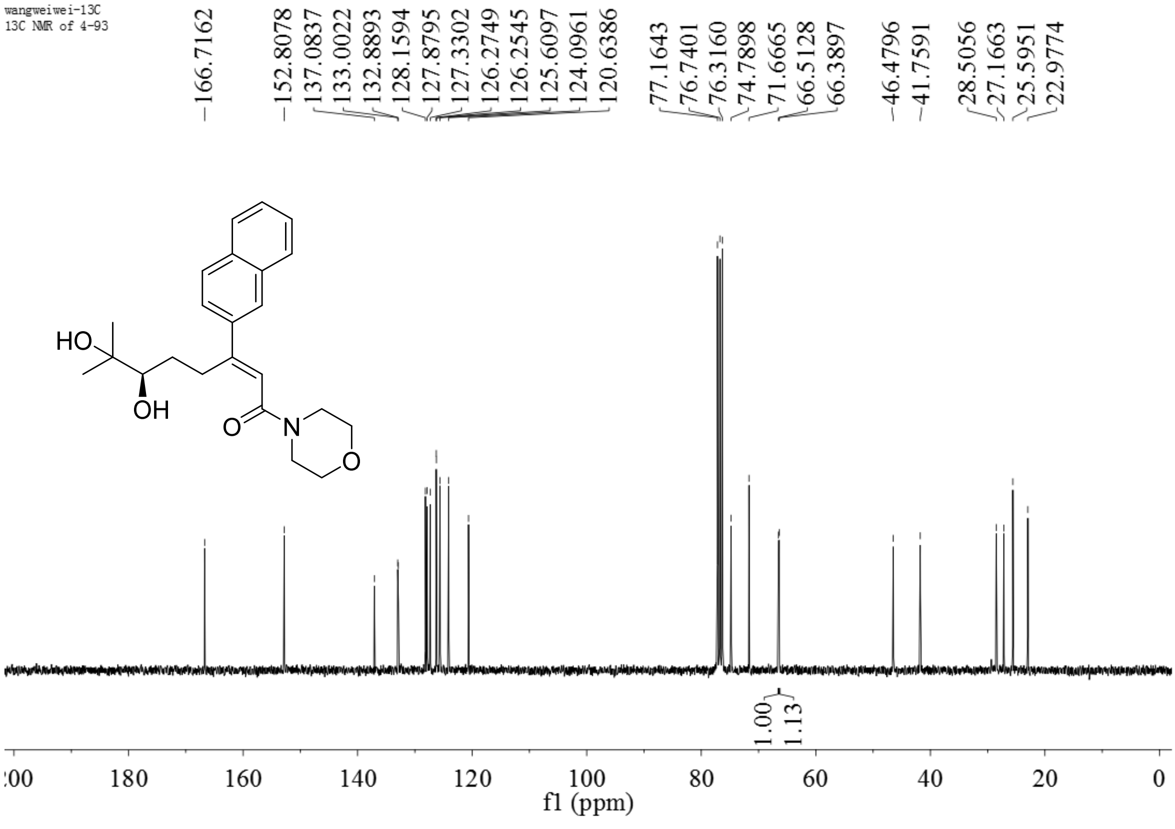

Figure S58. ^{13}C NMR spectrum of (*R*)-**11g**

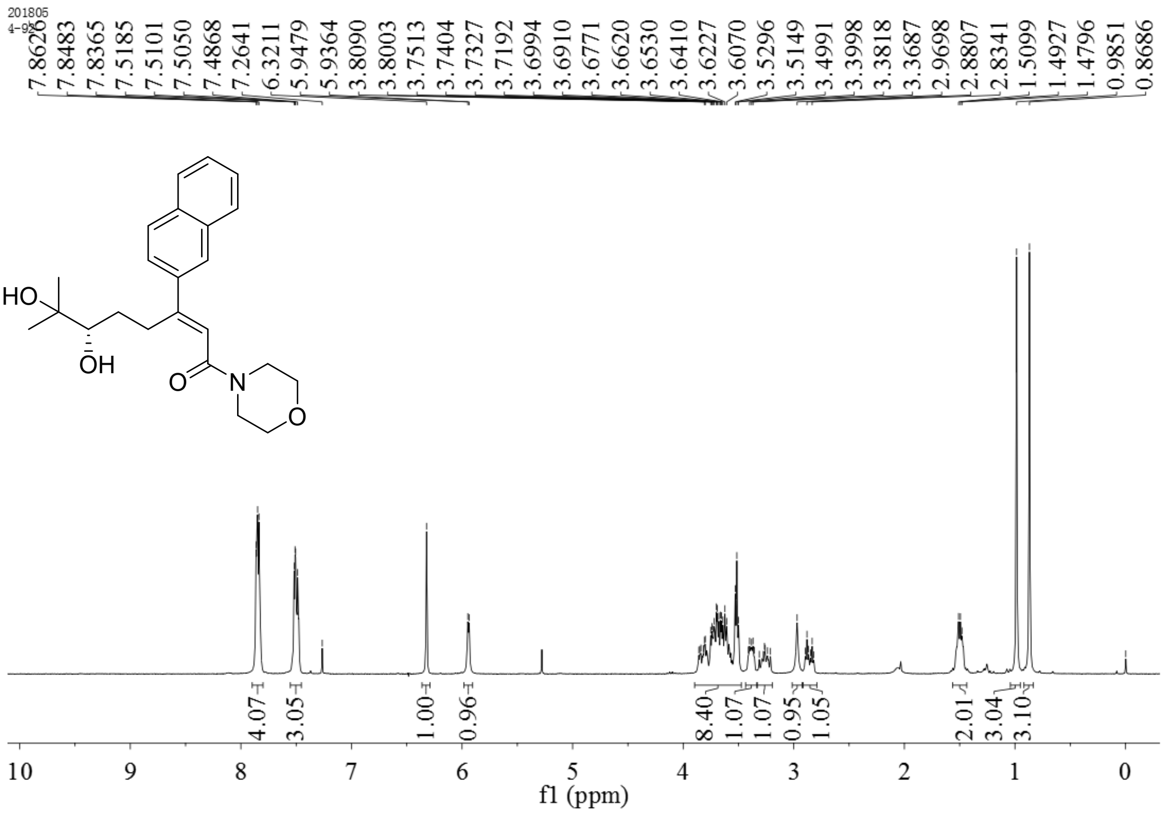

Figure S59. ^{1}H NMR of Compound (*S*)-**11g**

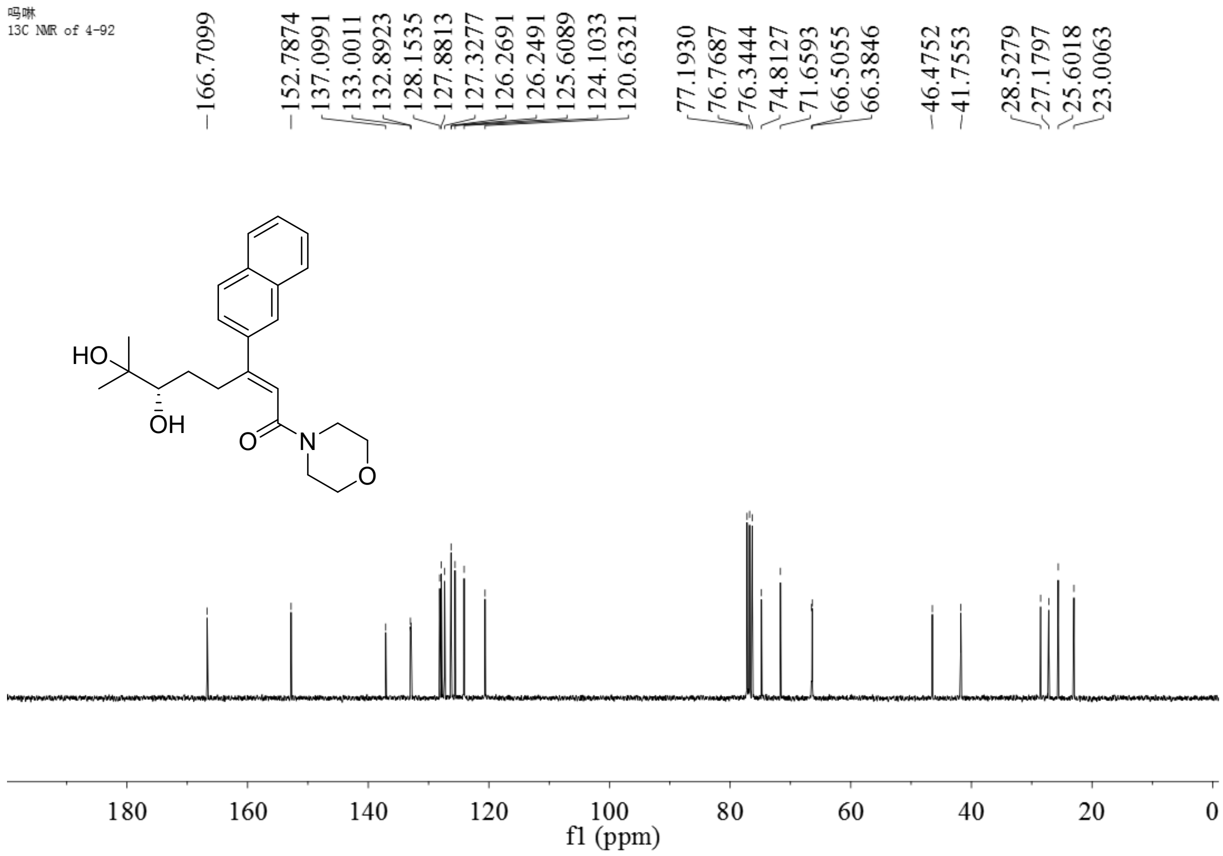

Figure S60. ^{13}C NMR spectrum of (*S*)-**11g**

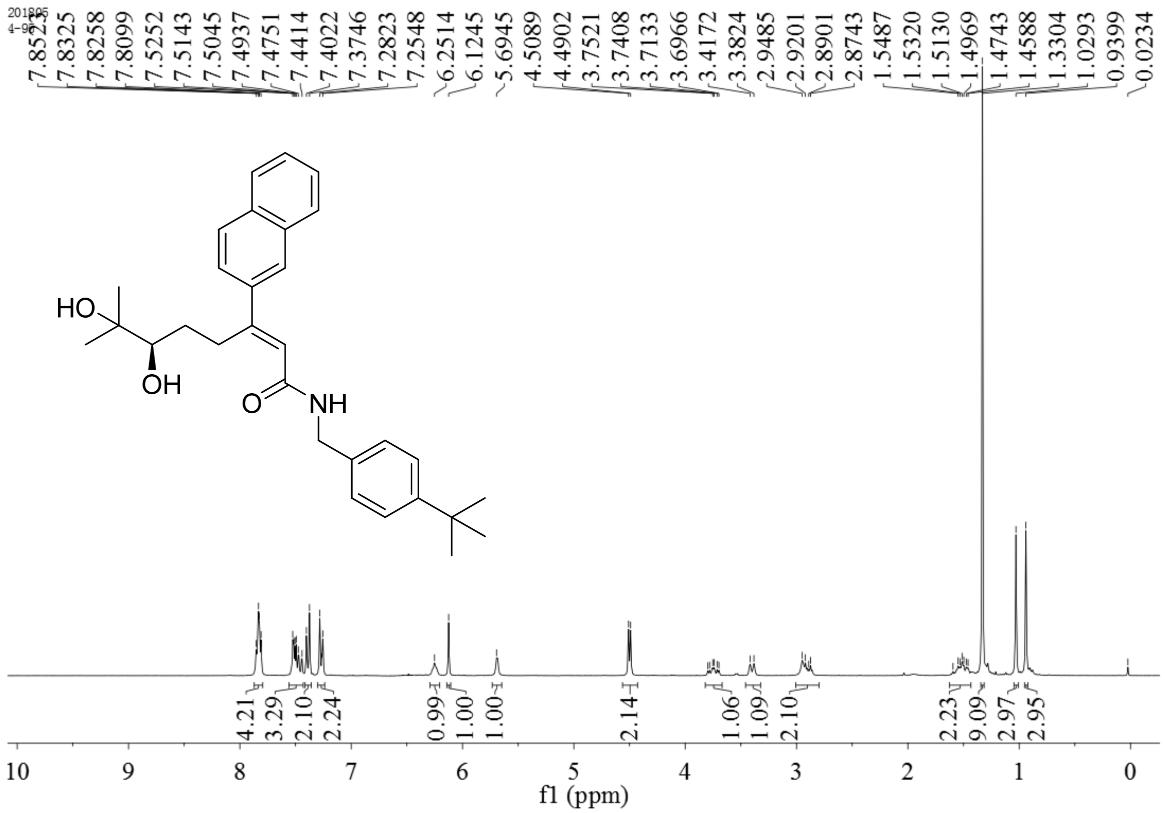

Figure S61. ^{1}H NMR of Compound (*R*)-**11h**

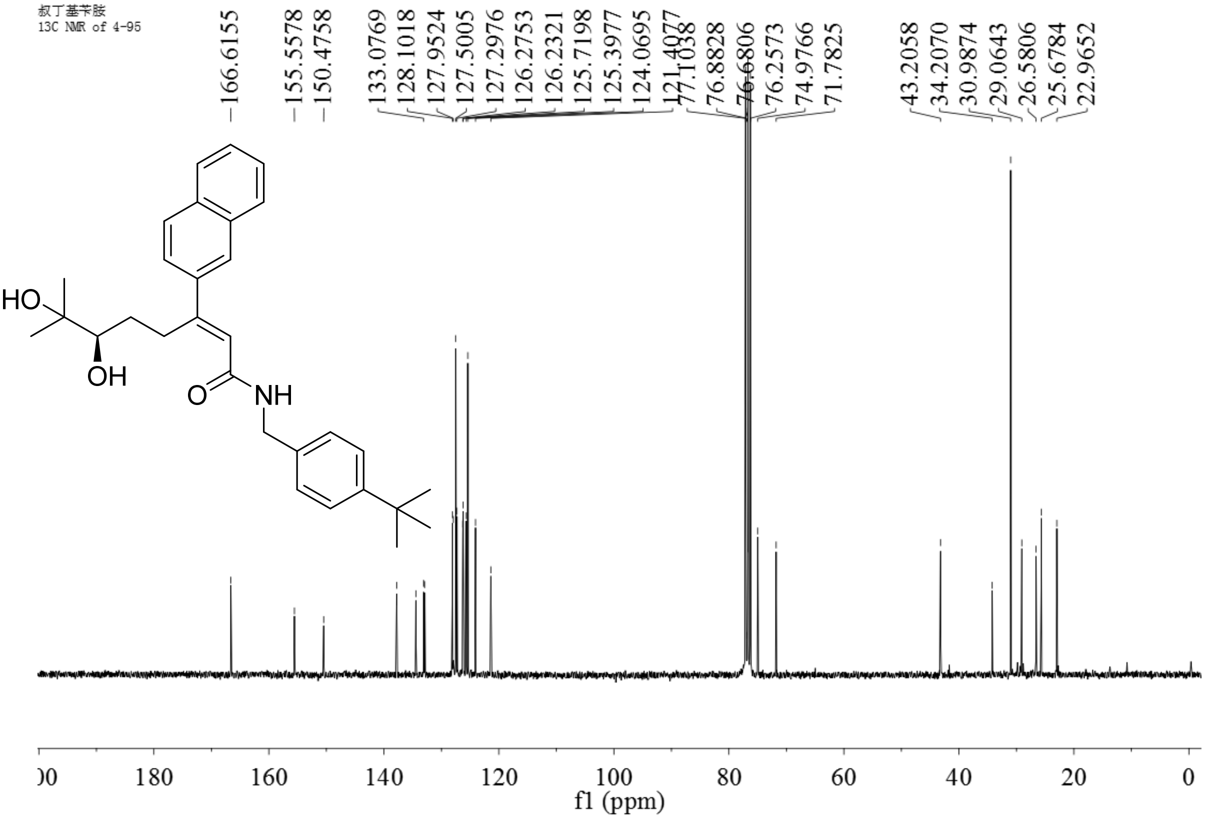

Figure S62. ^{13}C NMR spectrum of (*R*)-**11h**

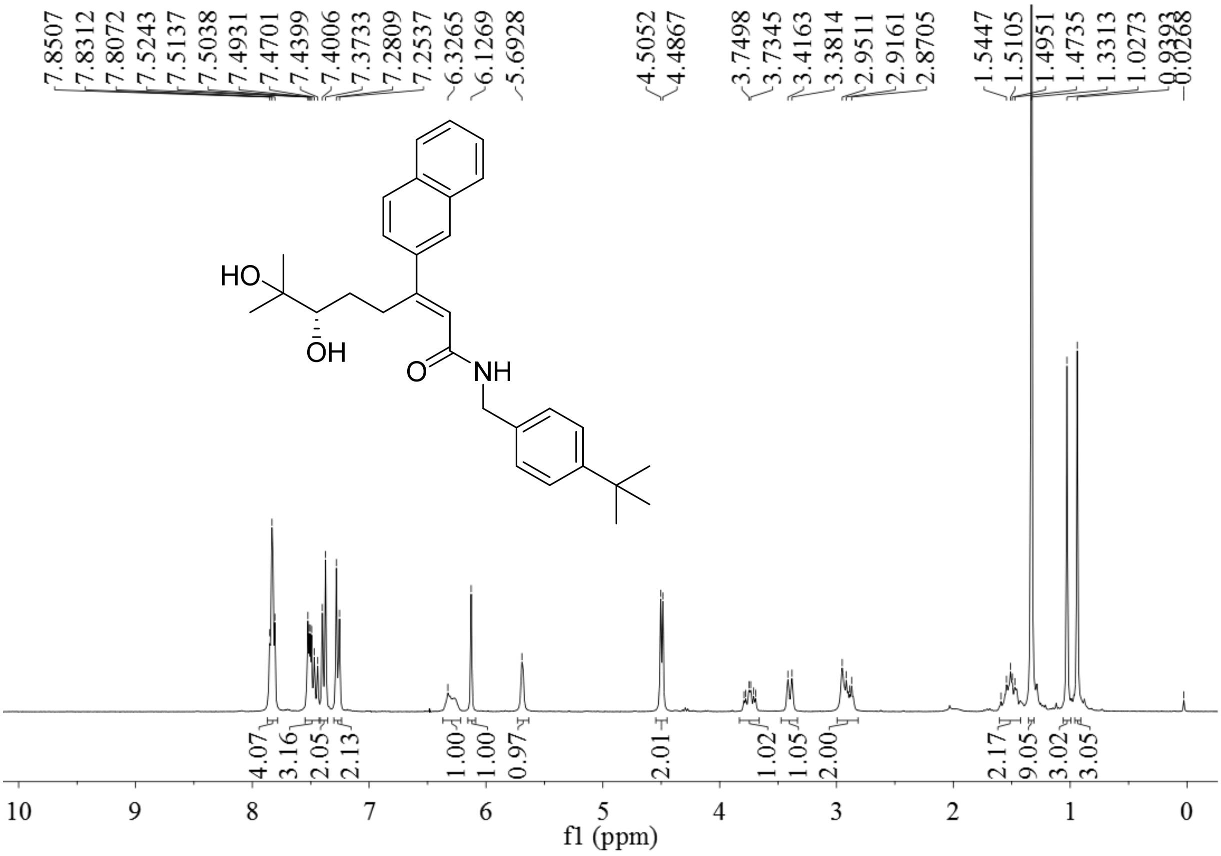

Figure S63. ^{1}H NMR of Compound (*S*)-**11h**

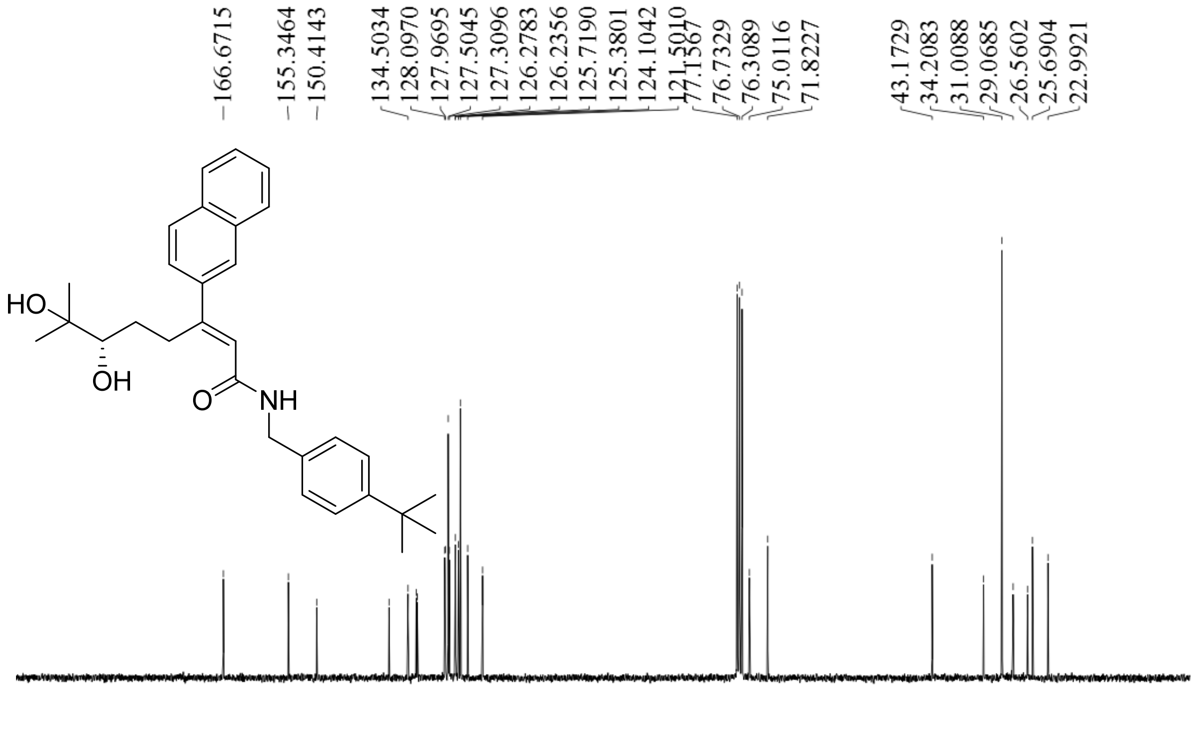

Figure S64. ^{13}C NMR spectrum of (*S*)-**11h**

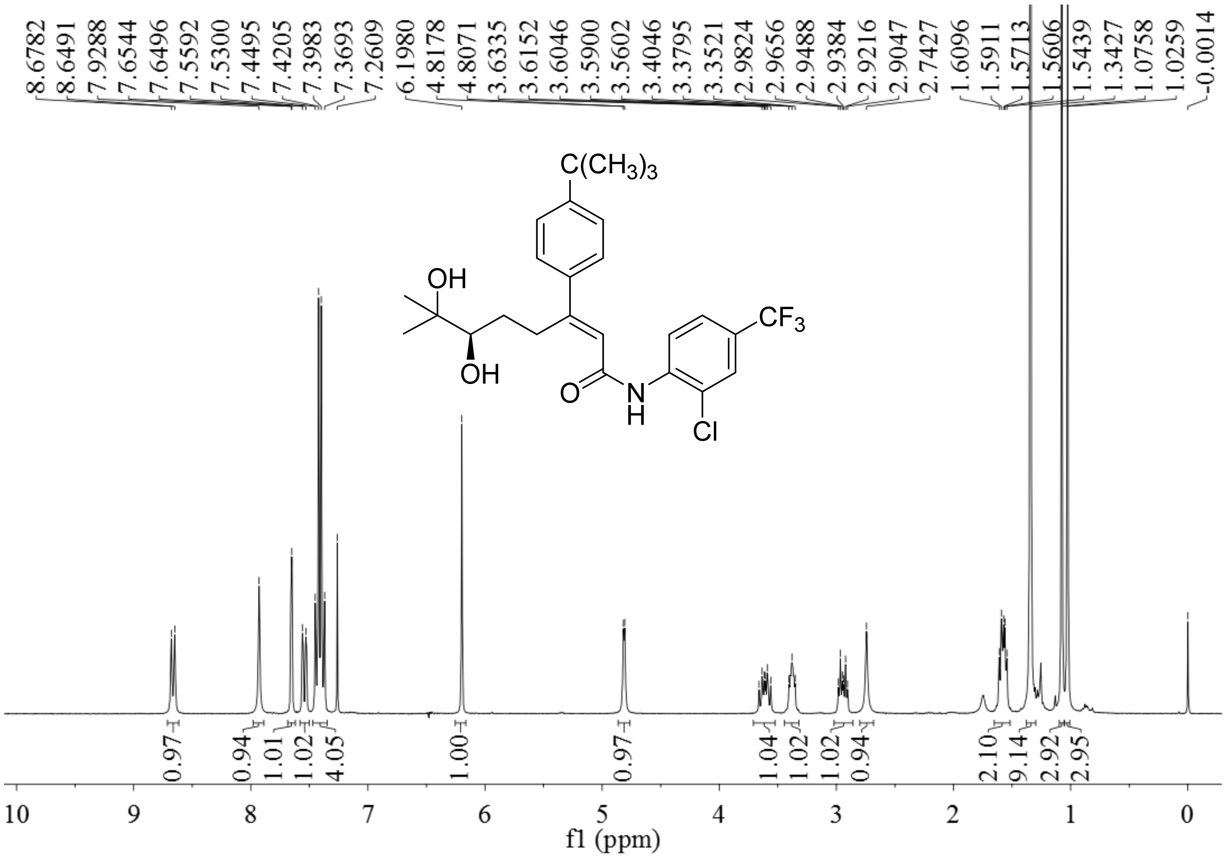

Figure S65. ^{1}H NMR of Compound (*R*)-**11i**

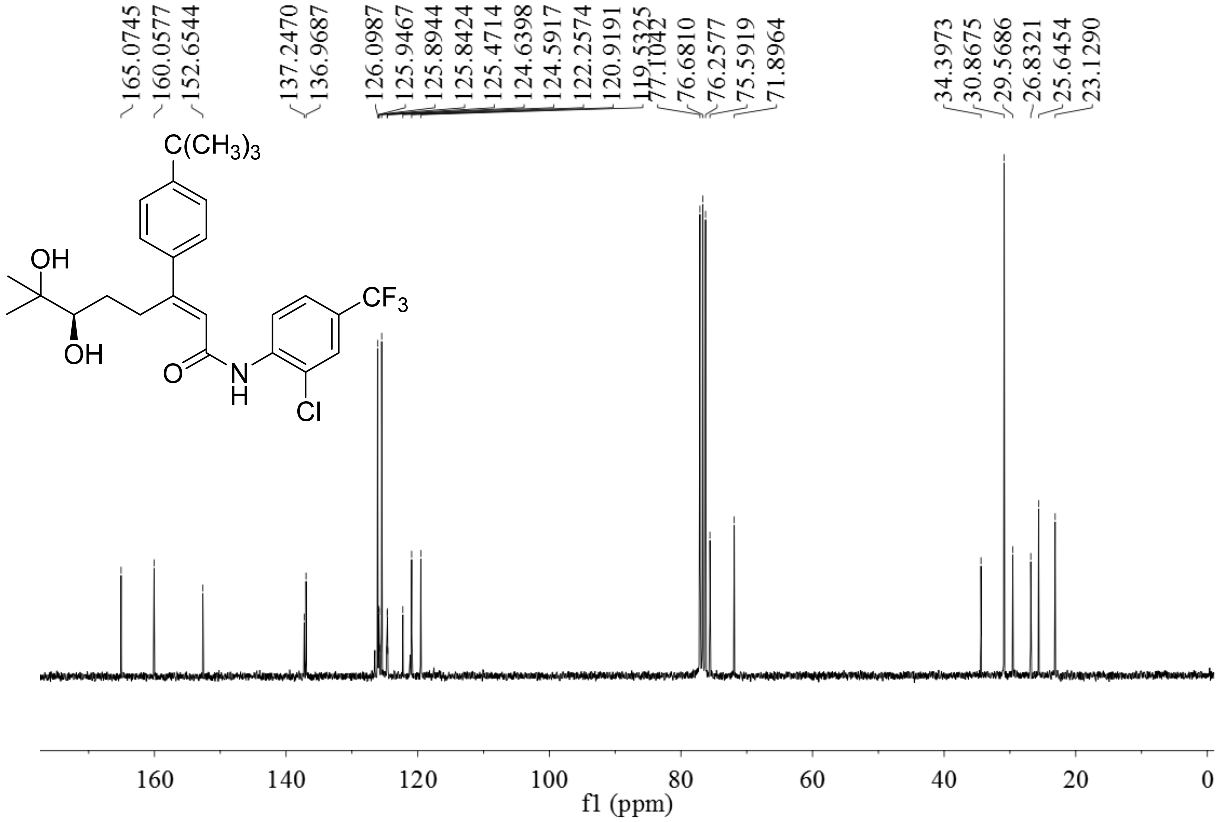

Figure S66. ^{13}C NMR spectrum of (*R*)-**11i**

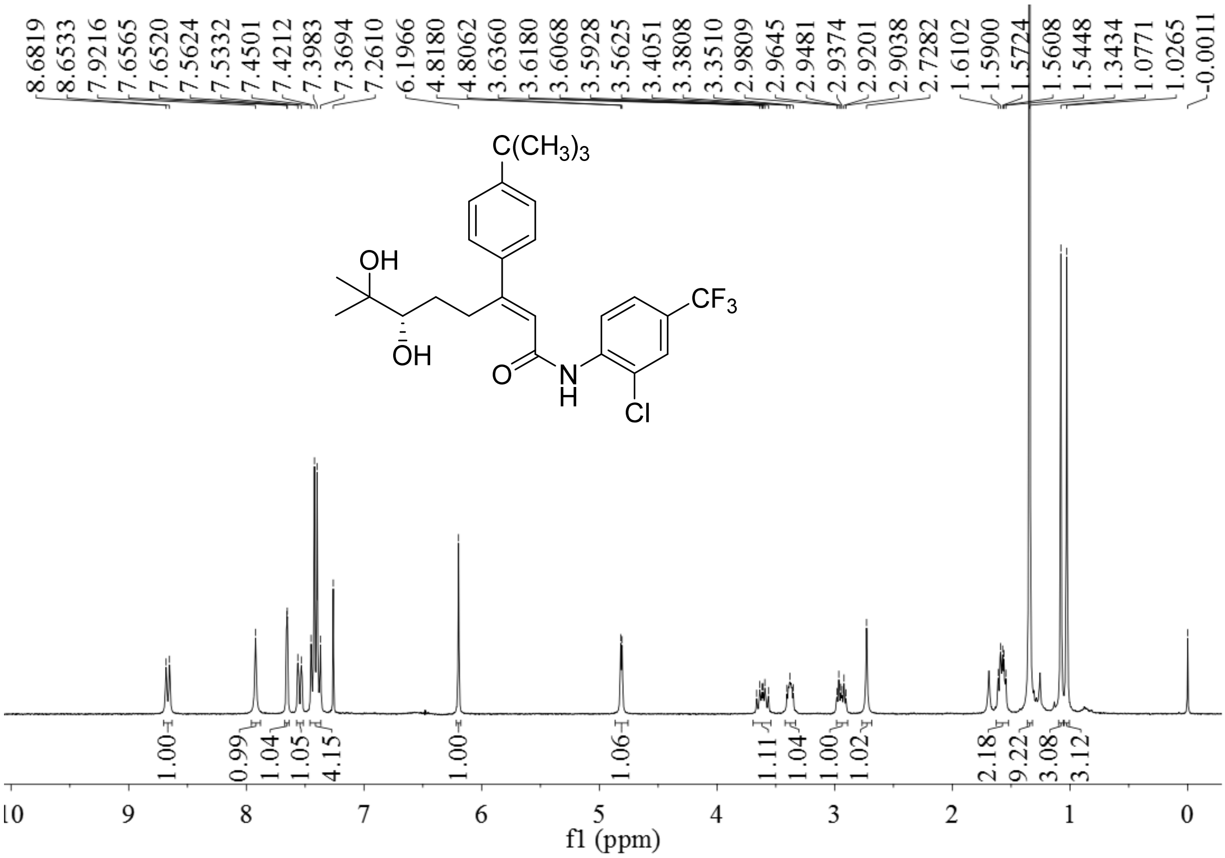

Figure S67. ^{1}H NMR of Compound (*S*)-**11i**

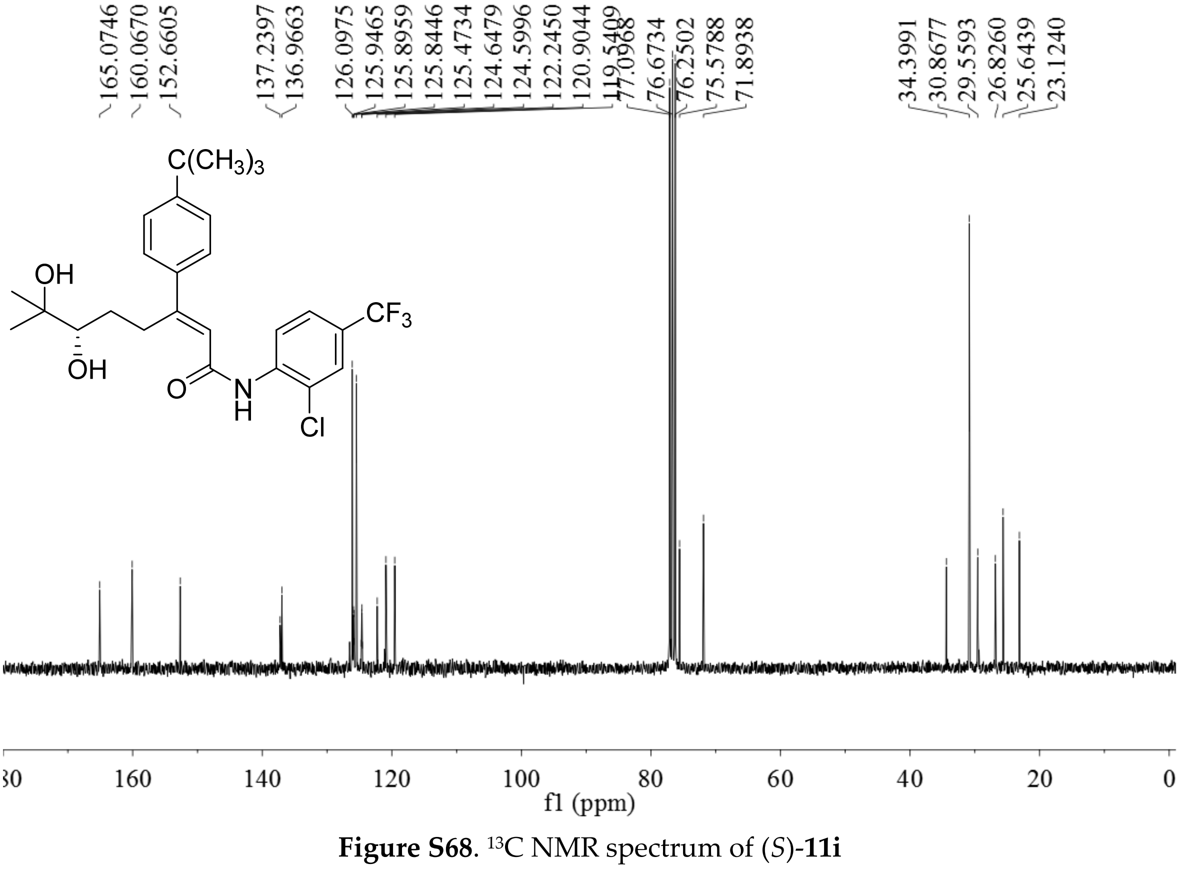

Figure S68. ^{13}C NMR spectrum of (*S*)-**11i**

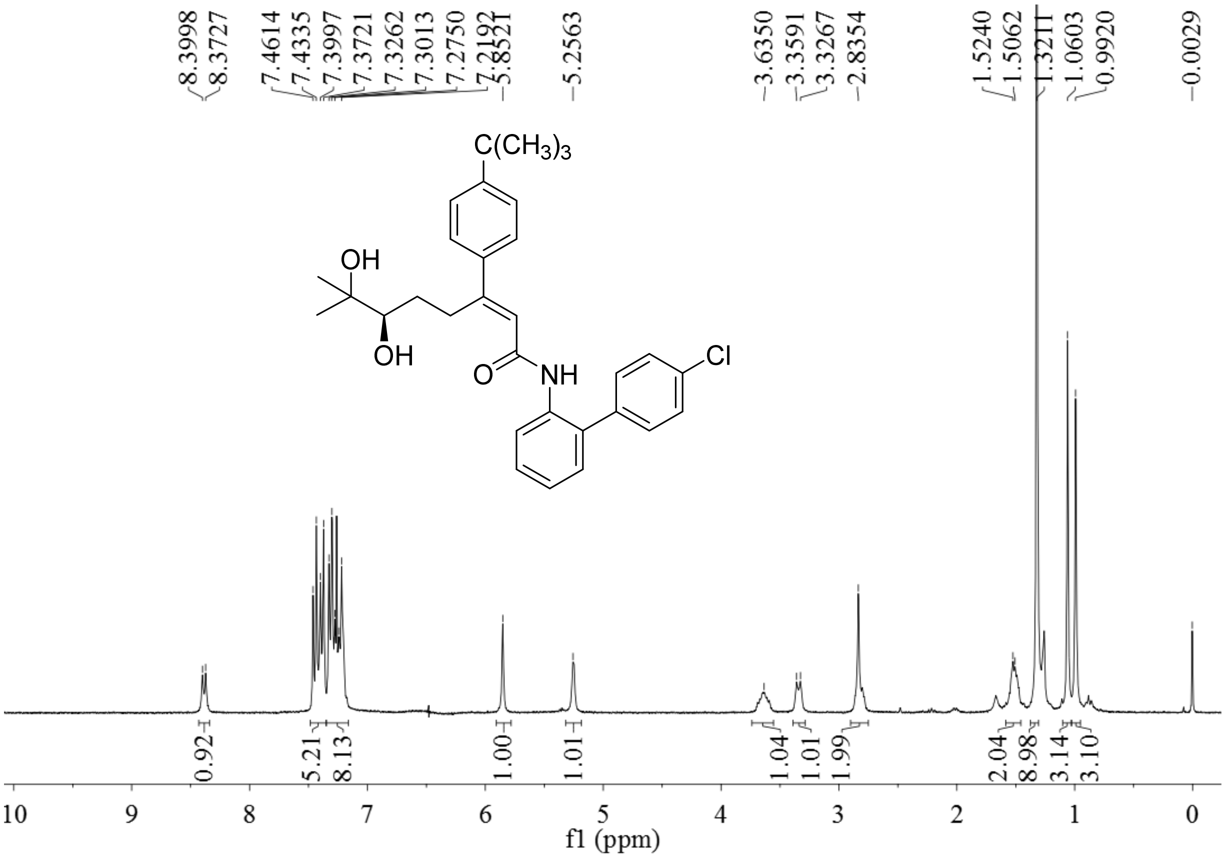

Figure S69. ^{1}H NMR of Compound (*R*)-**11j**

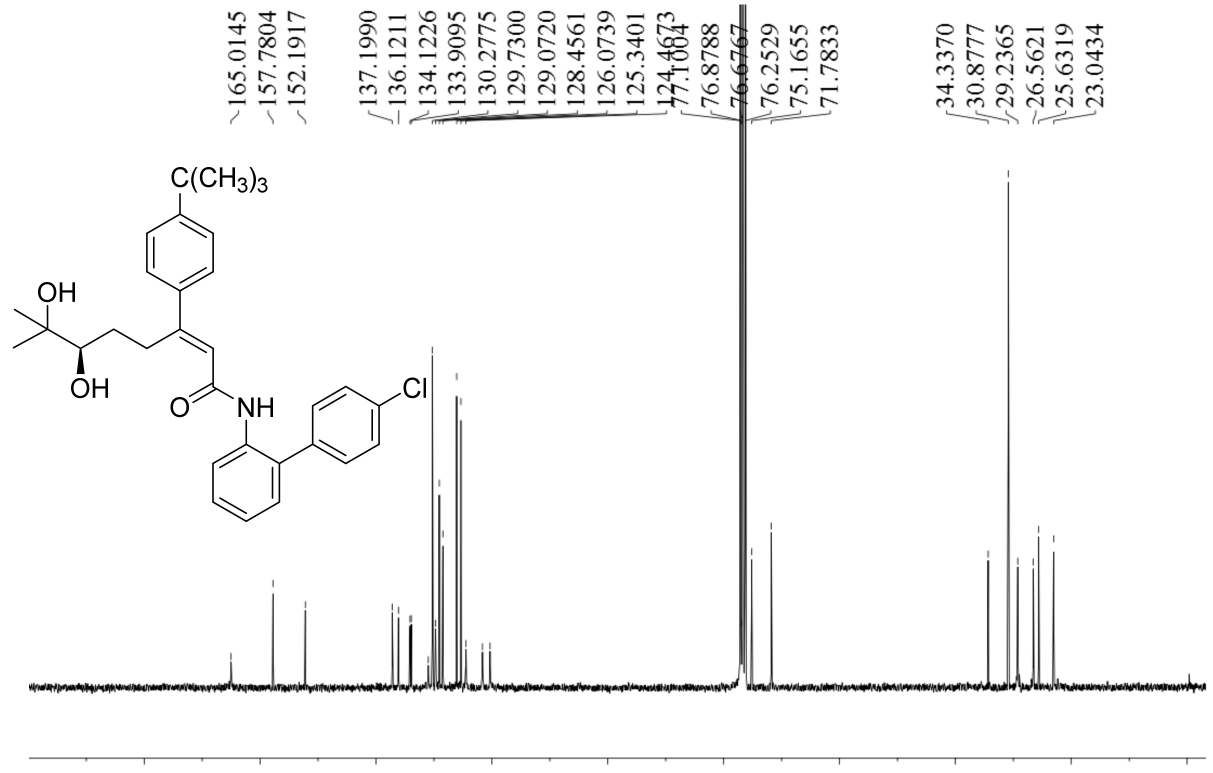

Figure S70. ^{13}C NMR spectrum of (*R*)-**11j**

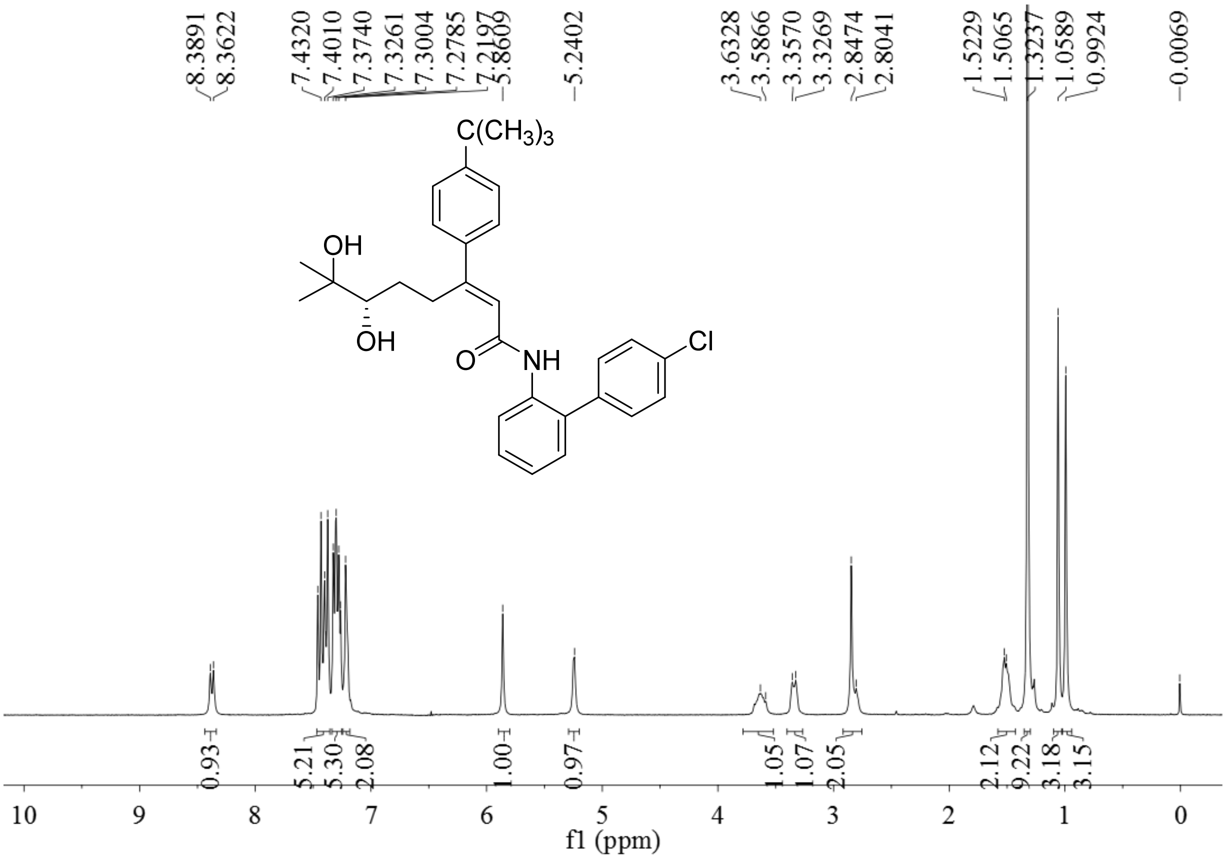

Figure S71. ^{1}H NMR of Compound (*S*)-**11j**

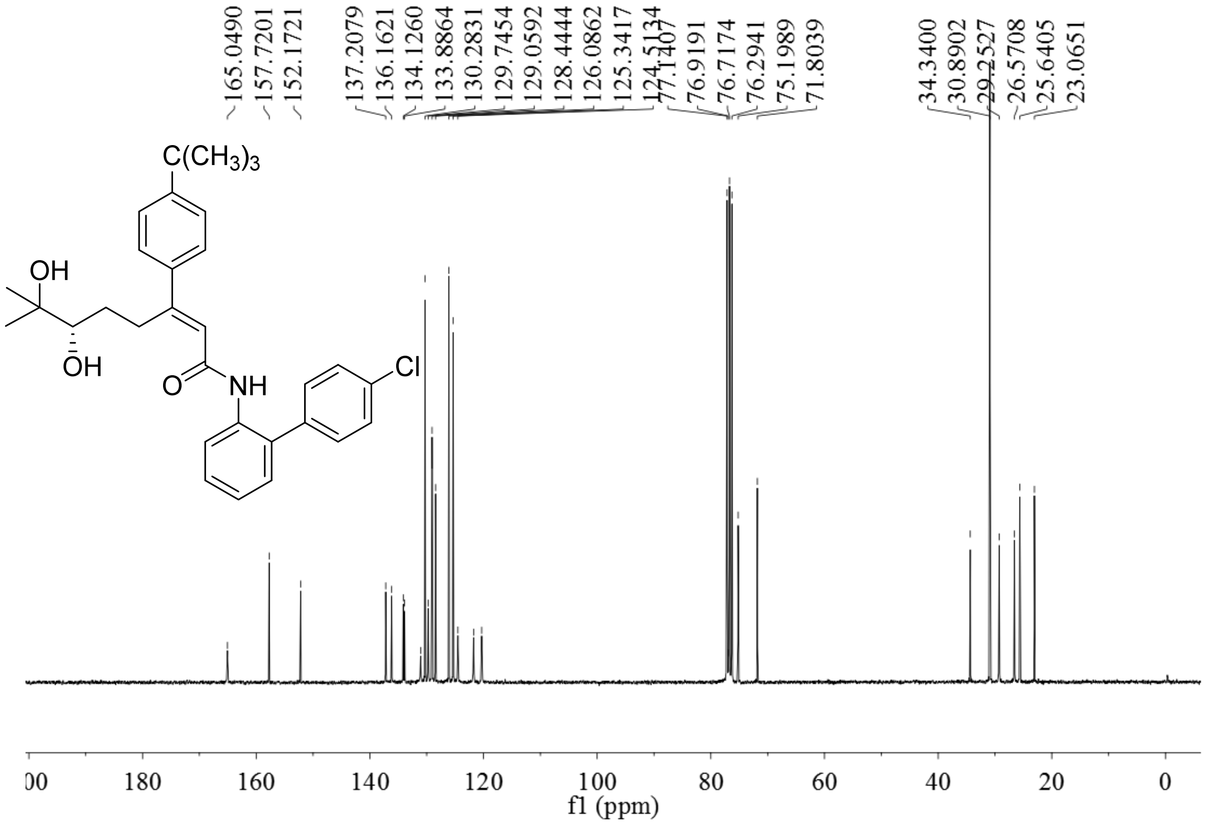

Figure S72. ^{13}C NMR spectrum of (*S*)-**11j**

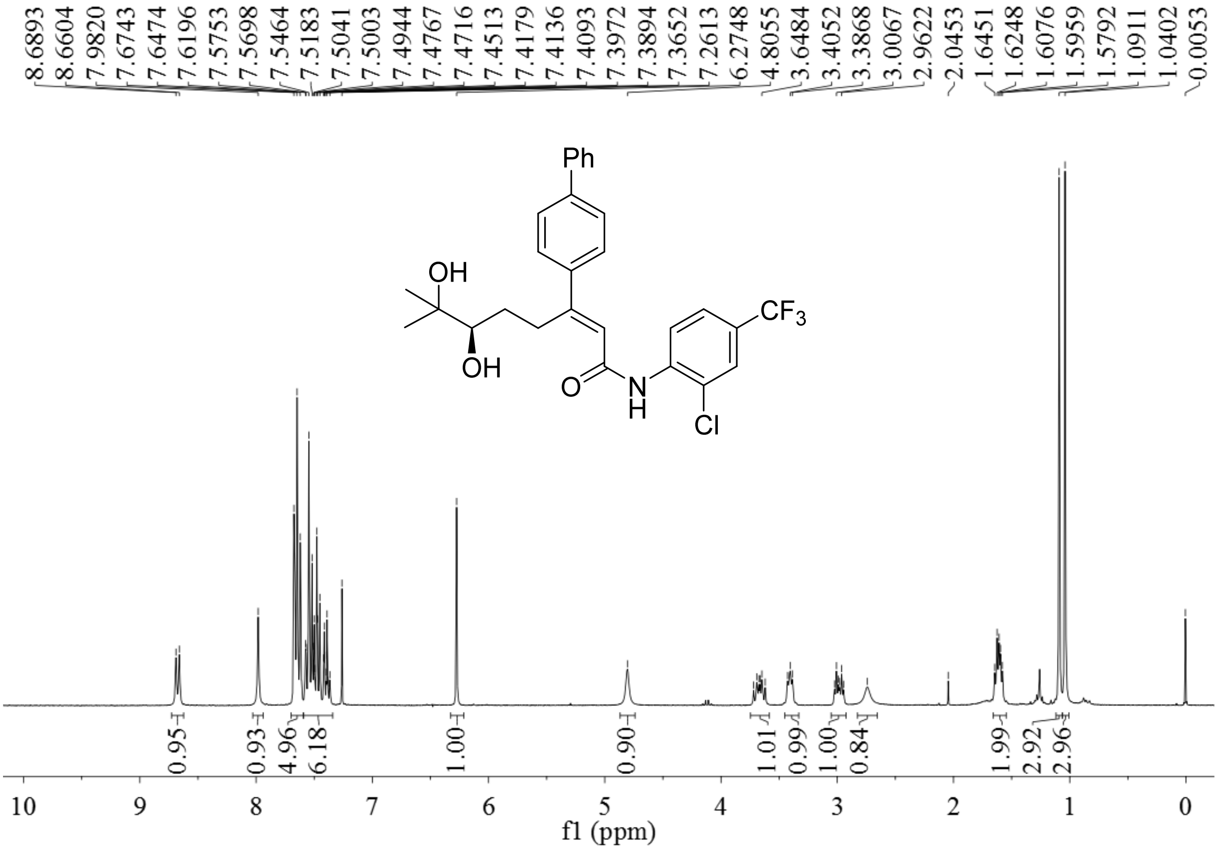

Figure S73. ^{1}H NMR of Compound (*R*)-**11k**

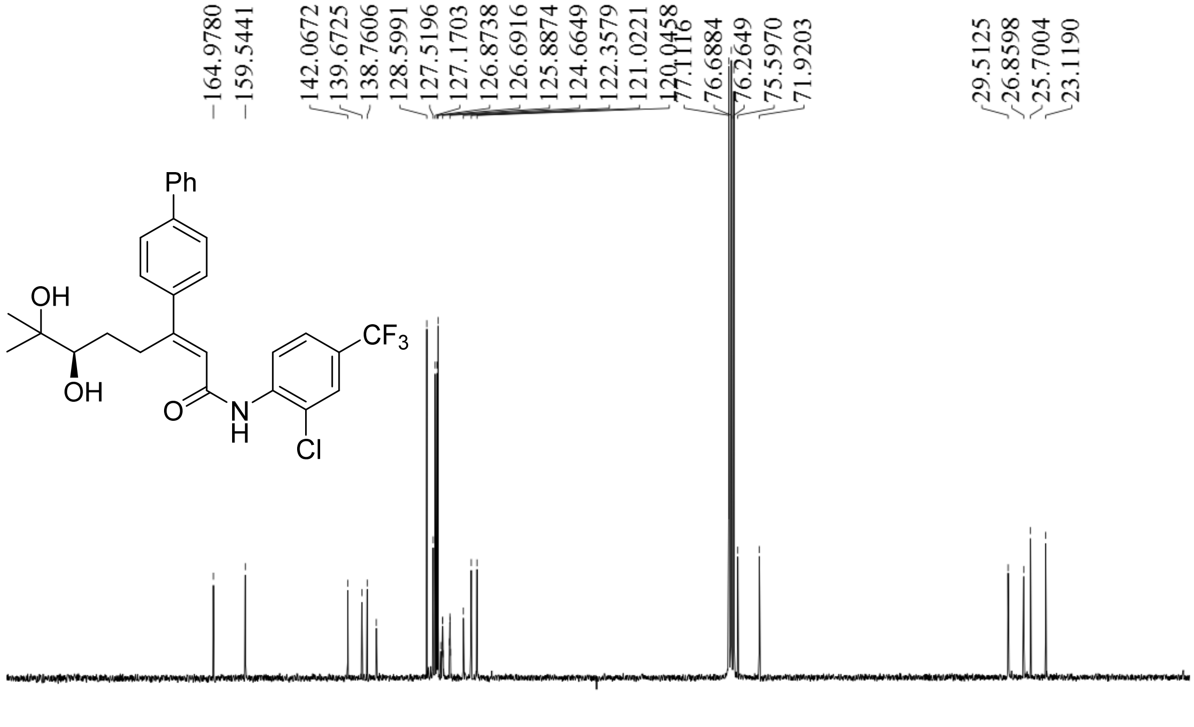

Figure S74. ^{13}C NMR spectrum of (*R*)-**11k**

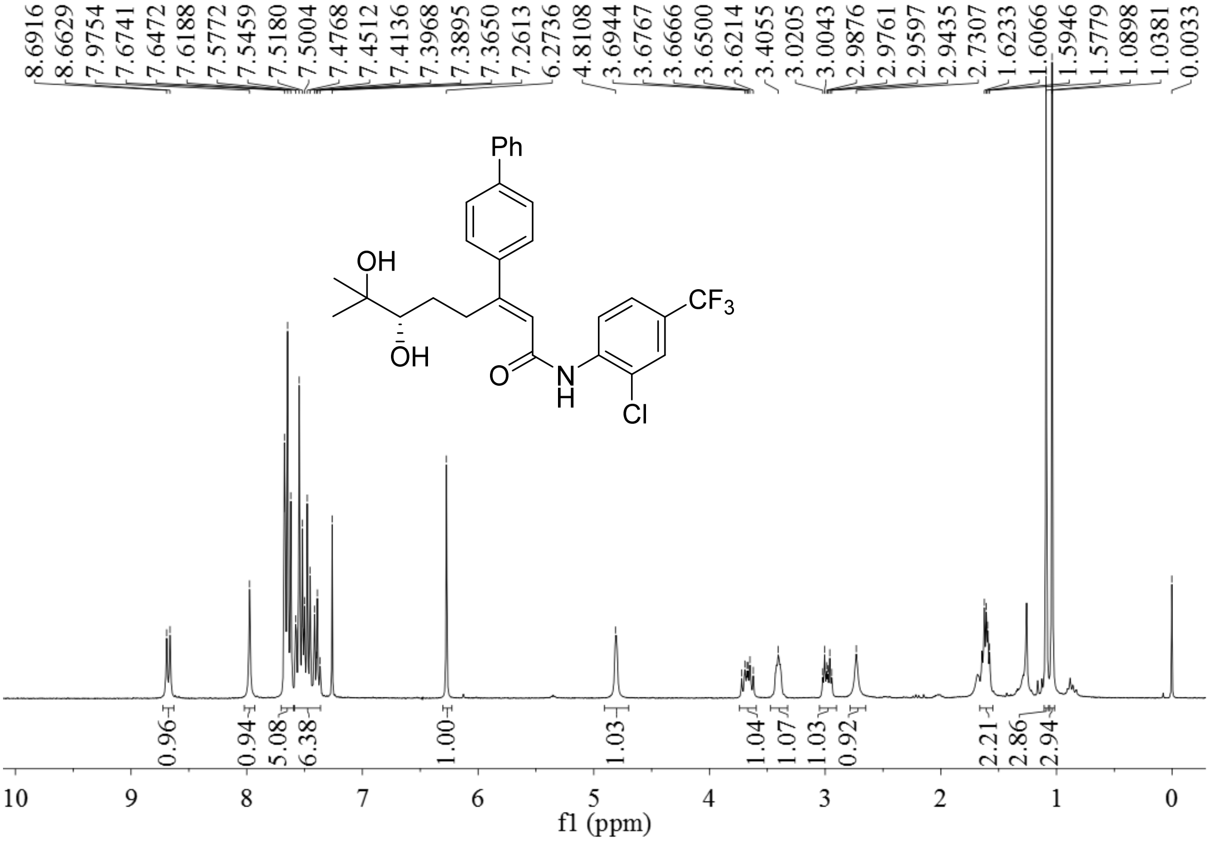

Figure S75. ^{1}H NMR of Compound (*S*)-**11k**

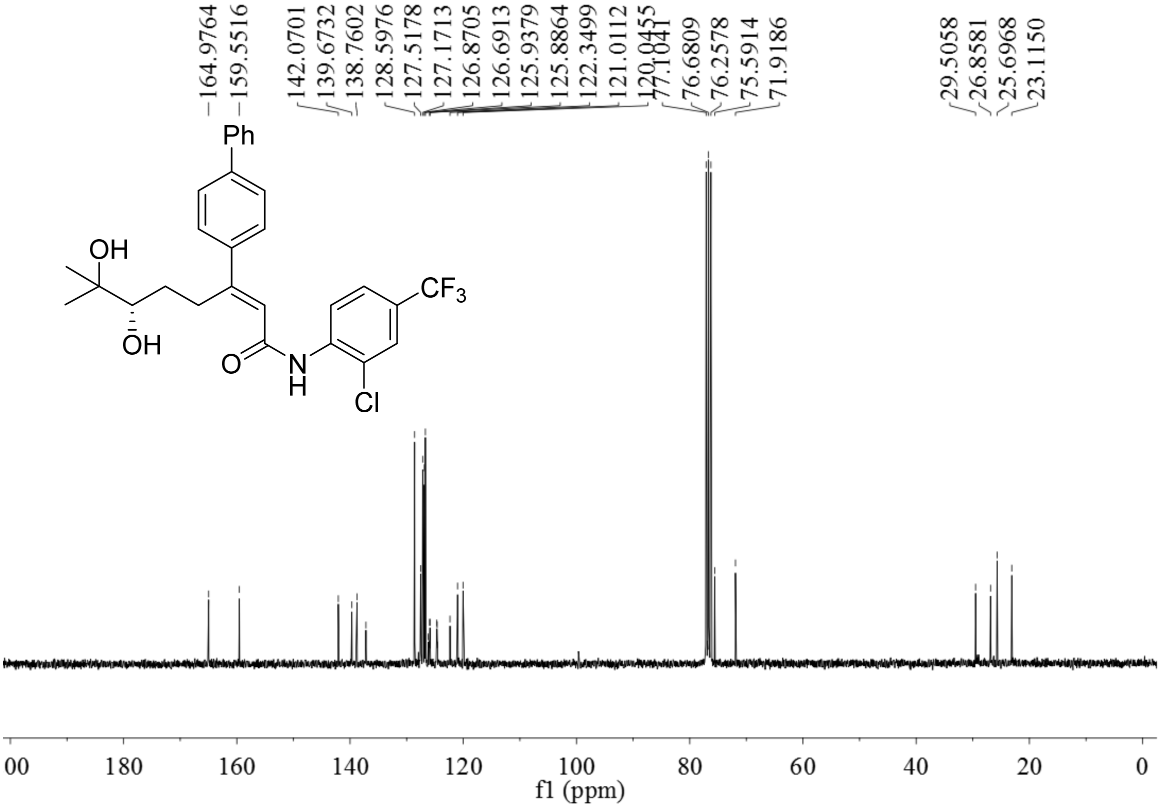

Figure S76. ^{13}C NMR spectrum of (*S*)-**11k**

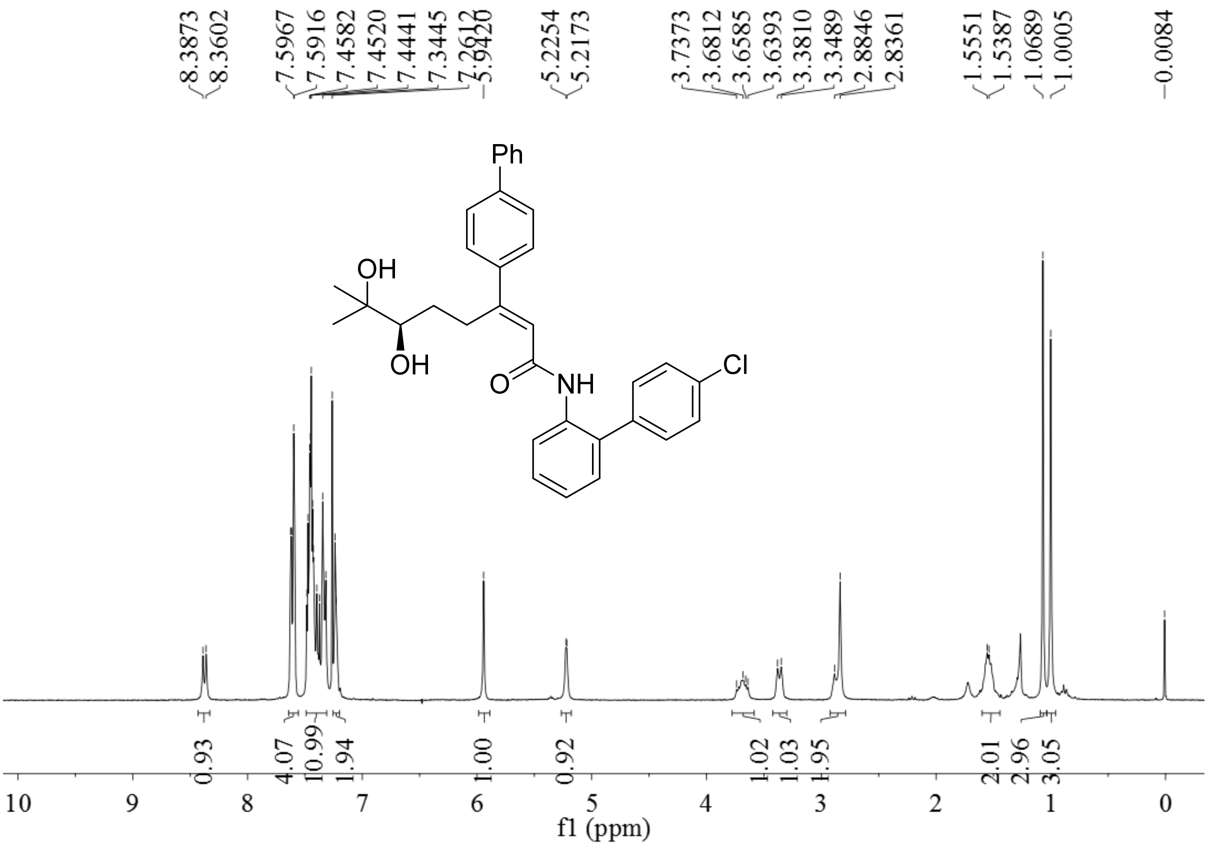

Figure S77. ^{1}H NMR of Compound (*R*)-**11l**

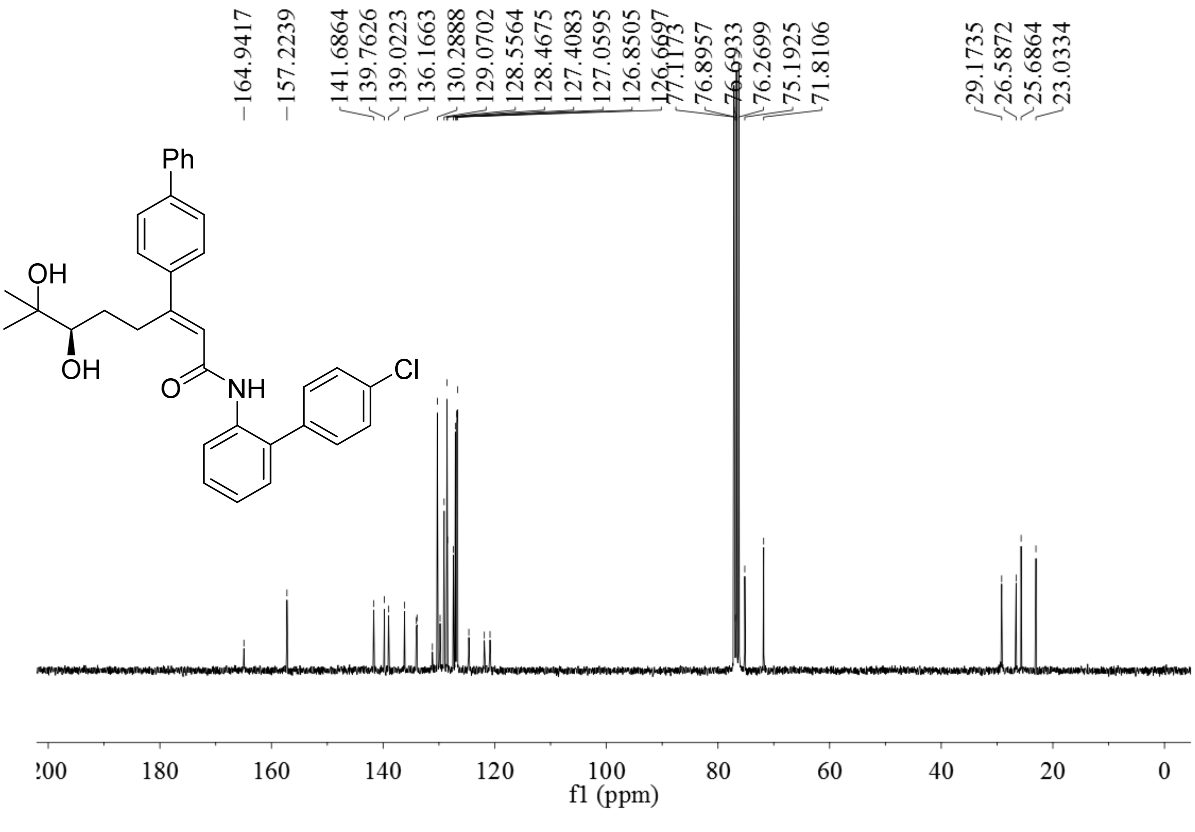

Figure S78. ^{13}C NMR spectrum of (*R*)-**11l**

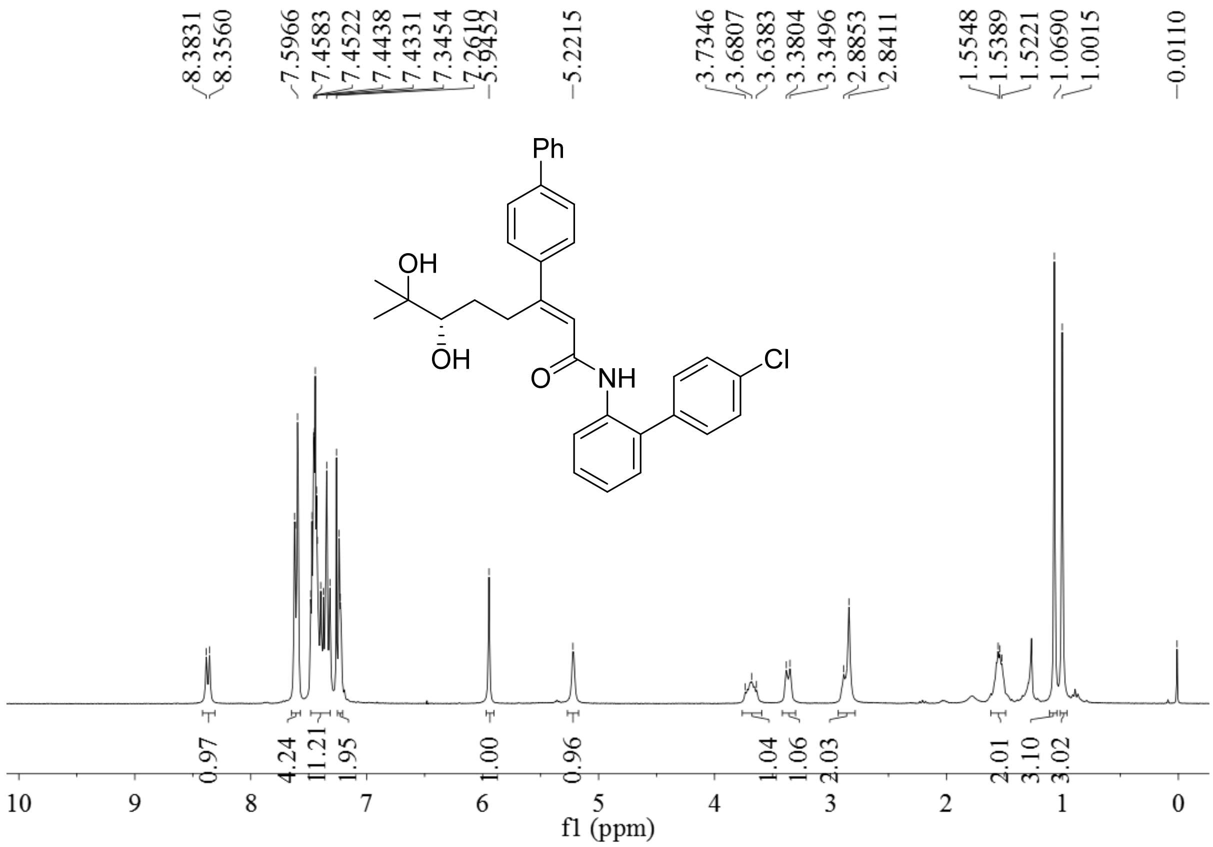

Figure S79. ^{1}H NMR of Compound (*S*)-**11l**

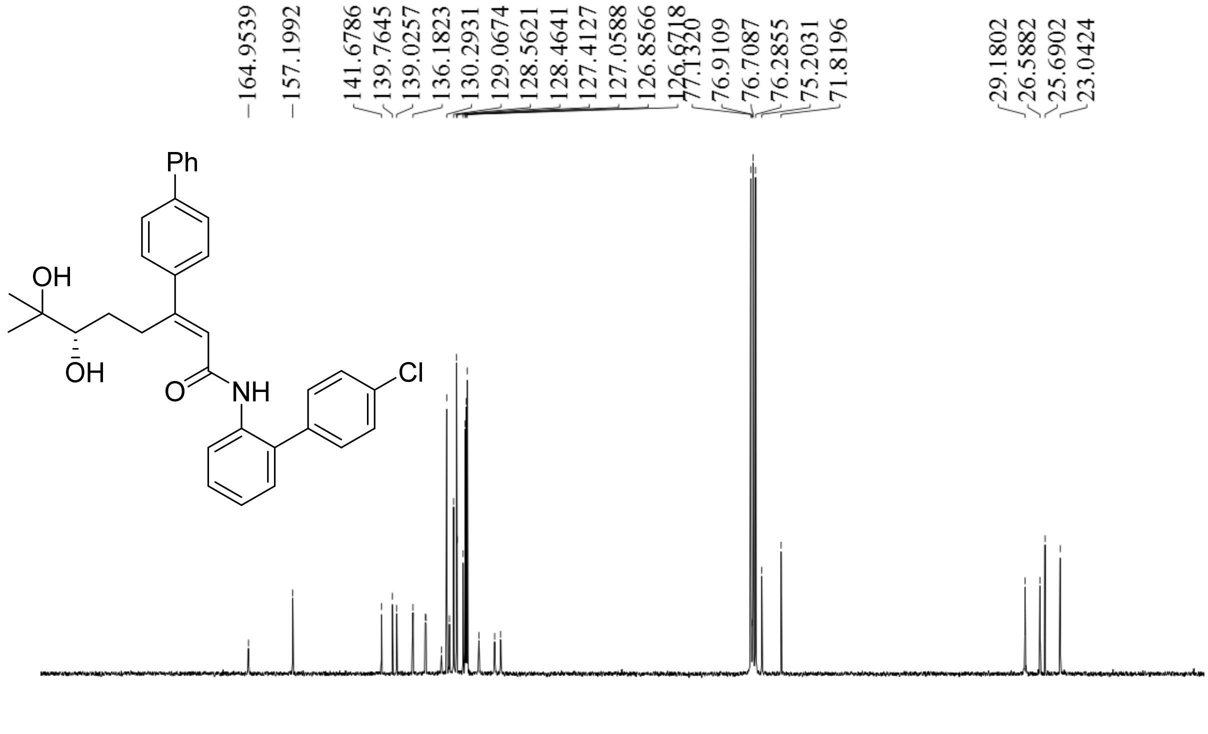

Figure S80. ^{13}C NMR spectrum of (*S*)-**11l**

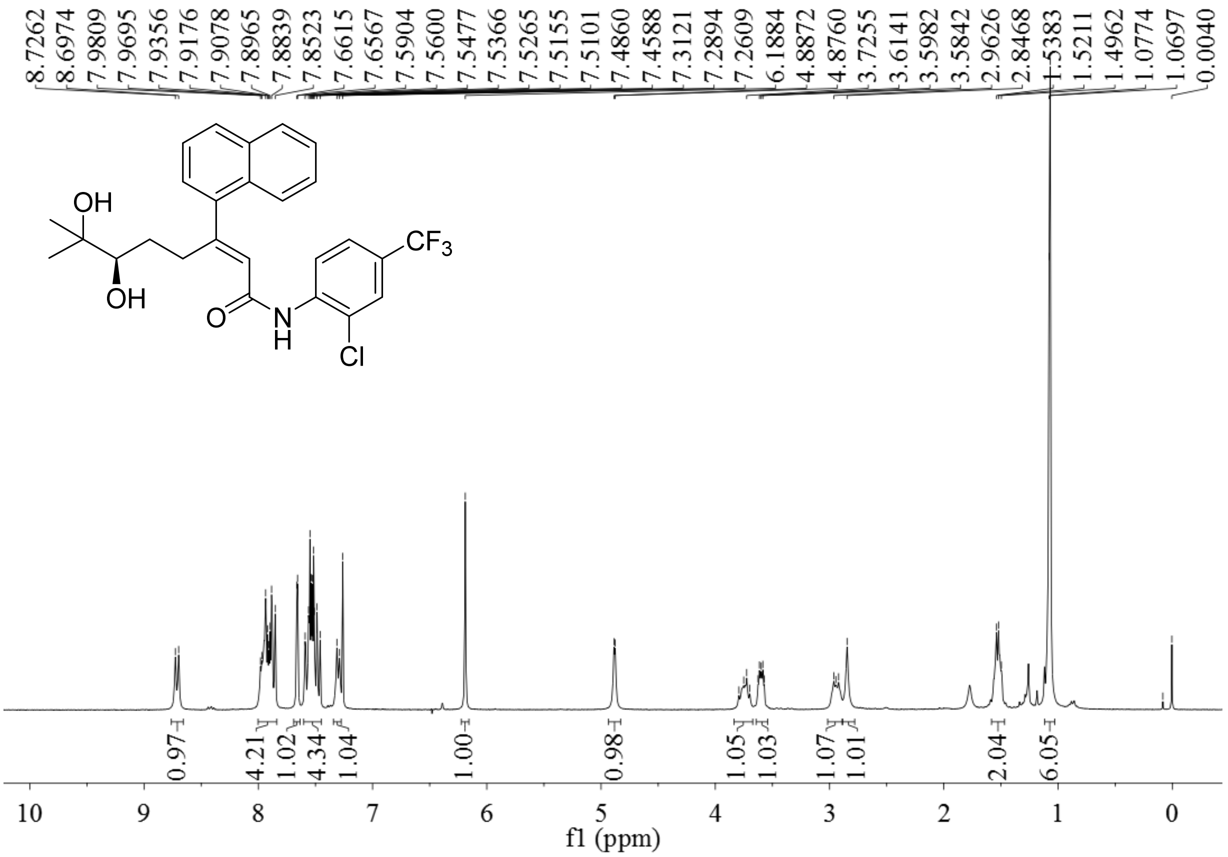

Figure S81. ^{1}H NMR of Compound (*R*)-**11m**

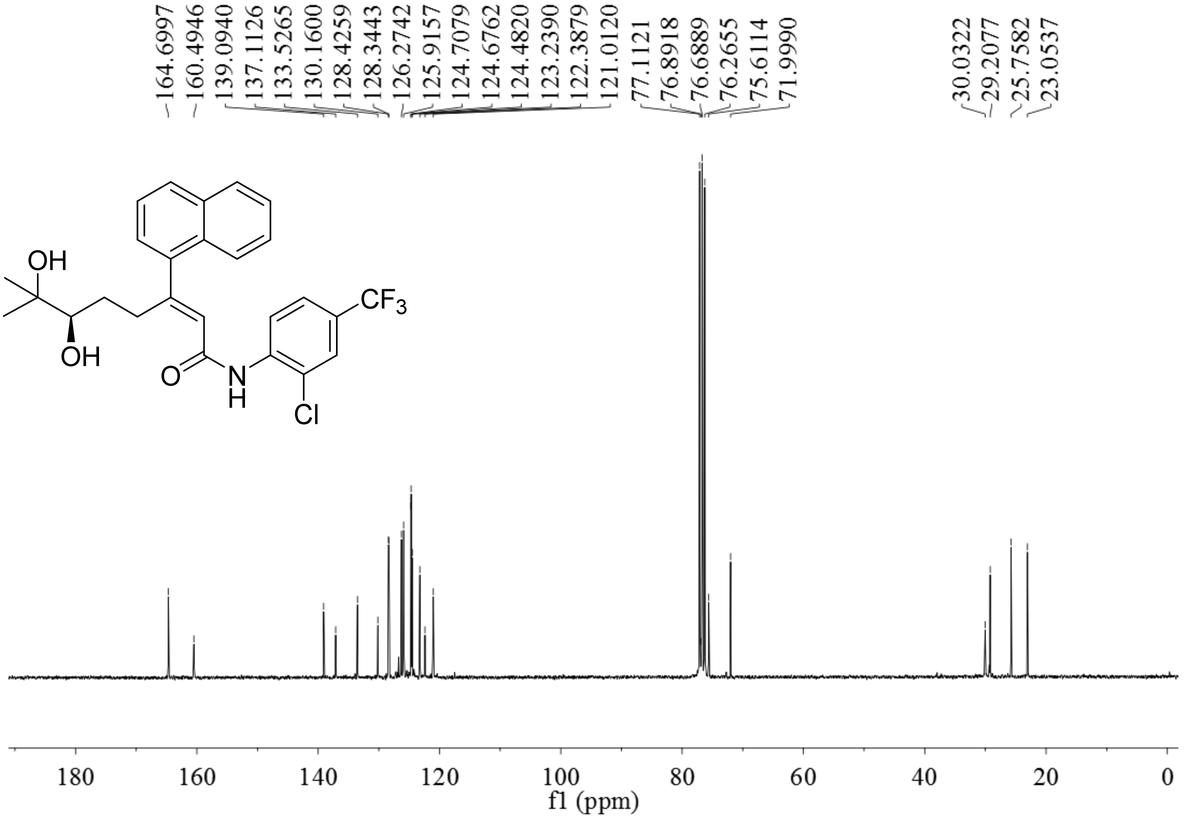

Figure S82. ^{13}C NMR spectrum of (*R*)-**11m**

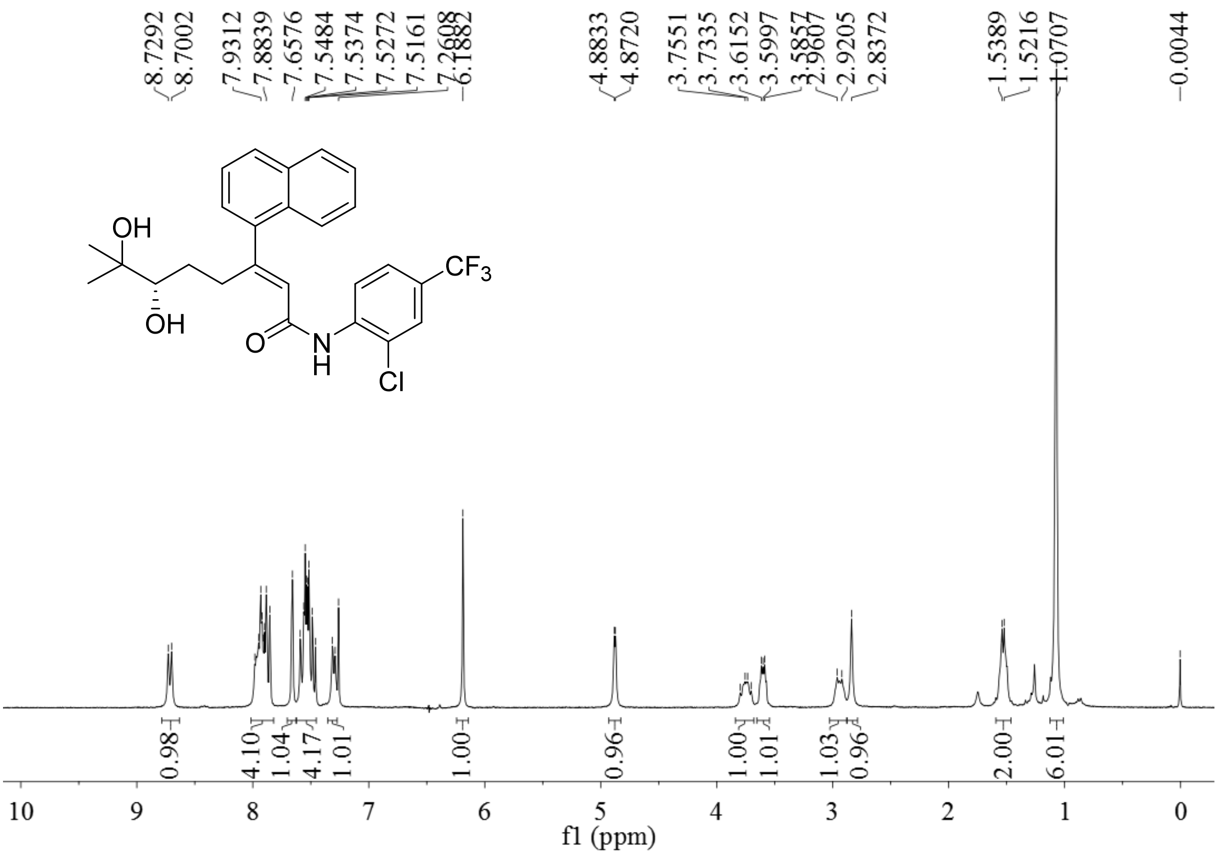

Figure S83. ^{1}H NMR of Compound (*S*)-**11m**

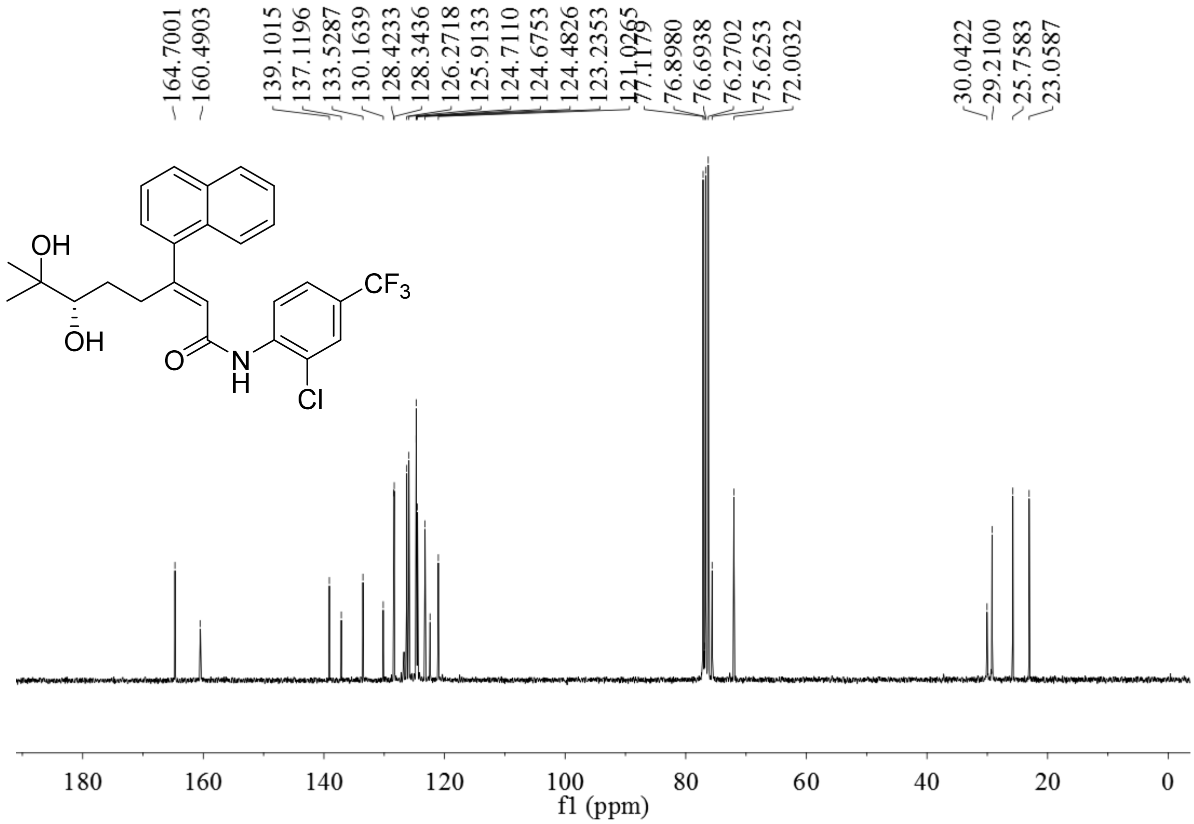

Figure S84. ^{13}C NMR spectrum of (*S*)-**11m**

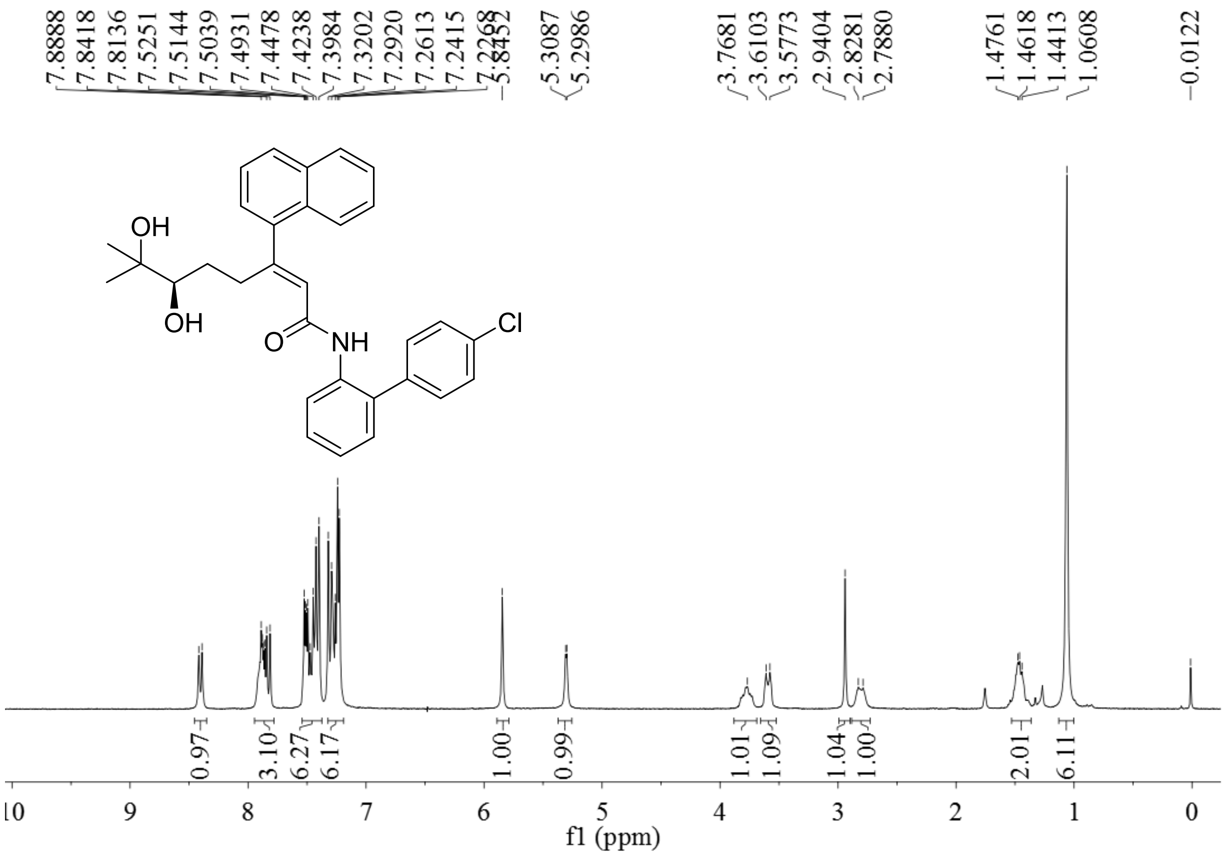

Figure S85. ^{1}H NMR of Compound (*R*)-**11n**

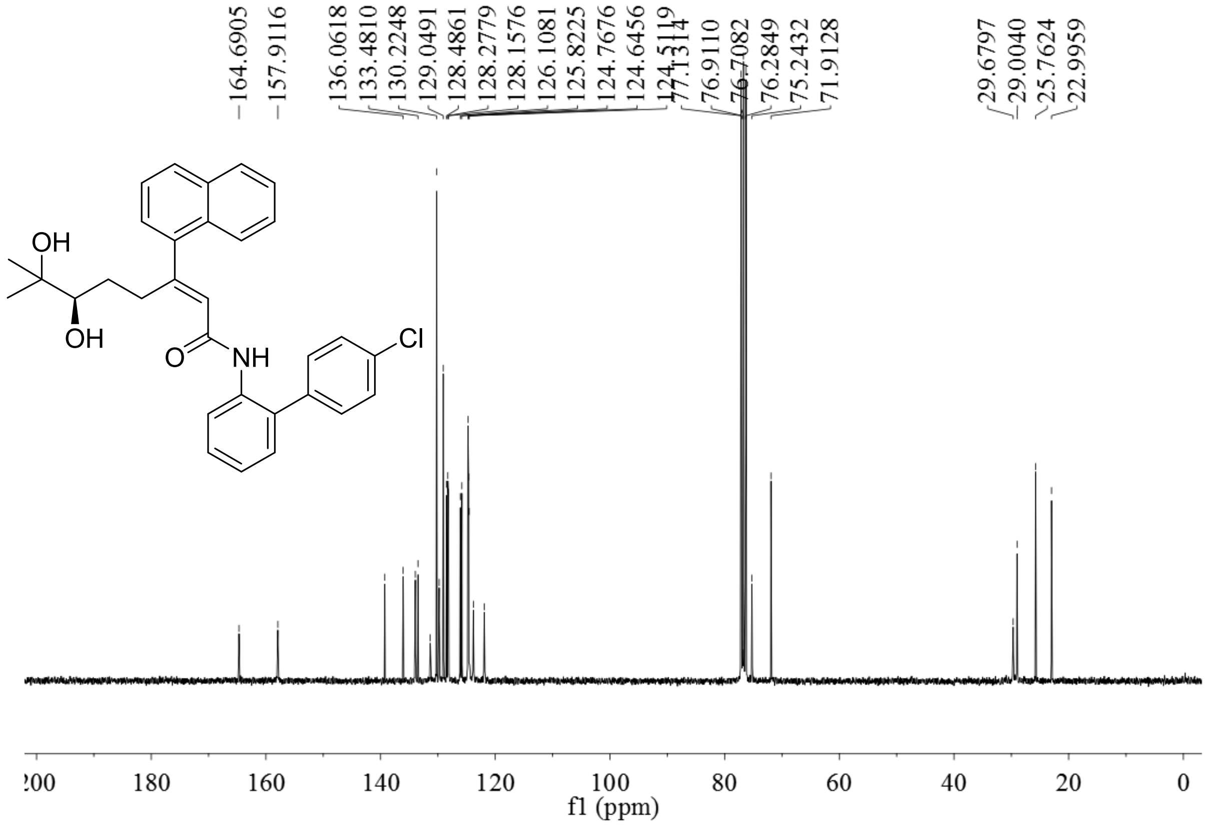

Figure S86. ^{13}C NMR spectrum of (*R*)-**11n**

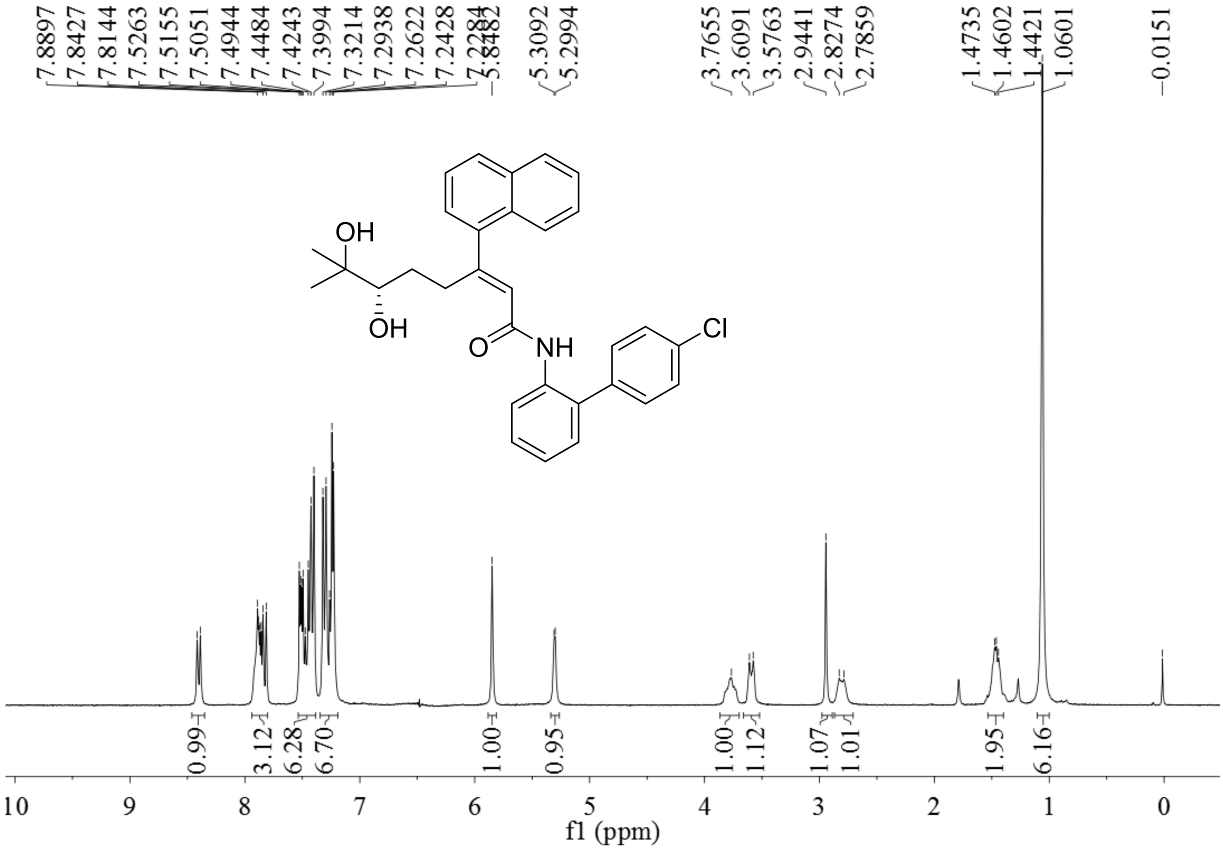

Figure S87. ^{1}H NMR of Compound (*S*)-**11n**

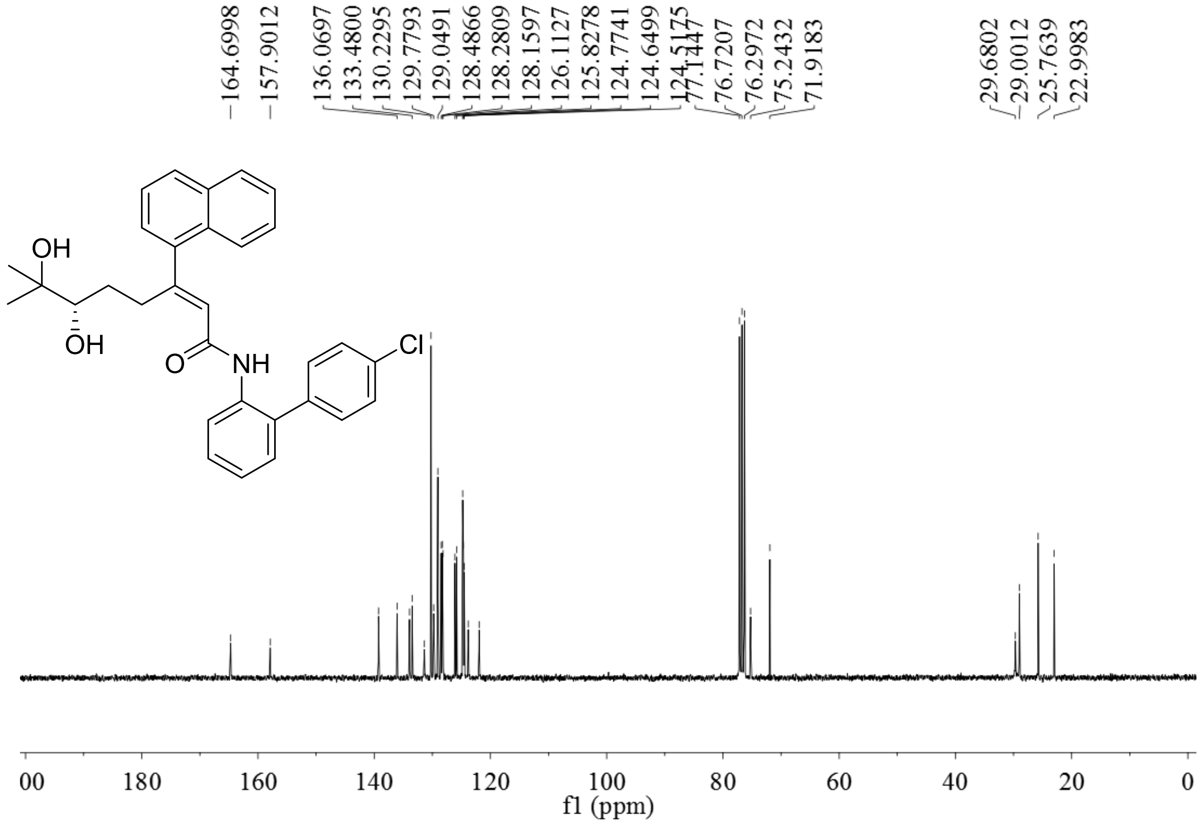

Figure S88. ^{13}C NMR spectrum of (*S*)-**11n**

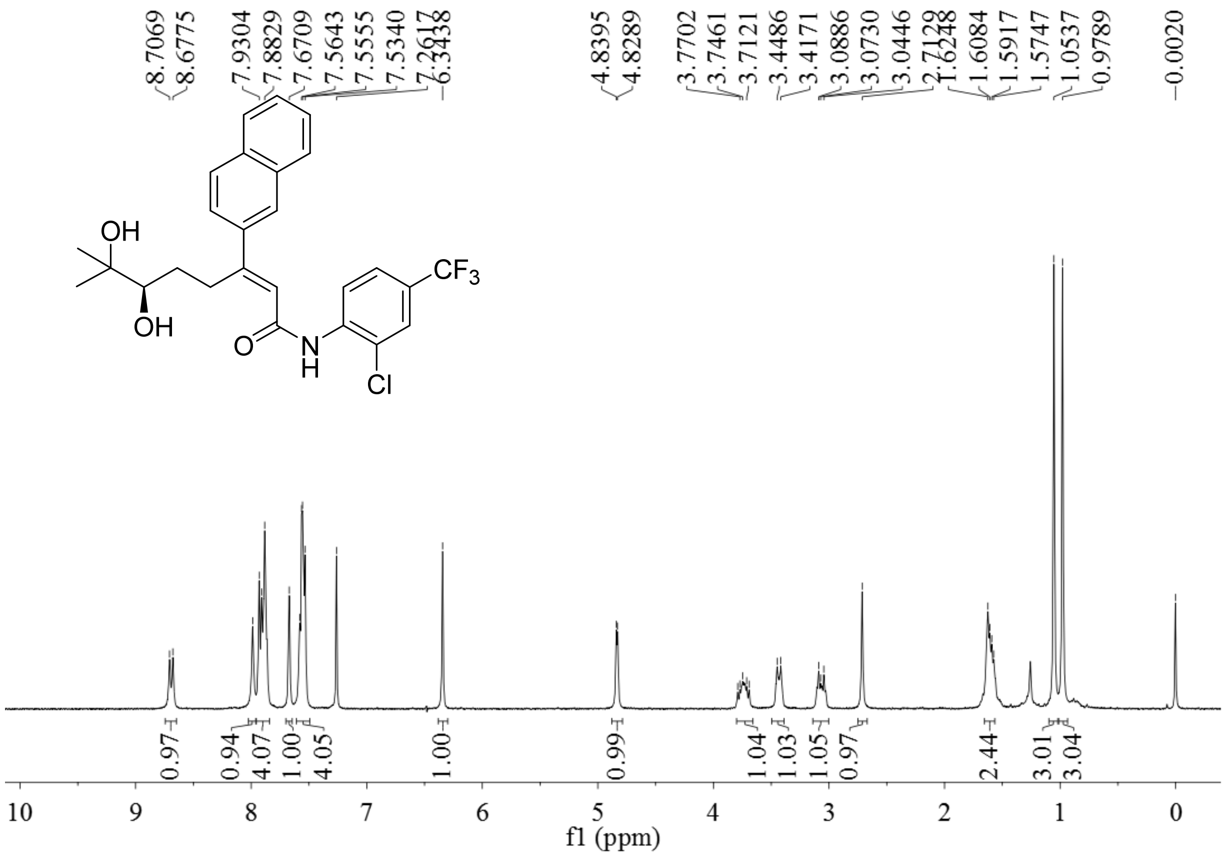

Figure S89. ^{1}H NMR of Compound (*R*)-**11o**

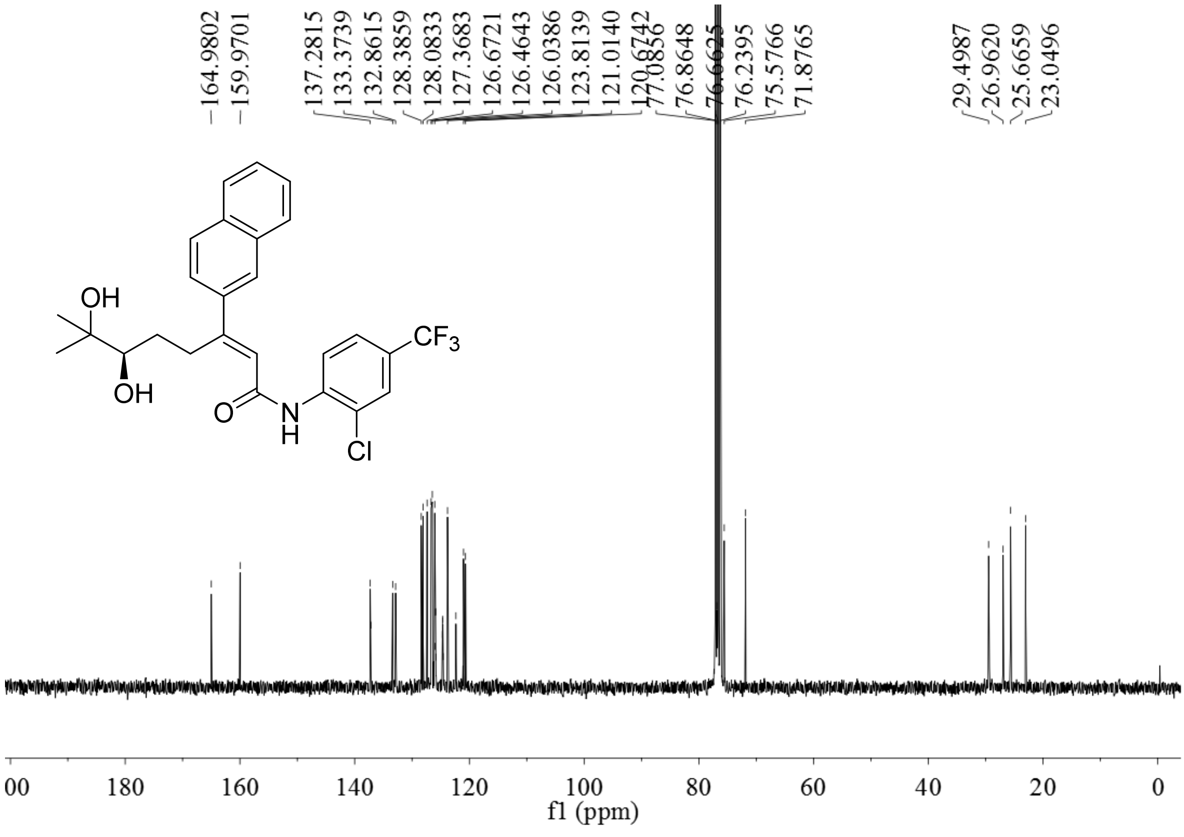

Figure S90. ^{13}C NMR spectrum of (*R*)-**11o**

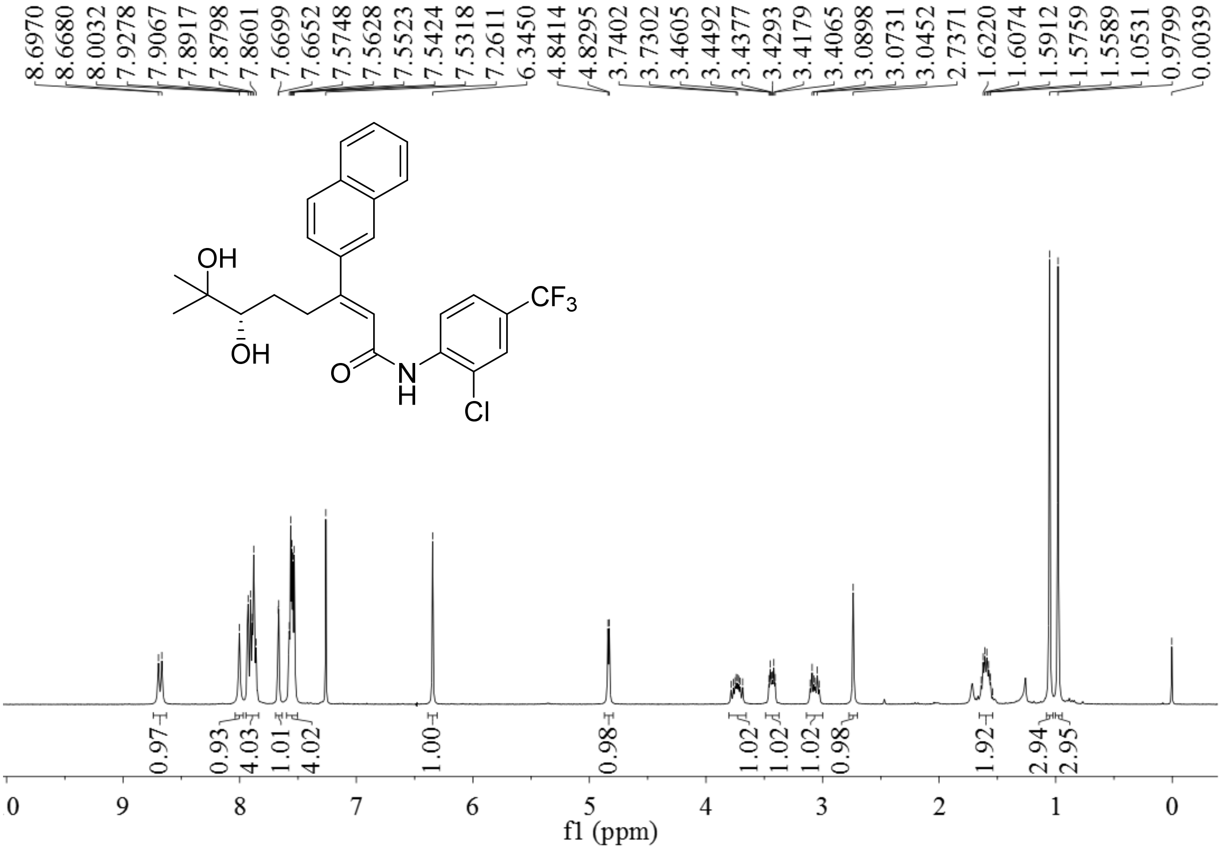

Figure S91. ^{1}H NMR of Compound (*S*)-**11o**

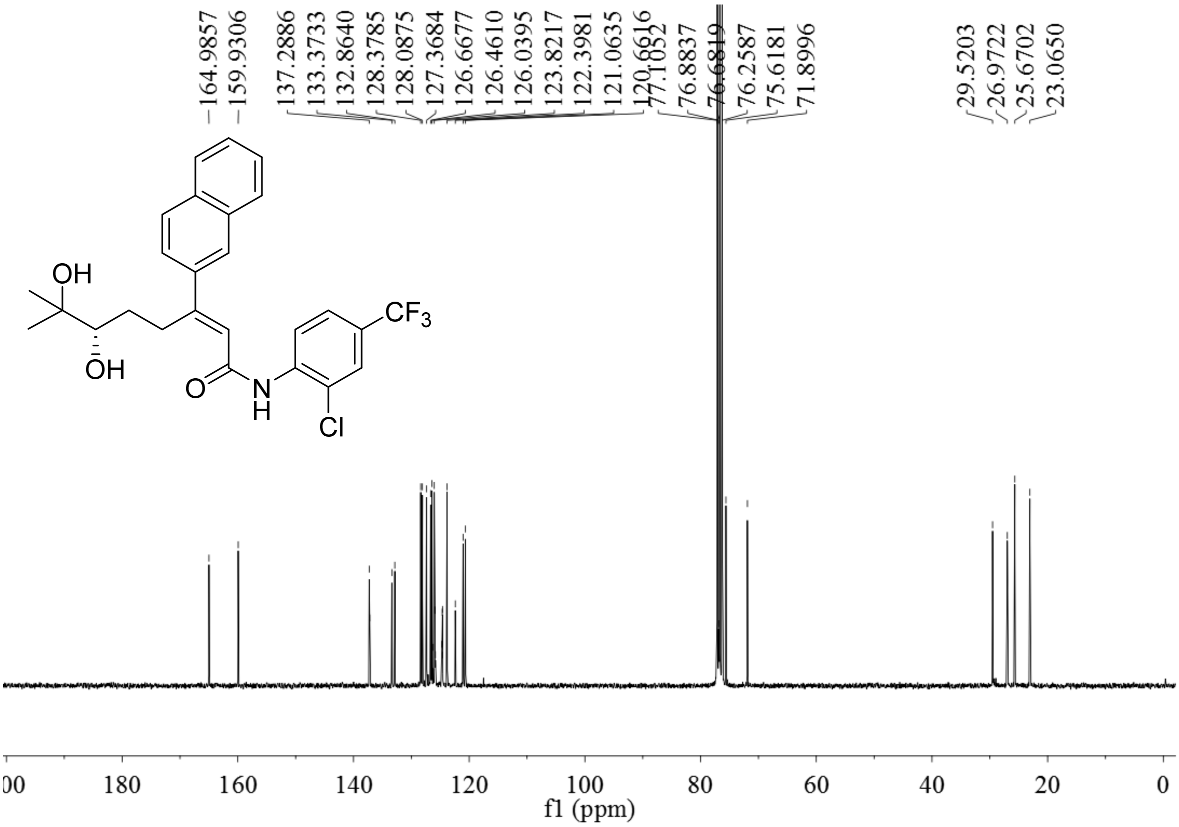

Figure S92. ^{13}C NMR spectrum of (*S*)-**11o**

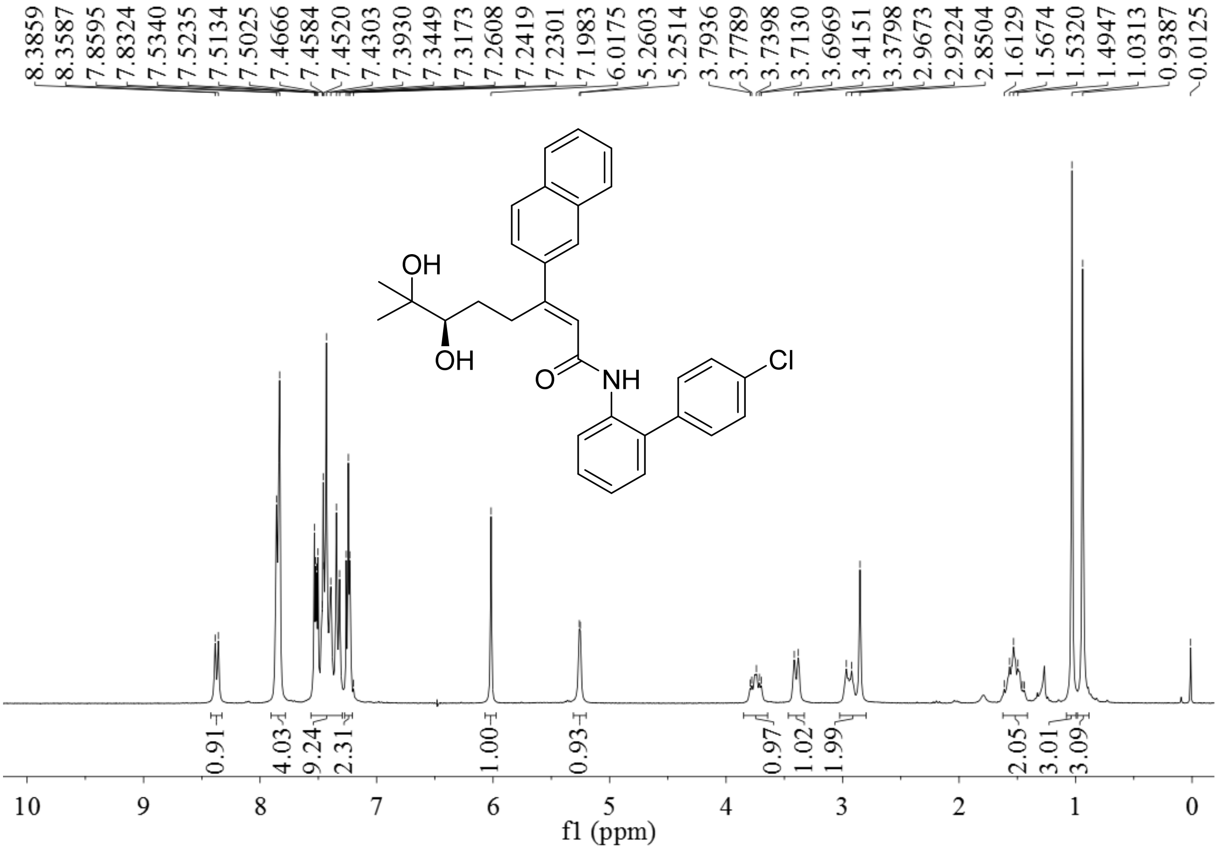

Figure S93. ^{1}H NMR of Compound (*R*)-**11p**

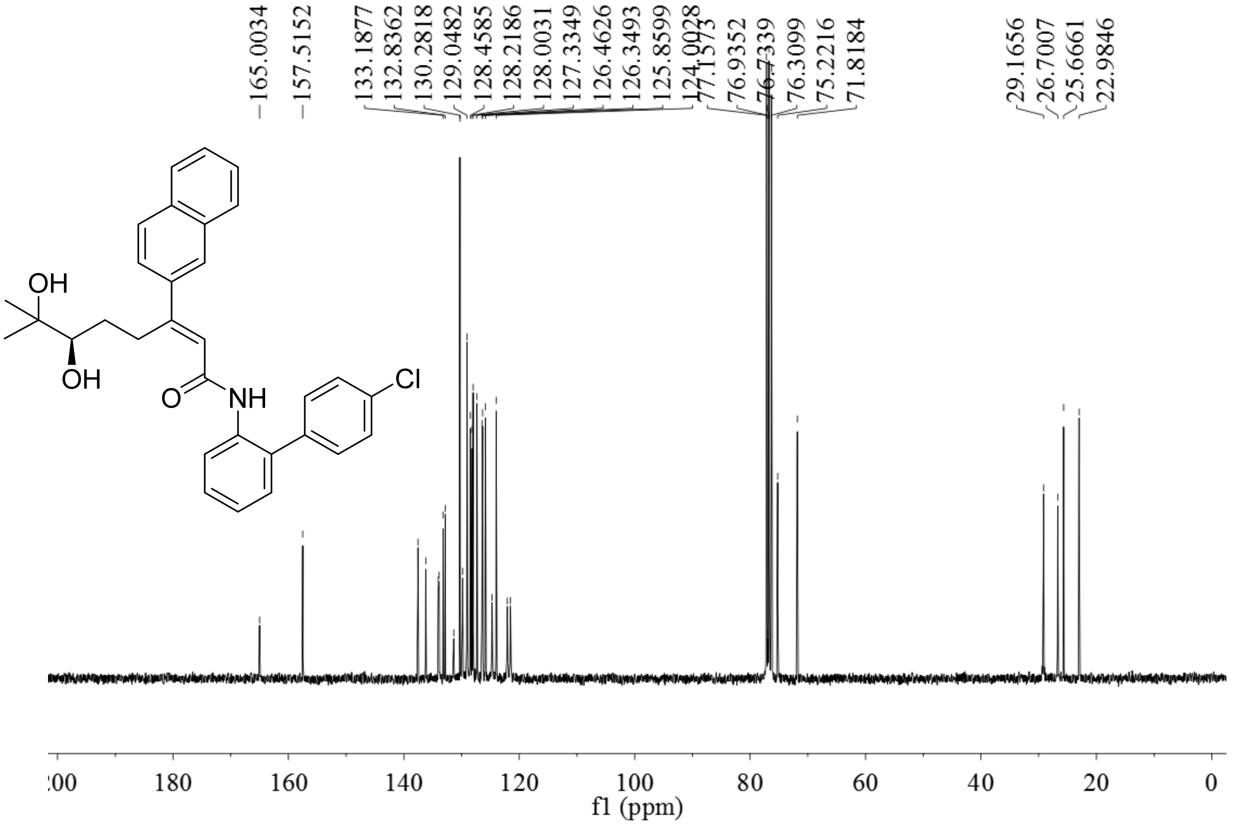

Figure S94. ^{13}C NMR spectrum of (*R*)-**11p**

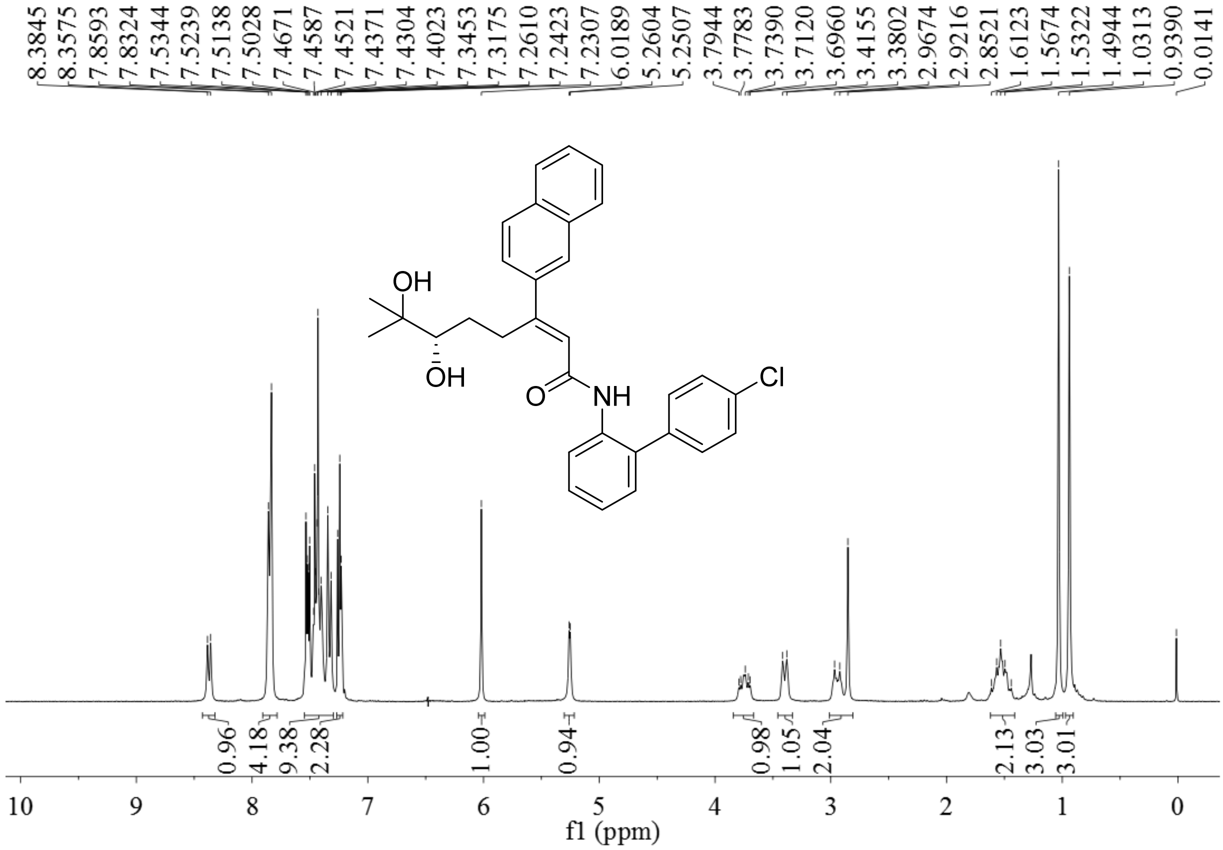

Figure S95. ^{1}H NMR of Compound (*S*)-**11p**

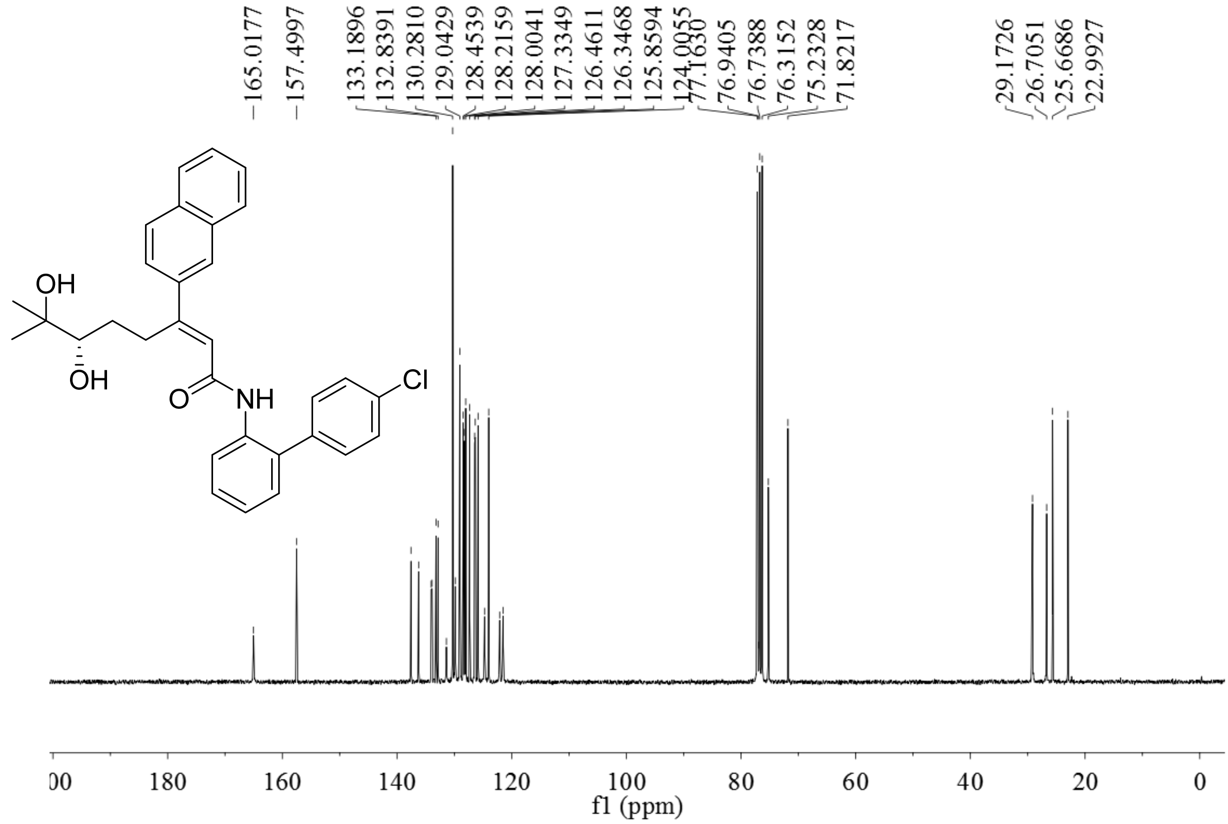

Figure S96. ^{13}C NMR spectrum of (*S*)-**11p**

2. HPLC profiles of 5 representative compounds

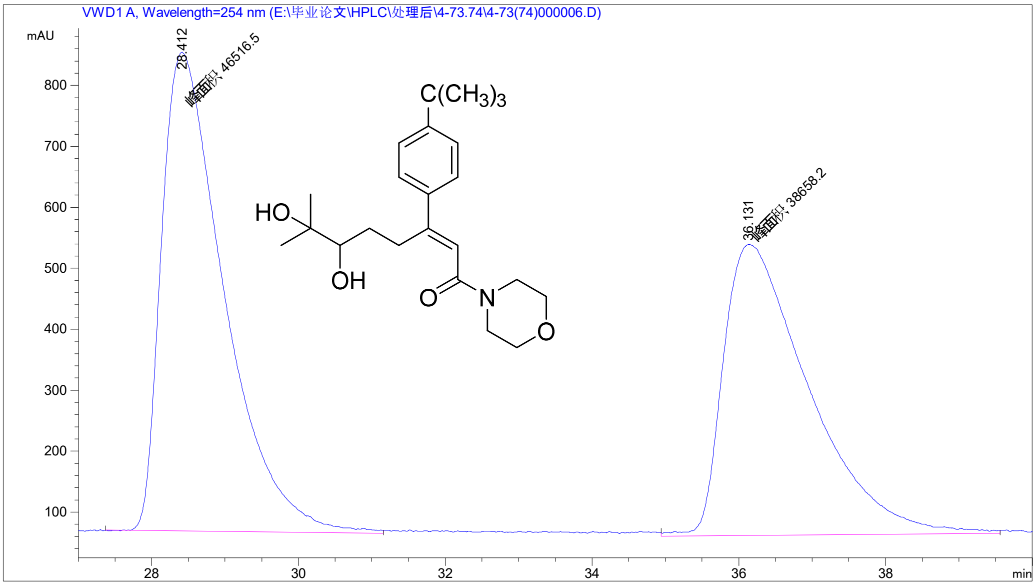

#	[min]		[min]	mAU	*s	[mAU]	%
1	28.412	MM	0.9873	4.65165e4		785.28253	54.6130
2	36.131	MM	1.3503	3.86582e4		477.13879	45.3870

Figure S97. HPLC profile of racemic **11a**

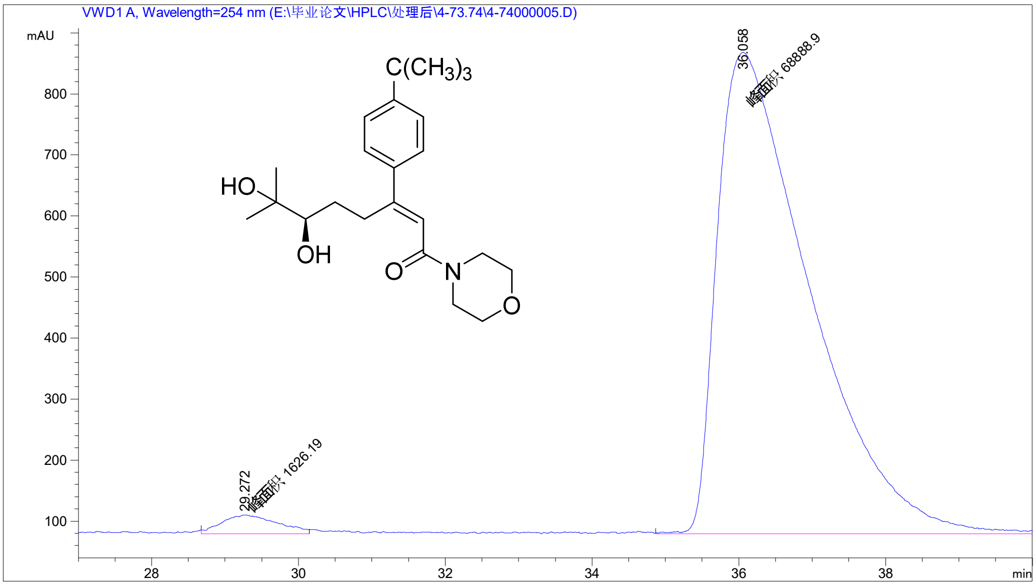

#	[min]		[min]	mAU	*s	[mAU]	%
1	29.272	MM	0.8818	1626.18958		30.73521	2.3062
2	36.058	MM	1.4556	6.88889e4		788.76563	97.6938

Figure S98. HPLC profile of (*R*)-**11a**

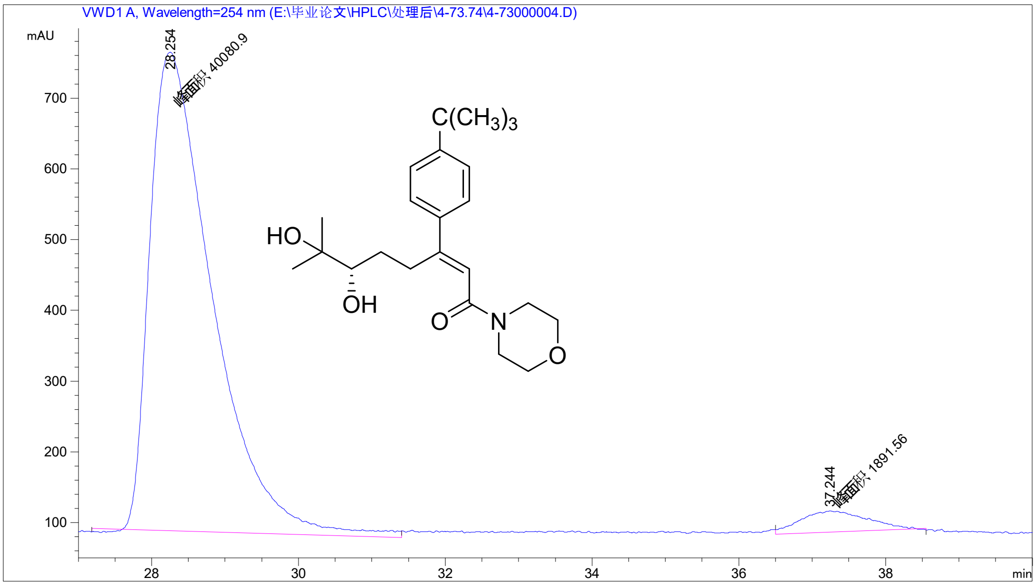

[min] [min] mAU *s [mAU] %

----|-------|----|-------|----------|----------|--------|

1	28.254 MM	0.9883	4.00809e4	675.89410	95.4933
2	37.244 MM	1.0554	1891.55688	29.87071	4.5067

Figure S99. HPLC profile of (*S*)-**11a**

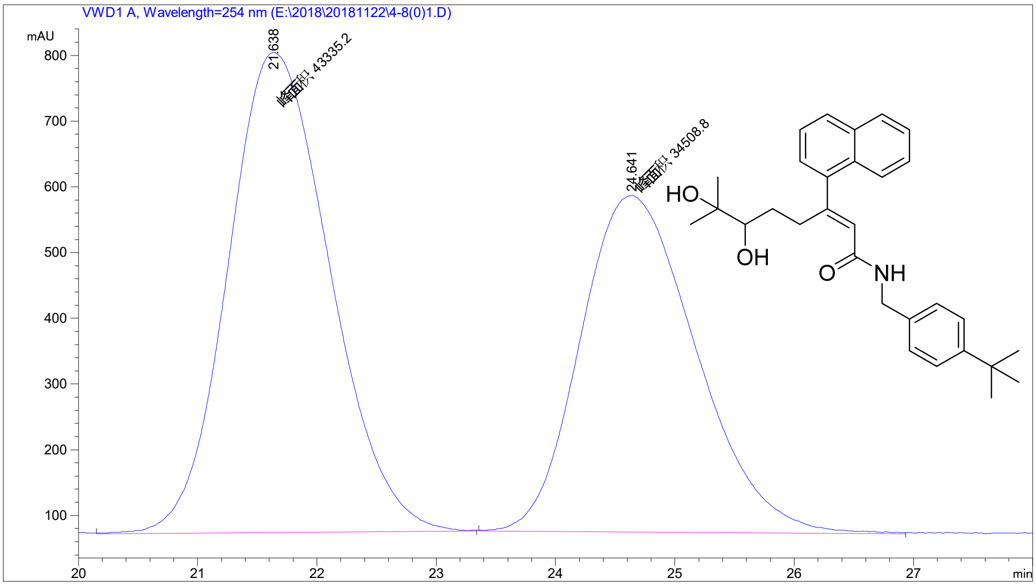

[min] [min] mAU *s [mAU] %

----|-------|----|-------|----------|----------|--------|

1	21.638 MM	0.9884	4.33352e4	730.69397	55.6693
2	24.641 MM	1.1236	3.45088e4	511.88162	44.3307

Figure S100. HPLC profile of racemic **11f**

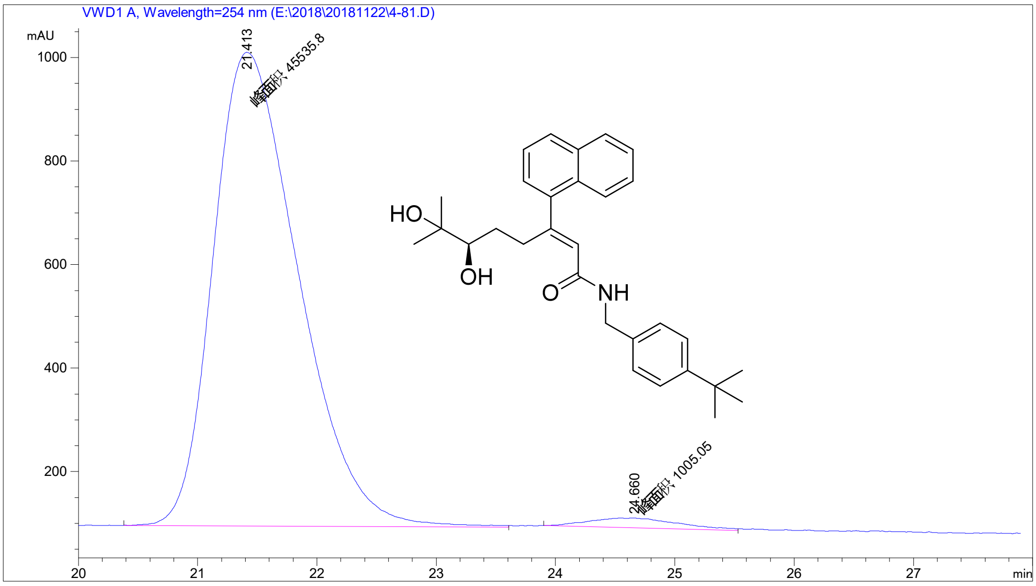

#	[min]		[min]	mAU *s	[mAU]	%
----|	-------|	----|	-------|	----------|	----------|	--------|
1	21.413	MM	0.8289	4.55358e4	915.61072	97.8405
2	24.660	MM	0.8785	1005.05499	19.06817	2.1595

Figure S101. HPLC profile of (*R*)-**11f**

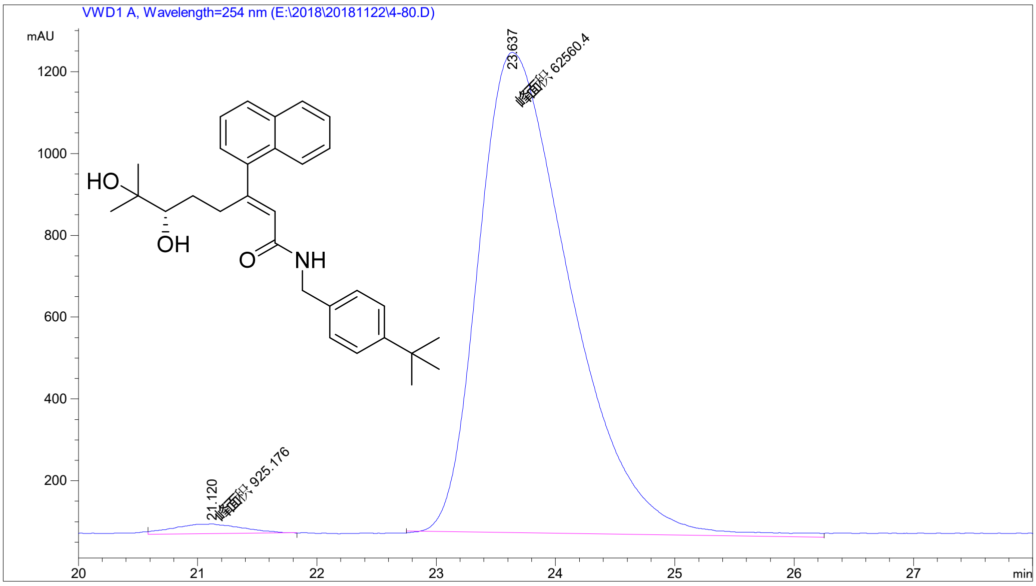

#	[min]		[min]	mAU *s	[mAU]	%
----|	-------|	----|	-------|	----------|	----------|	--------|
1	21.120	MM	0.6481	925.17633	23.79377	1.4573
2	23.637	MM	0.8884	6.25604e4	1173.59363	98.5427

Figure S102. HPLC profile of (*S*)-**11f**

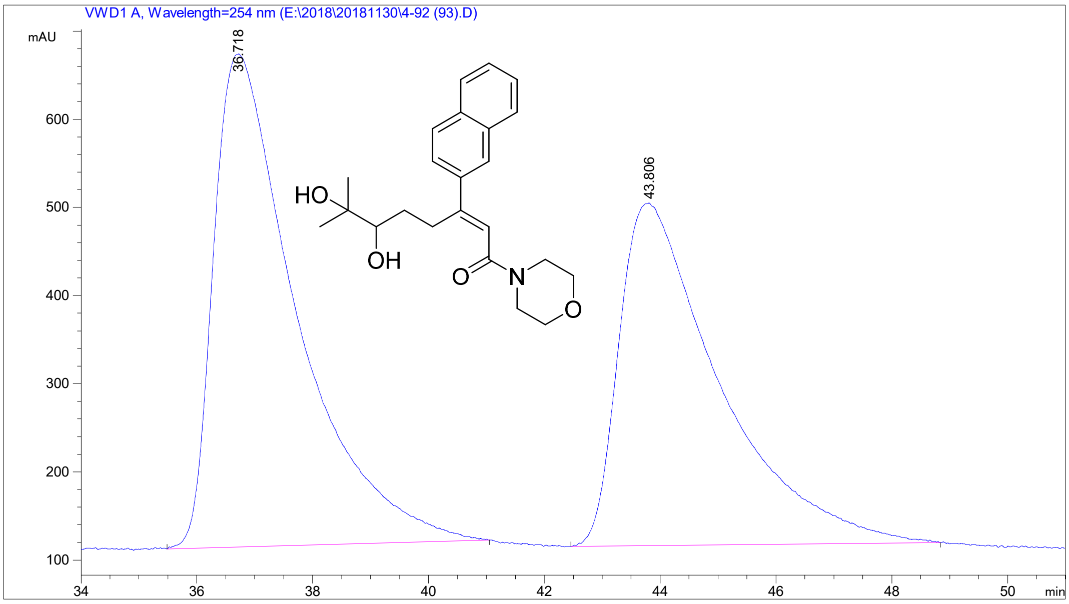

#	[min]		[min]	mAU *s	[mAU]	%
	----|-------|----|-------|----------|----------|--------|					
1	36.718	BB	1.2928	5.59493e4	559.03369	54.4429
2	43.806	BB	1.4169	4.68176e4	388.69717	45.5571

Figure S103. HPLC profile of racemic **11g**

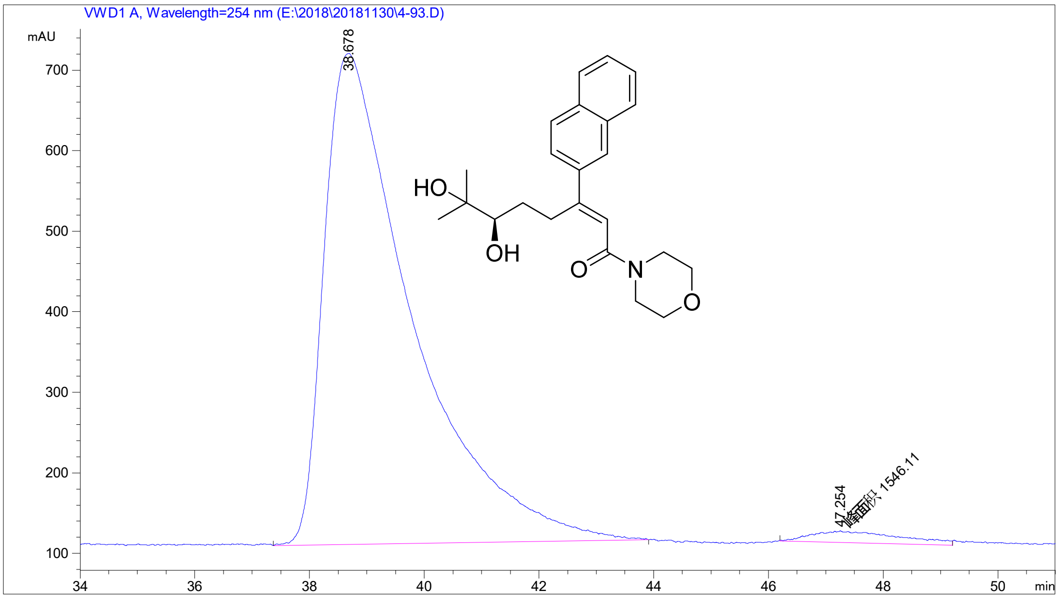

#	[min]		[min]	mAU *s	[mAU]	%
	----|-------|----|-------|----------|----------|--------|					
1	38.678	BB	1.4721	6.50230e4	609.31818	97.6774
2	47.254	MM	1.8103	1546.10559	14.23401	2.3226

Figure S104. HPLC profile of (*R*)-**11g**

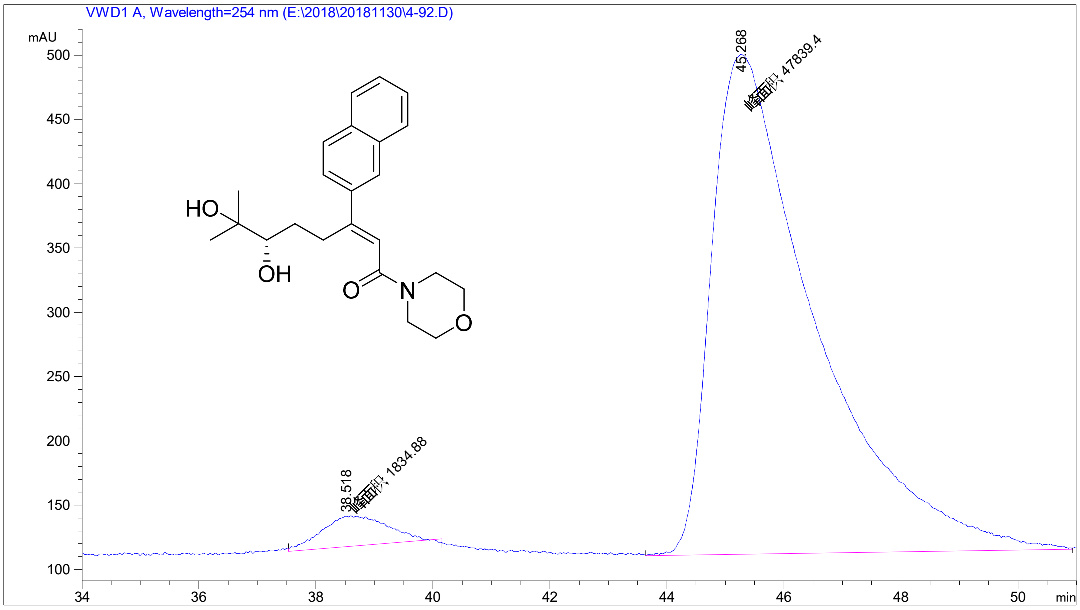

#	[min]		[min]	mAU	*s	[mAU	]	%
----|	-------|	----|	-------|	----------|	----------|	--------|		
1	38.518	MM	1.2862	1834.87976	23.77638	3.6938		
2	45.268	MM	2.0491	4.78394e4	389.11182	96.3062		

Figure S105. HPLC profile of (*S*)-**11g**

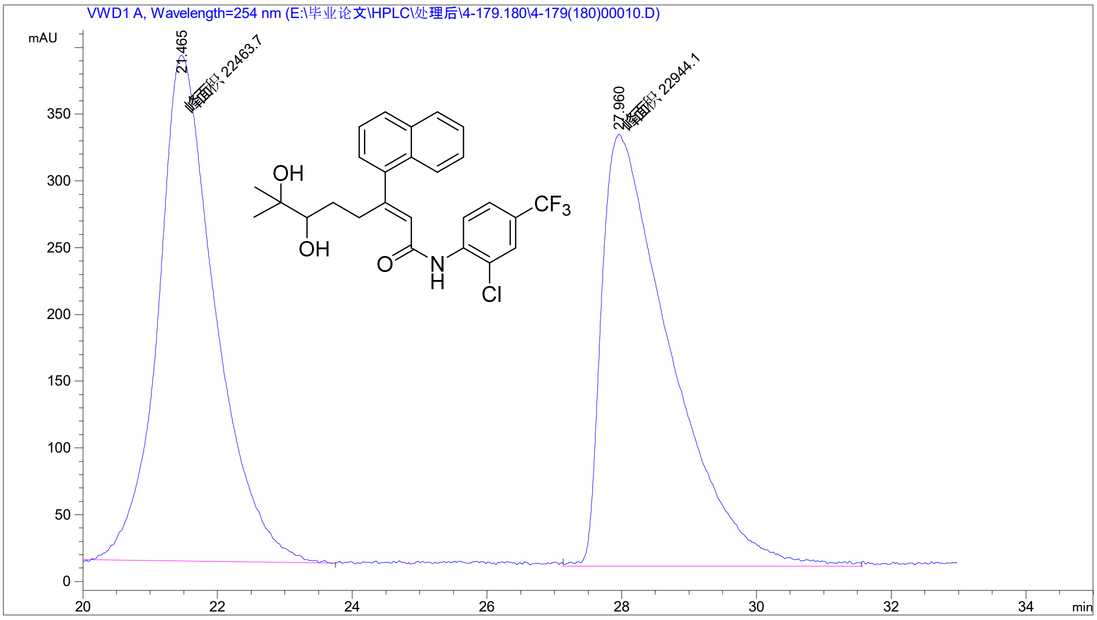

#	[min]		[min]	mAU	*s	[mAU	]	%
----|	-------|	----|	-------|	----------|	----------|	--------|		
1	21.465	MM	0.9881	2.24637e4	378.90637	49.4711		
2	27.960	MM	1.1810	2.29441e4	323.80475	50.5289		

Figure S106. HPLC profile of racemic **11m**

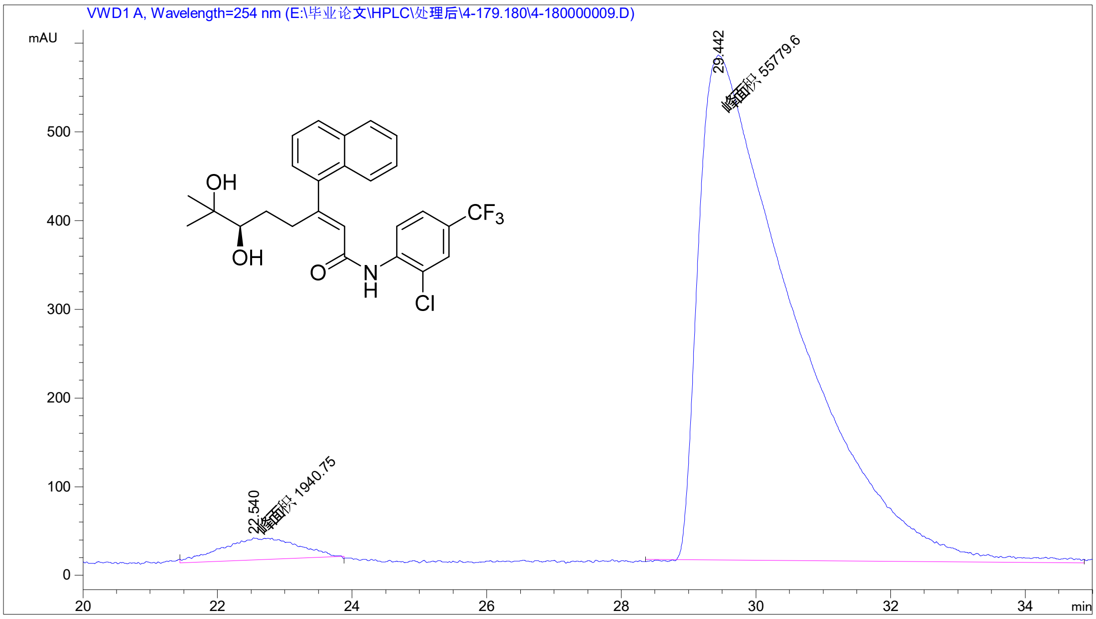

#	[min]		[min]	mAU *s	[mAU]	%
----|-------|----|-------|----------|----------|--------|						
1	22.540	MM	1.3050	1940.74597	24.78526	3.3623
2	29.442	MM	1.6329	5.57796e4	569.32037	96.6377

Figure S107. HPLC profile of (*R*)-**11m**

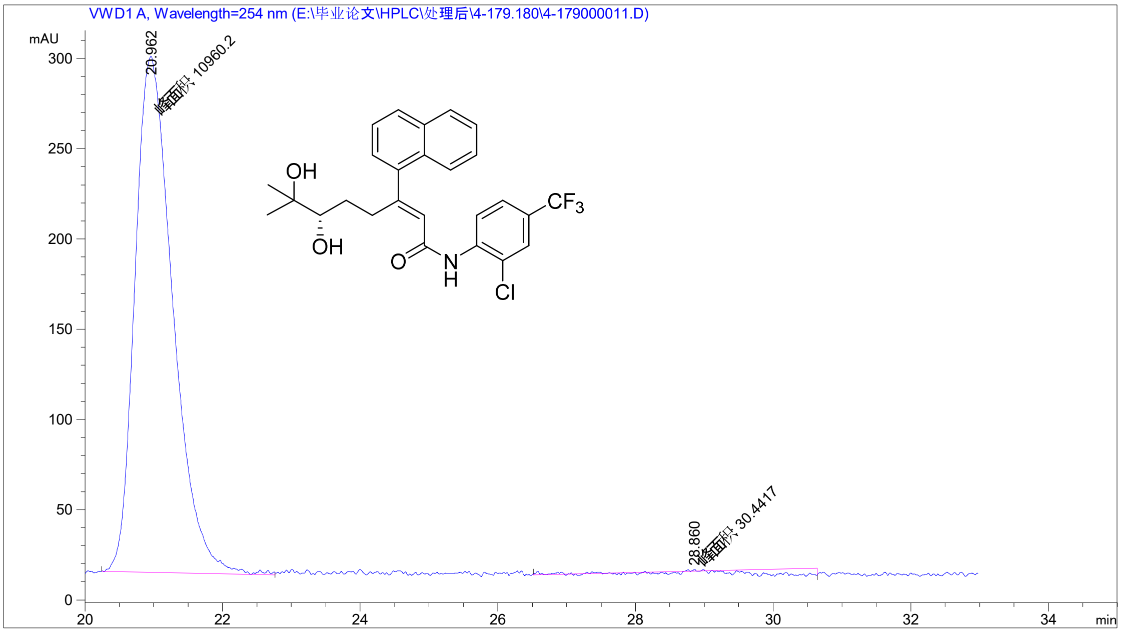

#	[min]		[min]	mAU *s	[mAU]	%
----|-------|----|-------|----------|----------|--------|						
1	20.962	MM	0.6387	1.09602e4	285.99332	99.7230
2	28.860	MM	0.5346	30.44166	9.49025e-1	0.2770

Figure S108. HPLC profile of (*S*)-**11m**

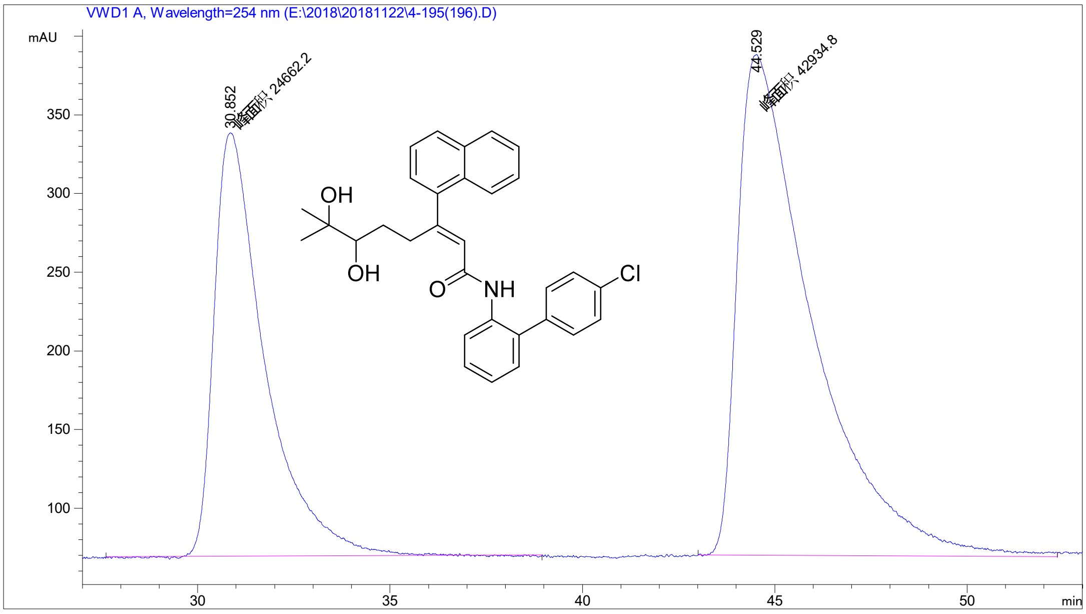

#	[min]		[min]	mAU	*s	[mAU	]	%
1	30.852	MM	1.5274	2.46622e4	269.11304	36.4841		
2	44.529	MM	2.2486	4.29348e4	318.23901	63.5159		

Figure S109. HPLC profile of racemic **11n**

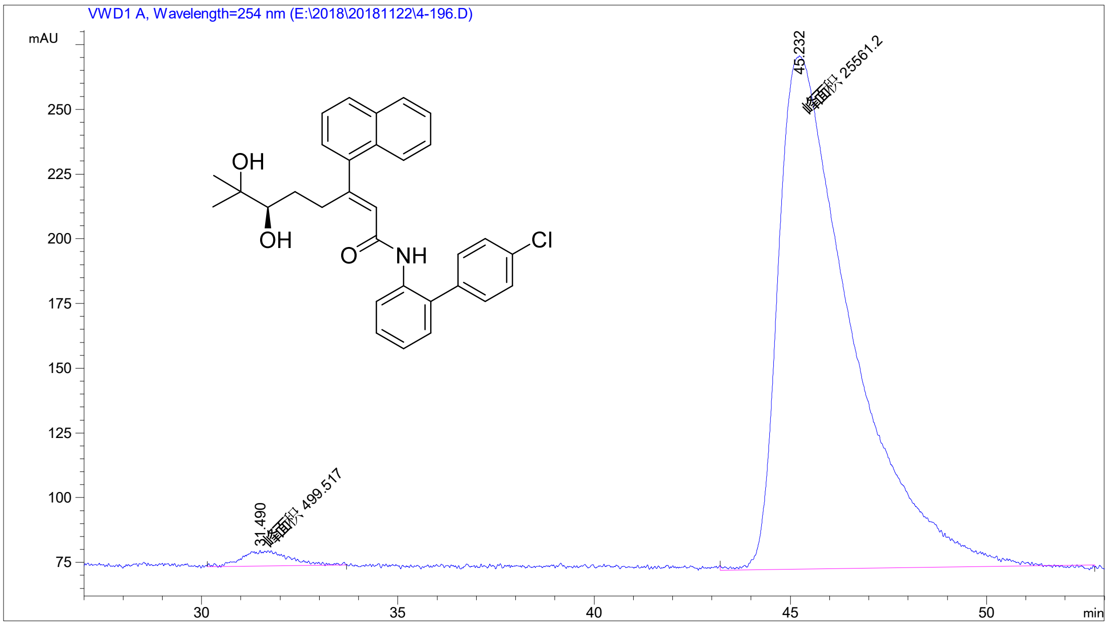

#	[min]		[min]	mAU	*s	[mAU	]	%
1	31.490	MM	1.3693	499.51706	6.07983	1.9167		
2	45.232	MM	2.1490	2.55612e4	198.24382	98.0833		

Figure S110. HPLC profile of (*R*)-**11n**

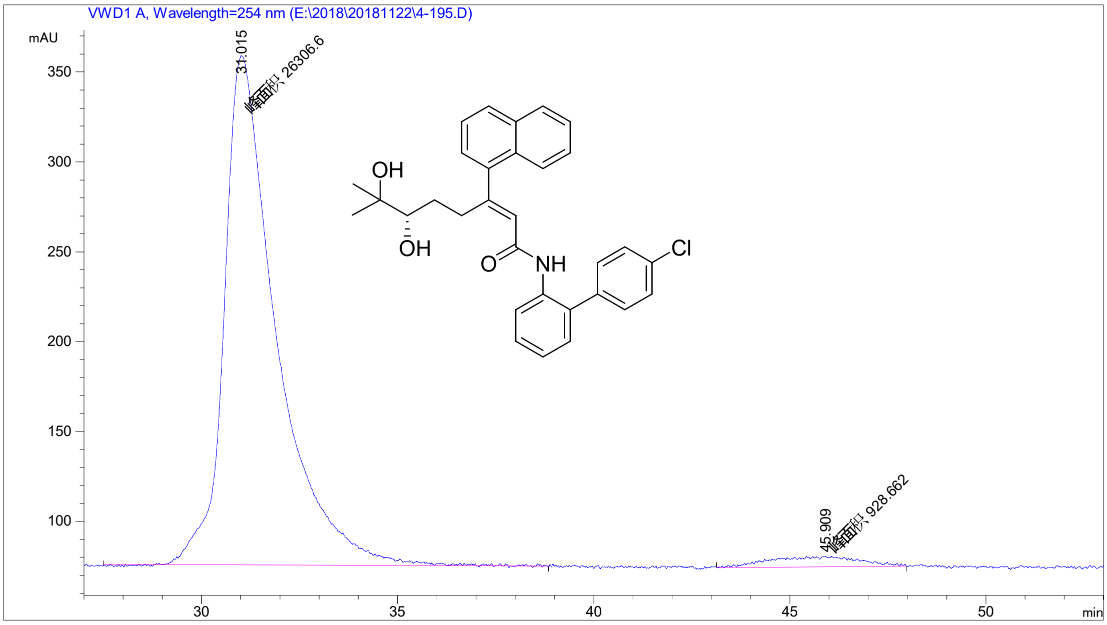

#	[min]		[min]	mAU	*s	[mAU	]	%
----|	-------|	----|	-------|	----------|	----------|	--------|		
1	31.015	MM	1.5474	2.63066e4	283.33441	96.5902		
2	45.909	MM	2.5926	928.66193	5.96991	3.4098		

Figure S111. HPLC profile of (*S*)-**11n**
